# Supplementary material for: Species Traits Drive Long-Term Population Trends of Common Breeding Birds in Northern Italy
Source: Animals (Basel). 2021 Dec 1;11(12):3426. doi: 10.3390/ani11123426 (PMC8698188; doi:10.3390/ani11123426)
Supplement: Supplementary file 1 [file animals-11-03426-s001.zip › SM/Supplementary Material_latest version-update.pdf]

Species traits drive long-term population trends of common breeding birds in Northern Italy

Pietro Tirozzi [1], Valerio Orioli [1], Olivia Dondina [1], Leila Kataoka [1], Luciano Bani [1,2]*

1 Department of Earth and Environmental Sciences, University of Milano-Bicocca, Piazza della Scienza 1, 20126 Milano, Italy; p.tirozzi@campus.unimib.it; valerio.orioli@unimib.it; olivia.dondina@unimib.it; l.kataoka@campus.unimib.it;
2 World Biodiversity Association Onlus c/o NATLAB Forte Inglese, Portoferraio, 57037 Livorno, Italy;
* Correspondence: luciano.bani@unimib.it

Supplementary Materials

List of Contents

Table S1. Number of point counts surveyed per year.

Table S2. Monitoring programs from which bird data were derived.

Table S3. Life history and ecological traits of the studied species.

Figure S1. Cramer's V coefficients of the traits originally considered in the analysis.

Table S4. Explained deviance (%) and the Akaike Information Criterion (AIC) of the models performed for the 76 studied species.

Table S5. Summary of models performed for each species.

Figure S2. Plot of population indices with confidence intervals and regression line for the 76 studied species.

Table S1. Number of point counts surveyed per year.

Year of survey	N of point counts
1992	373
1993	-
1994	-
1995	650
1996	696
1997	-
1998	-
1999	1103
2000	1443
2001	993
2002	593
2003	603
2004	401
2005	1092
2006	1051
2007	730
2008	704
2009	758
2010	744
2011	898
2012	766
2013	795
2014	886
2015	805
2016	799
2017	542
2018	538
2019	542

Table S2. Monitoring programs from which bird data were derived.

Project name	Project goal	Years	Sampling design	Number of point counts
Long-term Monitoring Program	Long-term survey at regional scale	1992, 1995-1996, 2005-2006	Stratified random sampling according to the different landscapes in the Region.	2756
		2007-2016	Stratified random sampling with random and fixed sampling units according to the different landscapes in the Region.	7885
		2017-2019	Stratified random sampling with fixed sampling units according to the different landscapes in the Region.	1622
Forest Biodiversity Survey	Survey of forest habitats	1999-2004	Random sampling in woodlands.	1903
Lowland Biodiversity Survey	Survey of agricultural habitats	2000-2002	Random sampling in agricultural lands.	952
Regional Fauna Database	Survey at regional scale	2000-2006	Systematic stratified sampling at regional scale.	3240
Greenway Project	Survey of Lombard Apennines to draw a greenway	2002-2003	Random sampling along river corridors in the Apennine zone.	147

Table S3. Life history and ecological traits of the studied species. For definition of levels and tresholds of each trait, see Section 2.4.1 in the main text. For traits not used in trait-based analysis: Fledging period (Short: ≤ 14, Intermediate: > 14 and < 26, Long: ≥ 26) and Body mass (Low: ≤ 15.64, Intermediate: > 15.64 and < 149.10, High: ≥ 149.10) were derived from [90]. Nesting habitat was derived from [92] and expert-based revised in relation to preferences of the species in the study area.

Species	Dispersal ratio	Annual fecundity	Incubation period	Fledging period	Migration strategy	Diet	Nest type	Altitudinal range (meters a.s.l.)	Landscape type	Nesting habitat	Overall specialization index	Body mass
Great Cormorant (*Phalacrocorax carbo*)	Low	Low	Long	Long	Sedentary	Vertebrates	Elevated-nester	Plain (≤ 200)	Farmland	Water	High	High
Black-crowned Night Heron (*Nycticorax nycticorax*)	High	Low	Long	Long	Long-distance	Vertebrates	Elevated-nester	Plain (≤ 200)	Farmland	Water	Intermediate	High
Little Egret (*Egretta garzetta*)	High	Low	Long	Long	Short-distance	Omnivores	Elevated-nester	Plain (≤ 200)	Farmland	Water	Intermediate	High
Grey Heron (*Ardea cinerea*)	High	Low	Long	Long	Short-distance	Vertebrates	Elevated-nester	Plain (≤ 200)	Farmland	Water	Intermediate	High
Mallard (*Anas platyrhynchos*)	Low	High	Long	Long	Short-distance	Omnivores	Ground-nester	Plain (≤ 200)	Farmland	Several	Low	High
Black Kite (*Milvus migrans*)	High	Low	Long	Long	Long-distance	Vertebrates	Elevated-nester	Hill (201-700)	Several	Forest	Intermediate	High
Common Buzzard (*Buteo buteo*)	High	Low	Long	Long	Short-distance	Vertebrates	Elevated-nester	Hill (201-700)	Several	Forest	Intermediate	High
Common Kestrel (*Falcon tinnunculus*)	High	Low	Long	Long	Short-distance	Vertebrates	Hole-nester	Plain (≤ 200)	Farmland	Several	Low	High
Common Quail (*Coturnix coturnix*)	Low	Intermediate	Long	Intermediate	Long-distance	Plant-eaters	Ground-nester	Plain (≤ 200)	Farmland	Open	High	Intermediate

Common Pheasant (*Phasianus colchicus*)	Low	High	Long	Short	Sedentary	Plant-eaters	Ground-nester	Plain (≤ 200)	Farmland	Forest	High	High
Common Moohren (*Gallinula chloropus*)	Low	High	Long	Long	Short-distance	Omnivores	Ground-nester	Plain (≤ 200)	Farmland	Water	Intermediate	High
Northern Lapwing (*Vanellus vanellus*)	High	Low	Long	Long	Short-distance	Invertebrates	Ground-nester	Plain (≤ 200)	Farmland	Open	High	High
Feral Pigeon (*Columba livia domestica*)	Intermediate	Intermediate	Long	Long	Sedentary	Plant-eaters	Hole-nester	Plain (≤ 200)	Farmland	Several	High	High
Common Wood Pigeon (*Columba palumbus*)	Intermediate	Low	Intermediate	Long	Short-distance	Plant-eaters	Elevated-nester	Plain (≤ 200)	Farmland	Several	Intermediate	High
Eurasian Collared Dove (*Streptopelia decaocto*)	Intermediate	Intermediate	Intermediate	Intermediate	Sedentary	Plant-eaters	Elevated-nester	Plain (≤ 200)	Farmland	Urban	High	High
European Turtle Dove (*Streptopelia turtur*)	Intermediate	Low	Intermediate	Intermediate	Long-distance	Plant-eaters	Elevated-nester	Plain (≤ 200)	Farmland	Shrubs	High	Intermediate
Common Cuckoo (*Cuculus canorus*)	High	Intermediate	Short	Intermediate	Long-distance	Invertebrates	Elevated-nester	Hill (201-700)	Several	Several	Intermediate	Intermediate
Common Swift (*Apus apus*)	High	Low	Long	Long	Long-distance	Invertebrates	Hole-nester	Plain (≤ 200)	Several	Urban	High	Intermediate
European Bee-eater (*Merops apiaster*)	High	Intermediate	Long	Long	Long-distance	Invertebrates	Hole-nester	Plain (≤ 200)	Farmland	Water	High	Intermediate
Eurasian Wryneck (*Jinx torquilla*)	Low	Intermediate	Short	Intermediate	Long-distance	Invertebrates	Hole-nester	Hill (201-700)	Farmland	Open	High	Intermediate

European Green Woodpecker (*Picus viridis*)	Low	Intermediate	Long	Intermediate	Sedentary	Invertebrates	Hole-nester	Hill (201-700)	Several	Forest	Intermediate	High
Great Spotted Woodpecker (*Dendrocopos major*)	Intermediate	Intermediate	Short	Intermediate	Sedentary	Omnivores	Hole-nester	Hill (201-700)	Several	Forest	Intermediate	Intermediate
Eurasian Skylark (*Alauda arvensis*)	Intermediate	High	Short	Intermediate	Short-distance	Omnivores	Ground-nester	Plain (≤ 200)	Farmland	Open	Intermediate	Intermediate
Eurasian Crag Martin (*Ptyonoprogne rupestris*)	High	Intermediate	Intermediate	Long	Sedentary	Invertebrates	Hole-nester	Low Mountain (701-1500)	Woodland	Urban	High	Intermediate
Barn Swallow (*Hirundo rustica*)	High	High	Intermediate	Intermediate	Long-distance	Invertebrates	Hole-nester	Plain (≤ 200)	Farmland	Open	High	Intermediate
Common House Martin (*Delichon urbicum*)	High	Intermediate	Intermediate	Long	Long-distance	Invertebrates	Hole-nester	Plain (≤ 200)	Several	Urban	High	Intermediate
Tree Pipit (*Anthus trivialis*)	Intermediate	Intermediate	Short	Short	Long-distance	Omnivores	Ground-nester	High Mountain (>1500)	Several	Shrubs	Low	Intermediate
Water Pipit (*Anthus spinoletta*)	Intermediate	Intermediate	Intermediate	Intermediate	Short-distance	Invertebrates	Ground-nester	High Mountain (>1500)	Natural Open-Habitat	Open	High	Intermediate
Western Yellow Wagtail (*Motacilla flava*)	Intermediate	Low	Short	Intermediate	Long-distance	Invertebrates	Ground-nester	Plain (≤ 200)	Farmland	Open	Intermediate	Intermediate
Grey Wagtail (*Motacilla cinerea*)	Intermediate	Intermediate	Short	Short	Short-distance	Invertebrates	Hole-nester	Low Mountain (701-1500)	Several	Water	Intermediate	Intermediate
White Wagtail (*Motacilla alba*)	Intermediate	High	Short	Short	Short-distance	Invertebrates	Hole-nester	Hill (201-700)	Several	Several	Low	Intermediate
Eurasian Wren (*Troglodytes troglodytes*)	Low	High	Intermediate	Intermediate	Short-distance	Omnivores	Hole-nester	Low Mountain (701-1500)	Woodland	Forest	Intermediate	Low

Dunnock (*Prunella modularis*)	Low	Intermediate	Short	Short	Short-distance	Omnivores	Elevated-nester	High Mountain (> 1500)	Natural Open-Habitat	Shrubs	Intermediate	Intermediate
European Robin (*Erithacus rubecula*)	Low	Intermediate	Intermediate	Short	Short-distance	Omnivores	Hole-nester	Low Mountain (701-1500)	Woodland	Forest	Low	Intermediate
Common Nigthingale (*Luscinia megarhynchos*)	Intermediate	Intermediate	Short	Short	Long-distance	Invertebrates	Ground-nester	Plain (≤ 200)	Farmland	Shrubs	Low	Intermediate
Black Redstart (*Phoenicurus ochruros*)	High	Intermediate	Intermediate	Intermediate	Short-distance	Omnivores	Hole-nester	High Mountain (> 1500)	Natural Open-Habitat	Open	High	Intermediate
Common Redstart (*Phoenicurus Phoenicurus*)	Intermediate	High	Short	Intermediate	Long-distance	Invertebrates	Hole-nester	Hill (201-700)	Woodland	Several	Low	Intermediate
African Stonechat (*Saxicola torquatus*)	Low	High	Intermediate	Short	Short-distance	Invertebrates	Ground-nester	Plain (≤ 200)	Farmland	Open	High	Low
Northern Wheatear (*Oenanthe oenanthe*)	High	Intermediate	Short	Intermediate	Long-distance	Invertebrates	Hole-nester	High Mountain (> 1500)	Natural Open-Habitat	Open	Intermediate	Intermediate
Common Blackbird (*Turdus merula*)	Low	Intermediate	Short	Short	Sedentary	Omnivores	Elevated-nester	Hill (201-700)	Several	Several	Low	Intermediate
Song Thrush (*Turdus philomelos*)	Low	Intermediate	Short	Short	Short-distance	Omnivores	Elevated-nester	Low Mountain (701-1500)	Woodland	Forest	Intermediate	Intermediate
Mistle Trush (*Turdus viscivorus*)	Intermediate	Intermediate	Intermediate	Short	Short-distance	Omnivores	Elevated-nester	High Mountain (> 1500)	Several	Shrubs	Intermediate	Intermediate
Cetti's Warbler (*Cettia cetti*)	Low	Intermediate	Intermediate	Intermediate	Sedentary	Invertebrates	Elevated-nester	Plain (≤ 200)	Farmland	Shrubs	Intermediate	Low
Melodius Warbler (*Hippolais polyglotta*)	Intermediate	Low	Short	Short	Long-distance	Invertebrates	Elevated-nester	Plain (≤ 200)	Farmland	Shrubs	Low	Low

Lesser Whitethroat (*Curruca curruca*)	Intermediate	Low	Short	Short	Long-distance	Omnivores	Elevated-nester	High Mountain (> 1500)	Natural Open-Habitat	Shrubs	Low	Low
Eurasian Blackcap (*Sylvia atricapilla*)	Low	Low	Short	Short	Short-distance	Omnivores	Elevated-nester	Hill (201-700)	Several	Several	Intermediate	Intermediate
Western Bonelli's Warbler (*Phylloscopus bonelli*)	Intermediate	Intermediate	Short	Short	Long-distance	Invertebrates	Ground-nester	Low Mountain (701-1500)	Woodland	Shrubs	Intermediate	Low
Common Chiffchaff (*Phylloscopus collybita*)	Intermediate	High	Intermediate	Intermediate	Short-distance	Invertebrates	Ground-nester	Low Mountain (701-1500)	Woodland	Forest	Intermediate	Low
Goldcrest (*Regulus regulus*)	Intermediate	High	Intermediate	Intermediate	Short-distance	Invertebrates	Elevated-nester	Low Mountain (701-1500)	Woodland	Forest	High	Low
Common Firecrest (*Regulus ignicapilla*)	Intermediate	High	Intermediate	Intermediate	Short-distance	Invertebrates	Elevated-nester	Low Mountain (701-1500)	Woodland	Forest	Intermediate	Low
Spotted Flycatcher (*Muscicapa striata*)	High	Intermediate	Short	Short	Long-distance	Invertebrates	Elevated-nester	Hill (201-700)	Several	Several	Intermediate	Intermediate
Long-tailed Tit (*Aegithalos caudatus*)	Intermediate	Intermediate	Intermediate	Intermediate	Sedentary	Omnivores	Elevated-nester	Hill (201-700)	Several	Forest	Intermediate	Low
Marsh Tit (*Poecile palustris*)	Intermediate	Intermediate	Intermediate	Intermediate	Sedentary	Omnivores	Hole-nester	Hill (201-700)	Woodland	Forest	Intermediate	Low
Willow Tit (*Poecile montanus*)	Low	Intermediate	Intermediate	Intermediate	Sedentary	Omnivores	Hole-nester	High Mountain (> 1500)	Natural Open-Habitat	Shrubs	Intermediate	Low
European Crested Tit (*Lophophanes cristatus*)	Intermediate	Intermediate	Intermediate	Intermediate	Sedentary	Omnivores	Hole-nester	Low Mountain (701-1500)	Woodland	Forest	High	Low

Coal Tit (*Periparus ater*)	Intermediate	High	Intermediate	Intermediate	Sedentary	Omnivores	Hole-nester	Low Mountain (701-1500)	Woodland	Forest	Intermediate	Low
Eurasian Blue Tit (*Cyanistes caeruleus*	Intermediate	High	Intermediate	Intermediate	Sedentary	Omnivores	Hole-nester	Hill (201-700)	Woodland	Forest	Intermediate	Low
Great Tit (*Parus major*)	Intermediate	High	Intermediate	Intermediate	Sedentary	Omnivores	Hole-nester	Hill (201-700)	Several	Several	Intermediate	Intermediate
Eurasian Nuthatch (*Sitta europea*)	Intermediate	Intermediate	Intermediate	Intermediate	Sedentary	Invertebrates	Hole-nester	Hill (201-700)	Woodland	Forest	Intermediate	Intermediate
Short-toed Treecreeper (*Certhia brachydactyla*)	Intermediate	High	Intermediate	Intermediate	Sedentary	Invertebrates	Hole-nester	Hill (201-700)	Woodland	Forest	Intermediate	Low
Eurasian Golden Oriole (*Oriolus oriolus*)	High	Low	Intermediate	Intermediate	Long-distance	Omnivores	Elevated-nester	Plain (≤ 200)	Farmland	Forest	Low	Intermediate
Red-backed Shrike (*Lanius collurio*)	Intermediate	Low	Intermediate	Intermediate	Long-distance	Omnivores	Elevated-nester	Hill (201-700)	Several	Shrubs	Intermediate	Intermediate
Eurasian Jay (*Garrulus glandarius*)	Intermediate	Intermediate	Intermediate	Long	Sedentary	Omnivores	Elevated-nester	Hill (201-700)	Woodland	Forest	Low	High
Eurasian Magpie (*Pica pica*)	Intermediate	Intermediate	Long	Long	Sedentary	Omnivores	Elevated-nester	Plain (≤ 200)	Farmland	Several	Low	High
Carrion Crow (*Corvus corone*)	High	Low	Long	Long	Sedentary	Omnivores	Elevated-nester	High Mountain (> 1500)	Natural Open-Habitat	Several	Low	High
Hooded Crow (*Corvus cornix*)	High	Low	Long	Long	Sedentary	Omnivores	Elevated-nester	Plain (≤ 200)	Farmland	Several	Low	High
Common Starling (*Sturnus vulgaris*)	Intermediate	Intermediate	Short	Intermediate	Short-distance	Omnivores	Hole-nester	Plain (≤ 200)	Farmland	Several	Intermediate	Intermediate

Italian Sparrow (*Passer italiae*)	Low	High	Short	Short	Sedentary	Plant-eaters	Hole-nester	Plain (≤ 200)	Farmland	Urban	Low	Intermediate
Eurasian Tree Sparrow (*Passer montanus*)	Low	High	Short	Intermediate	Short-distance	Omnivores	Hole-nester	Plain (≤ 200)	Farmland	Shrubs	Intermediate	Intermediate
Common Chaffinch (*Fringilla coelebs*)	Intermediate	Low	Short	Short	Short-distance	Omnivores	Elevated-nester	Hill (201-700)	Several	Several	Low	Intermediate
European Serin (*Serinus serinus*)	Intermediate	Intermediate	Short	Intermediate	Short-distance	Plant-eaters	Elevated-nester	Hill (201-700)	Several	Urban	Intermediate	Low
European Greenfinch (*Chloris chloris*)	Intermediate	Intermediate	Short	Short	Short-distance	Plant-eaters	Elevated-nester	Plain (≤ 200)	Farmland	Urban	Intermediate	Intermediate
European Goldfinch (*Carduelis carduelis*)	Intermediate	Intermediate	Short	Intermediate	Short-distance	Plant-eaters	Elevated-nester	Plain (≤ 200)	Farmland	Several	Intermediate	Low
Common Linnet (*Linaria cannabina*)	Intermediate	High	Short	Short	Sedentary	Plant-eaters	Elevated-nester	High Mountain (> 1500)	Natural Open-Habitat	Shrubs	High	Intermediate
Common Redpoll (*Acanthis flammea*)	Intermediate	Intermediate	Short	Short	Sedentary	Plant-eaters	Elevated-nester	High Mountain (> 1500)	Natural Open-Habitat	Shrubs	Low	Low
Eurasian Bullfinch (*Pyrrhula pyrrhula*)	Low	Intermediate	Short	Intermediate	Short-distance	Plant-eaters	Elevated-nester	Low Mountain (701-1500)	Woodland	Forest	Low	Intermediate

Figure S1. Cramer's V coefficients of the traits originally considered in the analysis. Statistically significant correlations (Chi-square test or Fisher's exact test) are in bold.

	Dispersal ratio	Annual fecundity	Incubation period	Fledging period	Migration strategy	Diet	Nest type	Altitudinal range	Landscape type	Nesting habitat	Overall specialization index	Body mass
Dispersal ratio	1											
Annual fecundity	**0.31**	1										
Incubation period	**0.36**	**0.31**	1									
Fledging period	**0.41**	**0.33**	**0.63**	1								
Migration strategy	**0.26**	0.23	0.20	0.10	1							
Diet	**0.36**	**0.36**	**0.38**	**0.35**	**0.35**	1						
Nest type	0.15	**0.34**	0.17	0.21	0.21	0.29	1					
Altitudinal	0.19	0.24	**0.34**	**0.32**	0.23	0.23	0.24	1				
Landscape type	0.21	**0.31**	**0.39**	0.28	0.18	0.23	0.25	**0.81**	1			
Nesting habitat	0.31	0.25	**0.47**	**0.50**	**0.33**	**0.38**	**0.40**	**0.50**	**0.50**	1		
Overall specialization index	0.13	0.10	**0.27**	**0.30**	0.16	**0.29**	0.18	0.26	0.21	**0.44**	1	
Body mass	**0.31**	**0.33**	**0.61**	**0.55**	**0.27**	**0.39**	0.19	**0.34**	**0.39**	**0.44**	0.16	1

Table S4. Explained deviance (%) and the Akaike Information Criterion (AIC) of the models performed for the 76 species under study. For zero-inflated GAMs are shown the deviance explained by the two components of the model. C = model with covariates dealing with environmental bias; ZIP = zero-inflated Poisson; ZINB = zero-inflated negative binomial; P = Poisson; NB = negative binomial; GAM = generalized additive model. For negative binomial models, values of the estimated dispersion parameter θ (the smaller θ, the larger overdispersion) are indicated in parentheses. High values of θ indicates the absence of overdispersion.

Species	Model	AIC	Explained deviance (%)	
Great Cormorant (*Phalacrocorax carbo*)	C-NB-GAM (0.284)	3029.39		57.60
	C-ZINB-GAM (0.736)	3076.70	Binomial	77.90
			Count	54.80
	C-ZIP-GAM	3231.56	Binomial	61.80
			Count	59.70
	C-P-GAM	3395.69		54.70
Black-crowned Night Heron (*Nycticorax nycticorax*)	C-NB-GAM (0.608)	6480.73		54.80
	C-ZINB-GAM (1.179)	6537.11	Binomial	89.00
			Count	42.40
	C-ZIP-GAM	6730.28	Binomial	78.00
			Count	45.40
	C-P-GAM	6921.94		49.60
Little Egret (*Egretta garzetta*)	C-NB-GAM (0.754)	9928.53		57.60
	C-ZINB-GAM (1.092)	9939.50	Binomial	92.40
			Count	42.20
	C-ZIP-GAM	10388.91	Binomial	73.30
			Count	47.10
	C-P-GAM	10690.01		52.30
Grey Heron (*Ardea cinerea*)	C-NB-GAM (1.668)	13735.41		43.20
	C-ZINB-GAM (2.020)	13754.99	Binomial	93.00
			Count	24.20
	C-ZIP-GAM	14081.95	Binomial	84.30
			Count	25.30
	C-P-GAM	14134.81		40.30
Mallard (*Anas platyrhynchos*)	C-ZINB-GAM (0.334)	11911.70	Binomial	83.00
			Count	7.30
	C-NB-GAM (0.184)	11951.06		45.00
	C-P-GAM	16149.18		34.40
	C-ZIP-GAM	Not converged		
Black Kite (*Milvus migrans*)	C-ZINB-GAM (1.595)	3533.71	Binomial	60.30
			Count	21.80
	C-ZIP-GAM	3554.90	Binomial	49.60
			Count	27.30
	C-NB-GAM (0.236)	3563.95		25.50
	C-P-GAM	3708.34		22.40
Common Buzzard (*Buteo buteo*)	C-P-GAM	6141.11		10.90
	C-ZIP-GAM	6155.44	Binomial	50.70
			Count	9.10
	C-NB-GAM (1144.979)	6173.12		9.98
	C-ZINB-GAM (92409.470)	6173.28	Binomial	54.80
			Count	7.74

Common Kestrel (*Falcon tinnunculus*)	C-P-GAM	6927.50		12.80
	C-NB-GAM (299509.437)	6929.30		12.70
	C-ZIP-GAM	7031.40	Binomial	46.20
			Count	10.90
	C-ZINB-GAM (295104.699)	7045.20	Binomial	48.80
			Count	10.40
Common Quail (*Coturnix coturnix*)	C-ZIP-GAM	4384.35	Binomial	62.40
			Count	30.90
	C-NB-GAM (0.805)	4406.25		30.70
	C-ZINB-GAM (71374.331)	4407.33	Binomial	61.90
			Count	29.90
	C-P-GAM	4432.33		28.10
Common Pheasant (*Phasianus colchicus*)	C-NB-GAM (1.884)	11672.84		38.90
	C-ZIP-GAM	11699.55	Binomial	73.20
			Count	29.50
	C-ZINB-GAM (9.601)	11719.96	Binomial	74.50
			Count	28.70
	C-P-GAM	11768.93		37.50
Common Moohren (*Gallinula chloropus*)	C-NB-GAM (3.303)	8059.16		42.00
	C-P-GAM	8069.28		40.90
	C-ZIP-GAM	8082.19	Binomial	88.80
			Count	29.90
	C-ZINB-GAM (18.068)	8096.48	Binomial	89.30
			Count	29.70
Northern Lapwing (*Vanellus vanellus*)	C-NB-GAM (0.315)	3496.99		68.80
	C-ZINB-GAM (0.675)	3514.25	Binomial	92.20
			Count	57.10
	C-ZIP-GAM	3602.08	Binomial	81.80
			Count	63.30
	C-P-GAM	3968.96		61.00
Feral Pigeon (*Columba livia domestica*)	C-ZINB-GAM (0.473)	26373.81	Binomial	89.30
			Count	24.50
	C-NB-GAM (0.380)	26547.21		47.00
	C-ZIP-GAM	33086.27	Binomial	29.00
			Count	36.70
	C-P-GAM	39737.77		36.90
Common Wood Pigeon (*Columba palumbus*)	C-ZIP-GAM	16727.40	Binomial	73.30
			Count	23.30
	C-ZINB-GAM (15.744)	16751.67	Binomial	75.00
			Count	22.60
	C-NB-GAM	16791.70		29.70
	C-P-GAM	16829.58		29.10
Eurasian Collared Dove (*Streptopelia decaocto*)	C-ZINB-GAM (4.220)	23900.66	Binomial	85.50
			Count	37.80
	C-NB-GAM (2.668)	24021.06		47.70
	C-ZIP-GAM	24057.30	Binomial	74.60
			Count	38.50
	C-P-GAM	24405.06		46.00
European Turtle Dove (*Streptopelia turtur*)	C-ZIP-GAM	12102.23	Binomial	70.10
			Count	28.60
	C-ZINB-GAM (9.848)	12110.22	Binomial	71.50
			Count	28.20
	C-NB-GAM (1.318)	12185.88		33.60
	C-P-GAM	12326.01		31.70

Common Cuckoo (*Cuculus canorus*)	C-ZIP-GAM	25792.68	Binomial	80.70
			Count	14.70
	C-ZINB-GAM (336562.339)	25863.40	Binomial	80.10
			Count	14.10
	C-P-GAM	26140.75		19.00
	C-NB-GAM (3059509.190)	26180.97		18.70
Common Swift (*Apus apus*)	C-ZINB-GAM (0.295)	40080.10	Binomial	54.80
			Count	9.61
	C-NB-GAM (0.178)	40556.48		14.40
	C-ZIP-GAM	55397.13	Binomial	7.87
			Count	8.44
	C-P-GAM	79149.04		14.80
European Bee-eater (*Merops apiaster*)	C-NB-GAM (0.073)	4230.85		48.40
	C-ZINB-GAM (0.376)	4286.48	Binomial	45.70
			Count	42.30
	C-ZIP-GAM	4475.40	Binomial	14.30
			Count	55.90
	C-P-GAM	5468.65		37.30
Eurasian Wryneck (*Jynx torquilla*)	C-ZIP-GAM	3713.88	Binomial	63.40
			Count	28.00
	C-ZINB-GAM (66017.577)	3753.80	Binomial	64.10
			Count	26.60
	C-P-GAM	3754.60		26.10
	C-NB-GAM (0.346)	4277.52		26.30
European Green Woodpecker (*Picus viridis*)	C-P-GAM	7913.30		17.60
	C-ZIP-GAM	7931.24	Binomial	64.30
			Count	15.90
	C-NB-GAM (15411.061)	7997.53		16.1
	C-ZINB-GAM (905764.043)	8011.81	Binomial	63.80
			Count	13.50
Great Spotted Woodpecker (*Dendrocopos major*)	C-P-GAM	11958.44		20.90
	C-NB-GAM (92666.166)	12021.31		20.20
	C-ZIP-GAM	12023.01	Binomial	79.10
			Count	18.20
	C-ZINB-GAM (207106.000)	12076.00	Binomial	79.60
			Count	17.40
Eurasian Skylark (*Alauda arvensis*)	C-ZIP-GAM	13476.92	Binomial	77.60
			Count	45.00
	C-ZINB-GAM (14.493)	13504.58	Binomial	78.70
			Count	44.50
	C-NB-GAM (2.362)	13667.07		48.30
	C-P-GAM	13742.54		47.00
Eurasian Crag Martin (*Ptyonoprogne rupestris*)	C-NB-GAM (0.159)	3798.65		47.10
	C-ZINB-GAM (1.021)	3833.38	Binomial	62.40
			Count	43.60
	C-ZIP-GAM	3844.65	Binomial	40.00
			Count	49.90
	C-P-GAM	4178.56		39.60
Barn Swallow (*Hirundo rustica*)	C-ZINB-GAM (0.802)	40303.37	Binomial	87.20
			Count	25.20
	C-NB-GAM (0.708)	40464.14		41.00
	C-ZIP-GAM	47492.13	Binomial	31.40
			Count	26.10
	C-P-GAM	51918.72		35.00

Common House Martin (*Delichon urbicum*)	C-ZINB-GAM (0.291)	23845.97	Binomial	60.60
			Count	12.20
	C-NB-GAM (0.154)	24040.40		18.30
	C-ZIP-GAM	27742.22	Binomial	8.39
			Count	16.70
	C-P-GAM	36579.74		17.00
Tree Pipit (*Anthus trivialis*)	C-ZIP-GAM	5021.72	Binomial	93.80
			Count	47.20
	C-ZINB-GAM (259222.884)	5050.91	Binomial	93.60
			Count	46.50
	C-P-GAM	5177.49		65.90
	C-NB-GAM (4.512)	5224.74		66.50
Water Pipit (*Anthus spinoletta*)	C-ZINB-GAM (177199.646)	3707.63	Binomial	95.50
			Count	66.60
	C-P-GAM	3715.80		84.60
	C-NB-GAM (11.8)	3742.13		84.90
	C-ZIP-GAM		Not converged	
Western Yellow Wagtail (*Motacilla flava*)	C-ZIP-GAM	11274.22	Binomial	87.00
			Count	35.60
	C-ZINB-GAM (8.354)	11290.24	Binomial	89.40
			Count	35.00
	C-NB-GAM (2.546)	11421.31		54.40
	C-P-GAM	11501.58		52.10
Grey Wagtail (*Motacilla cinerea*)	C-P-GAM	4879.06		30.40
	C-ZIP-GAM	4896.97	Binomial	87.20
			Count	24.40
	C-NB-GAM (32.599)	4935.47		29.00
	C-ZINB-GAM (119200.423)	4960.33	Binomial	86.90
			Count	22.30
White Wagtail (*Motacilla alba*)	C-P-GAM	11209.53		20.40
	C-NB-GAM (34911.335)	11225.74		20.20
	C-ZIP-GAM	11234.61	Binomial	75.80
			Count	19.90
	C-ZINB-GAM (125329.338)	11264.26	Binomial	75.30
			Count	19.50
Eurasian Wren (*Troglodytes troglodytes*)	C-ZIP-GAM	17495.73	Binomial	90.50
			Count	18.30
	C-ZINB-GAM (96764.546)	17517.74	Binomial	90.10
			Count	18.10
	C-P-GAM	17682.89		39.60
	C-NB-GAM (10.698)	17943.17		38.30
Dunnock (*Prunella modularis*)	C-P-GAM	4894.66		69.90
	C-ZIP-GAM	4902.03	Binomial	96.50
			Count	40.50
	C-NB-GAM (90289.738)	4908.35		69.70
	C-ZINB-GAM (290852.931)	4923.56	Binomial	96.30
			Count	40.00
European Robin (*Erithacus rubecula*)	C-ZIP-GAM	16251.34	Binomial	93.40
			Count	33.30
	C-ZINB-GAM (93303.352)	16273.17	Binomial	93.30
			Count	33.30
	C-P-GAM	16634.23		52.80
	C-NB-GAM (87.877)	16645.47		52.80

Common Nigthingale (*Luscinia megarhynchos*)	C-ZIP-GAM	25515.68	Binomial	89.60
			Count	33.50
	C-ZINB-GAM (213716.954)	25573.05	Binomial	89.20
			Count	33.70
	C-P-GAM	26089.01		52.70
	C-NB-GAM (690471.069)	26114.85		52.50
Black Redstart (*Phoenicurus ochruros*)	C-ZIP-GAM	7593.68	Binomial	88.70
			Count	41.10
	C-ZINB-GAM (387811.573)	7603.81	Binomial	89.00
			Count	40.80
	C-P-GAM	7635.01		56.00
	C-NB-GAM (141339.368)	7636.27		56.00
Common Redstart (*Phoenicurus Phoenicurus*)	C-ZIP-GAM	11928.43	Binomial	88.20
			Count	23.10
	C-ZINB-GAM (385821.722)	11948.16	Binomial	88.10
			Count	22.90
	C-P-GAM	12013.63		34.20
	C-NB-GAM (229589.045)	12021.75		34.10
African Stonechat (*Saxicola torquatus*)	C-ZIP-GAM	5151.91	Binomial	54.40
			Count	33.40
	C-ZINB-GAM (32713.385)	5180.57	Binomial	53.50
			Count	32.40
	C-NB-GAM (0.629)	5181.02		29.10
	C-P-GAM	5224.84		26.50
Northern Wheatear (*Oenanthe oenanthe*)	C-P-GAM	2515.16		77.80
	C-ZIP-GAM	2519.88	Binomial	97.90
			Count	55.00
	C-NB-GAM (41460.755)	2526.04		77.30
	C-ZINB-GAM (762946.519)	2536.48	Binomial	97.90
			Count	54.30
Common Blackbird (*Turdus merula*)	C-ZIP-GAM	45864.32	Binomial	81.20
			Count	22.20
	C-ZINB-GAM (1720556.717)	45714.74	Binomial	81.00
			Count	22.00
	C-P-GAM	45832.06		28.00
	C-NB-GAM (2692926.525)	45851.83		27.80
Song Thrush (*Turdus philomelos*)	C-ZIP-GAM	6540.94	Binomial	92.90
			Count	23.60
	C-ZINB-GAM (129475.547)	6648.63	Binomial	91.90
			Count	20.00
	C-P-GAM	6674.23		44.40
	C-NB-GAM (3.090)	6806.35		43.70
Mistle Thrush (*Turdus viscivorus*)	C-ZIP-GAM	2902.87	Binomial	88.20
			Count	24.30
	C-ZINB-GAM (1001115.204)	2925.33	Binomial	89.50
			Count	19.10
	C-P-GAM	2932.33		46.30
	C-NB-GAM (1.580)	2934.39		48.10

Cetti's Warbler (*Cettia cetti*)	C-ZIP-GAM	6450.23	Binomial	84.50
			Count	37.30
	C-ZINB-GAM (8.695)	6468.52	Binomial	84.60
			Count	37.00
	C-NB-GAM (0.996)	6486.37		46.30
	C-P-GAM	6565.63		43.30
Melodius Warbler (*Hippolais polyglotta*)	C-ZIP-GAM	4529.84	Binomial	65.30
			Count	31.10
	C-NB-GAM (0.435)	4534.72		31.40
	C-ZINB-GAM (17.146)	4555.42	Binomial	64.90
			Count	29.30
	C-P-GAM	4585.81		28.70
Lesser Whitethroat (*Curruca curruca*)	C-ZIP-GAM	3000.43	Binomial	91.80
			Count	31.80
	C-ZINB-GAM (66638.615)	3014.07	Binomial	91.10
			Count	32.20
	C-P-GAM	3020.25		61.30
	C-NB-GAM (1.63)	3042.61		63.10
Eurasian Blackcap (*Sylvia atricapilla*)	C-ZIP-GAM	48060.23	Binomial	88.60
			Count	21.50
	C-ZINB-GAM (595897.002)	48132.52	Binomial	87.20
			Count	20.80
	C-P-GAM	48209.62		29.30
	C-NB-GAM (566535.073)	48282.73		28.80
Western Bonelli's Warbler (*Phylloscopus bonelli*)	C-ZINB-GAM (3.52)	6627.47	Binomial	71.00
			Count	41.40
	C-NB-GAM (0.405)	6726.72		57.80
	C-P-GAM	7211.36		51.50
	C-ZIP-GAM		Not converged	
Common Chiffchaff (*Phylloscopus collybita*)	C-ZIP-GAM	13150.88	Binomial	91.90
			Count	27.70
	C-ZINB-GAM (58861.5429	13172.84	Binomial	91.50
			Count	28.30
	C-P-GAM	13329.43		50.40
	C-NB-GAM (17966.806)	13330.12		50.40
Goldcrest (*Regulus regulus*)	C-ZIP-GAM	5562.70	Binomial	91.80
			Count	47.30
	C-P-GAM	5640.06		59.70
	C-ZINB-GAM (25.475)	5643.97	Binomial	91.30
			Count	45.40
	C-NB-GAM (2.305)	5674.59		60.90
Common Firecrest (*Regulus ignicapilla*)	C-ZIP-GAM	4838.47	Binomial	72.80
			Count	40.30
	C-ZINB-GAM (21117.411)	4900.82	Binomial	67.80
			Count	40.30
	C-NB-GAM (0.671)	4902.20		45.40
	C-P-GAM	4916.71		42.10
Spotted Flycatcher (*Muscicapa striata*)	C-P-GAM	13412.03		21.40
	C-ZIP-GAM	13427.06	Binomial	79.10
			Count	15.80
	C-NB-GAM	13429.56		21.10
	C-ZINB-GAM (1537699.063)	13502.62	Binomial	81.80
			Count	14.20

Long-tailed Tit (*Aegithalos caudatus*)	C-P-GAM	8768.01		17.80
	C-NB-GAM (2531114.871)	8771.23		17.80
	C-ZIP-GAM	8855.56	Binomial	71.00
			Count	12.60
	C-ZINB-GAM (88403.418)	8861.84	Binomial	71.30
			Count	12.40
Marsh Tit (*Poecile palustris*)	C-ZIP-GAM	6839.02	Binomial	86.00
			Count	31.90
	C-ZINB-GAM (30.045)	6877.20	Binomial	86.00
			Count	30.70
	C-NB-GAM (1.056)	7001.02		45.80
	C-P-GAM	7057.28		42.80
Willow Tit (*Poecile montanus*)	C-ZIP-GAM	2750.03	Binomial	89.80
			Count	32.40
	C-ZINB-GAM (255855.435)	2760.22	Binomial	90.00
			Count	30.70
	C-NB-GAM (1.532)	2810.90		58.80
	C-P-GAM	2827.38		55.90
European Crested Tit (*Lophophanes cristatus*)	C-ZIP-GAM	4225.32	Binomial	86.30
			Count	46.30
	C-ZINB-GAM (28.989)	4240.97	Binomial	86.30
			Count	46.00
	C-NB-GAM	4298.49		58.00
	C-P-GAM	4372.76		54.00
Coal Tit (P*eriparus ater*)	C-ZIP-GAM	13899.30	Binomial	89.50
			Count	42.20
	C-ZINB-GAM (26.712)	13939.49	Binomial	90.30
			Count	41.80
	C-NB-GAM (5.177)	14211.60		67.50
	C-P-GAM	14249.30		66.40
Eurasian Blue Tit (*Cyanistes caeruleus*)	C-ZIP-GAM	13764.00	Binomial	79.50
			Count	19.70
	C-ZINB-GAM (166.830)	13844.32	Binomial	78.30
			Count	18.70
	C-P-GAM	13997.30		32.90
	C-NB-GAM ((2.661)	14038.47		33.20
Great Tit (*Parus major*)	C-ZIP-GAM	32209.26	Binomial	81.10
			Count	13.50
	C-ZINB-GAM (122665.837)	32248.47	Binomial	80.50
			Count	13.30
	C-P-GAM	32296.40		21.80
	C-NB-GAM (56747.195)	32317.42		21.60
Eurasian Nuthatch (*Sitta europaea*)	C-ZIP-GAM	4736.48	Binomial	85.30
			Count	32.00
	C-ZINB-GAM (2673.914)	4833.81	Binomial	84.30
			Count	28.60
	C-P-GAM	4887.27		38.50
	C-NB-GAM (0.821)	4914.27		40.40

Short-toed Treecreeper (*Certhia brachydactyla*)	C-ZIP-GAM	2952.57	Binomial	91.30
			Count	36.70
	C-P-GAM	2956.61		45.60
	C-NB-GAM (4.807)	2965.82		46.00
	C-ZINB-GAM (67079.969)	2973.61	Binomial	91.00
			Count	35.70
Eurasian Golden Oriole (*Oriolus oriolus*)	C-ZIP-GAM	9430.02	Binomial	85.30
			Count	30.50
	C-ZINB-GAM (94932.338)	9466.67	Binomial	83.50
			Count	31.40
	C-P-GAM	9514.51		40.40
	C-NB-GAM	9533.47		41.10
Red-backed Shrike (*Lanius collurio*)	C-P-GAM	6147.40		23.20
	C-NB-GAM (7.766)	6153.75		23.20
	C-ZIP-GAM	6172.55	Binomial	69.70
			Count	25.40
	C-ZINB-GAM (131338.51)	6189.26	Binomial	70.30
			Count	25.10
Eurasian Jay (*Garrulus glandarius*)	C-P-GAM	9552.70		29.40
	C-NB-GAM (453507.129)	9578.89		28.90
	C-ZIP-GAM	9589.78	Binomial	85.80
			Count	20.40
	C-ZINB-GAM (132878.23)	9836.27	Binomial	84.30
			Count	16.00
Eurasian Magpie (*Pica pica*)	C-ZIP-GAM	12836.52	Binomial	89.50
			Count	27.70
	C-ZINB-GAM (16.487)	12858.11	Binomial	89.80
			Count	27.40
	C-NB-GAM (4.266)	12882.85		39.50
	C-P-GAM	12897.32		28.70
Carrion Crow (*Corvus corone*)	C-ZIP-GAM	3261.47	Binomial	86.70
			Count	45.70
	C-ZINB-GAM (2.416)	3264.04	Binomial	89.60
			Count	41.50
	C-NB-GAM (0.732)	3348.44		55.00
	C-P-GAM	3391.32		53.00
Hooded Crow (*Corvus cornix*)	C-ZINB-GAM (2.221)	47078.79	Binomial	91.40
			Count	32.30
	C-NB-GAM (2.184)	47142.09		39.20
	C-ZIP-GAM	51689.46	Binomial	75.30
			Count	29.90
	C-P-GAM	51896.28		36.60
Common Starling (*Sturnus vulgaris*)	C-ZINB-GAM (3.064)	32012.92	Binomial	97.10
			Count	32.40
	C-NB-GAM (3.011)	32013.45		48.00
	C-ZIP-GAM	33407.99	Binomial	94.80
			Count	29.90
	C-P-GAM	33442.58		43.70

Italian Sparrow (*Passer italiae*)	C-ZIP-GAM	30881.22	Binomial	97.20
			Count	39.00
	C-ZINB-GAM (21.534)	30913.60	Binomial	96.90
			Count	39.80
	C-P-GAM	30937.87		48.90
	C-NB-GAM (19.285)	30944.07		49.20
Eurasian Tree Sparrow (*Passer montanus*)	C-ZIP-GAM	17891.27	Binomial	94.90
			Count	21.90
	C-P-GAM	17912.57		33.90
	C-ZINB-GAM (658979.465)	17934.73	Binomial	94.50
			Count	21.40
	C-NB-GAM (353604.226)	17953.29		33.50
Common Chaffinch (*Fringilla coelebs*)	C-ZIP-GAM	41633.72	Binomial	79.60
			Count	24.20
	C-ZINB-GAM (3555510.598)	41665.93	Binomial	79.80
			Count	24.00
	C-P-GAM	43408.7		41.10
	C-NB-GAM (785815.051)	43430.87		40.90
European Serin (*Serinus serinus*)	C-ZIP-GAM	15368.22	Binomial	87.80
			Count	26.30
	C-ZINB-GAM (63.969)	15437.27	Binomial	87.50
			Count	25.90
	C-NB-GAM	15962.71		35.20
	C-P-GAM	16008.36		34.40
European Greenfinch (*Chloris chloris*)	C-ZINB-GAM (2.639)	16415.30	Binomial	68.10
			Count	23.10
	C-NB-GAM (0.902)	16485.18		30.10
	C-ZIP-GAM	16532.33	Binomial	57.40
			Count	25.00
	C-P-GAM	17042.48		27.50
European Goldfinch (*Carduelis carduelis*)	C-ZINB-GAM (1.7749)	19064.10	Binomial	52.90
			Count	19.00
	C-NB-GAM (0.623)	19084.07		21.50
	C-ZIP-GAM	19157.66	Binomial	34.40
			Count	23.40
	C-P-GAM	19939.7		20.10
Common Linnet (*Linaria cannabina*)	C-ZINB-GAM (1.336)	3209.39	Binomial	92.10
			Count	39.90
	C-ZIP-GAM	3253.82	Binomial	82.90
			Count	45.90
	C-NB-GAM (0.473)	3275.33		65.90
	C-P-GAM	3458.20		59.20
Common Redpoll (*Acanthis flammea*)	C-ZINB-GAM (1.190)	3421.109	Binomial	94.20
			Count	29.10
	C-ZIP-GAM	3466.635	Binomial	81.70
			Count	41.00
	C-NB-GAM (0.592)	3515.496		69.00
	C-P-GAM	3699.918		62.20

	C-ZIP-GAM	3601.80	Binomial	89.30
			Count	29.30
Eurasian Bullfinch (*Pyrrhula pyrrhula*)	C-P-GAM	3617.81		49.20
	C-NB-GAM (2.084)	3627.82		50.60
	C-ZINB-GAM (87108.682)	3631.22	Binomial	87.60
			Count	28.70

Table S5. Summary of models performed for each species. In each table, the common and the scientific name of the species, the type of model and summary statistics for both parametric and smooth terms are shown. Land cover covariates (see also [54] for further details): C110 = continue urban matrix and infrastructures; C112 = discontinue urban matrix; C211 = arable lands; C213 = paddy fields; C221 = vineyards; C222 = orchards; C223 = olive groves; C224 = wood plantations; C231 = meadows and pastures; C311 = broadleaved forests; C312 = coniferous forests; C313 = mixed forests; C320 = shrub lands; C321 = grasslands; C330 = areas with sparse or absent vegetation; C410 = wetland vegetation; C511 = rivers and streams. Edf = estimated degree of freedom.

**Great Cormorant (*Phalacrocorax carbo*) — Model C-NB-GAM**				
**Parametric terms**	**Estimate**	**Standard error**	**z-value**	**p-value**
Intercept	−7.963	1.052	−7.568	< 0.001
Year 2005	0.459	0.390	1.178	0.239
Year 2006	−0.583	0.620	−0.941	0.347
Year 2007	0.709	0.441	1.607	0.108
Year 2008	0.954	0.403	2.369	0.018
Year 2009	1.698	0.365	4.655	< 0.001
Year 2010	1.540	0.387	3.978	< 0.001
Year 2011	1.899	0.371	5.120	< 0.001
Year 2012	1.453	0.383	3.797	< 0.001
Year 2013	1.709	0.374	4.575	< 0.001
Year 2014	2.574	0.349	7.374	< 0.001
Year 2015	2.743	0.348	7.888	< 0.001
Year 2016	2.240	0.382	5.872	< 0.001
Year 2017	2.208	0.378	5.839	< 0.001
Year 2018	2.382	0.374	6.370	< 0.001
Year 2019	−0.126	0.092	−1.364	0.172
Sin	0.232	0.140	1.661	0.097
Cos	−7.963	1.052	−7.568	< 0.001
**Smooth terms**		**edf**	**Chi square**	**p-value**
s(X,Y)		2.015	242.782	< 0.001
s(Elevation)		2.488	168.363	< 0.001
s(Slope)		0.756	2.814	0.051
s(C110)		2.062	76.111	< 0.001
s(C112)		1.024	28.223	< 0.001
s(C211)		1.502	65.433	< 0.001
s(C213)		1.020	30.178	< 0.001
s(C221)		0.606	1.369	0.129
s(C222)		0.000	0.000	0.645
s(C223)		0.000	0.000	0.369
s(C224)		0.924	11.020	< 0.001
s(C231)		1.095	33.632	< 0.001
s(C311)		2.332	34.278	< 0.001
s(C312)		0.693	1.728	0.114
s(C313)		0.910	8.012	0.002
s(C320)		0.000	0.000	0.747
s(C321)		0.358	0.451	0.259
s(C330)		0.000	0.000	0.396
s(C410)		0.987	43.675	< 0.001
s(C511)		0.622	1.477	0.118

Black-crowned Night Heron (*Nycticorax nycticorax*) — Model C-NB-GAM				
**Parametric terms**	**Estimate**	**Standard error**	**z-value**	**p-value**
Intercept	−7.401	1.021	−7.247	< 0.001
Year 1995	−2.113	0.289	−7.302	< 0.001
Year 1996	−1.349	0.226	−5.956	< 0.001
Year 1999	−1.293	0.223	−5.810	< 0.001
Year 2000	−0.766	0.195	−3.923	< 0.001
Year 2001	−1.083	0.201	−5.375	< 0.001
Year 2002	−1.883	0.272	−6.922	< 0.001
Year 2003	−2.128	0.320	−6.640	< 0.001
Year 2004	−1.573	0.331	−4.745	< 0.001
Year 2005	−1.655	0.216	−7.646	< 0.001
Year 2006	−1.557	0.228	−6.814	< 0.001
Year 2007	−1.486	0.240	−6.187	< 0.001
Year 2008	−1.230	0.230	−5.353	< 0.001
Year 2009	−1.734	0.237	−7.304	< 0.001
Year 2010	−0.921	0.210	−4.376	< 0.001
Year 2011	−1.282	0.225	−5.700	< 0.001
Year 2012	−2.048	0.272	−7.539	< 0.001
Year 2013	−1.329	0.232	−5.729	< 0.001
Year 2014	−1.261	0.232	−5.440	< 0.001
Year 2015	−1.250	0.226	−5.540	< 0.001
Year 2016	−1.703	0.249	−6.836	< 0.001
Year 2017	−1.621	0.305	−5.323	< 0.001
Year 2018	−1.914	0.296	−6.457	< 0.001
Year 2019	−2.487	0.378	−6.573	< 0.001
Sin	0.091	0.056	1.610	0.107
Cos	−0.076	0.086	−0.880	0.379
**Smooth terms**		**edf**	**Chi square**	**p-value**
s(X,Y)		1.954	114.550	< 0.001
s(Elevation)		1.017	58.680	< 0.001
s(Slope)		0.284	0.355	0.257
s(C110)		1.016	19.906	< 0.001
s(C112)		0.122	0.143	0.275
s(C211)		0.992	7.735	0.003
s(C213)		2.681	216.444	< 0.001
s(C221)		0.798	3.332	0.040
s(C222)		0.865	5.404	0.012
s(C223)		0.688	0.418	0.436
s(C224)		0.011	0.009	0.351
s(C231)		0.914	7.470	0.003
s(C311)		0.001	0.001	0.166
s(C312)		0.000	0.000	0.696
s(C313)		0.000	0.000	0.577
s(C320)		0.002	0.001	0.392
s(C321)		0.631	1.035	0.200
s(C330)		0.501	0.865	0.187
s(C410)		1.007	132.680	< 0.001
s(C511)		0.000	0.000	0.423

Little Egret (*Egretta garzetta*) — Model C-NB-GAM				
**Parametric terms**	**Estimate**	**Standard error**	**z-value**	**p-value**
Intercept	−11.318	1.510	−7.497	< 0.001
Year 1995	−0.667	0.272	−2.452	0.014
Year 1996	−0.386	0.253	−1.526	0.127
Year 1999	0.282	0.234	1.204	0.229
Year 2000	0.722	0.220	3.276	0.001
Year 2001	0.211	0.225	0.938	0.348
Year 2002	0.399	0.244	1.635	0.102
Year 2003	−0.416	0.278	−1.497	0.134
Year 2004	−0.391	0.325	−1.201	0.230
Year 2005	0.087	0.228	0.381	0.703
Year 2006	0.245	0.231	1.061	0.289
Year 2007	0.064	0.247	0.259	0.795
Year 2008	0.228	0.243	0.939	0.348
Year 2009	0.302	0.233	1.296	0.195
Year 2010	0.574	0.230	2.490	0.013
Year 2011	0.479	0.234	2.046	0.041
Year 2012	−0.126	0.252	−0.498	0.618
Year 2013	0.758	0.231	3.282	0.001
Year 2014	0.671	0.235	2.857	0.004
Year 2015	0.595	0.234	2.546	0.011
Year 2016	0.735	0.232	3.173	0.002
Year 2017	0.598	0.258	2.319	0.020
Year 2018	0.326	0.258	1.264	0.206
Year 2019	0.375	0.260	1.442	0.149
Sin	0.013	0.043	0.296	0.767
Cos	−0.055	0.067	−0.828	0.408
**Smooth terms**		**edf**	**Chi square**	**p-value**
s(X,Y)		1.857	98.662	< 0.001
s(Elevation)		1.005	102.320	< 0.001
s(Slope)		0.942	10.682	< 0.001
s(C110)		1.171	71.180	< 0.001
s(C112)		0.985	20.906	0.000
s(C211)		0.001	0.000	0.795
s(C213)		2.815	521.595	< 0.001
s(C221)		0.768	2.776	0.057
s(C222)		0.783	3.110	0.046
s(C223)		0.000	0.000	0.579
s(C224)		0.001	0.000	0.593
s(C231)		0.943	9.713	0.001
s(C311)		0.000	0.000	0.893
s(C312)		0.001	0.000	0.462
s(C313)		0.831	3.812	0.032
s(C320)		0.000	0.000	0.522
s(C321)		0.759	2.934	0.049
s(C330)		0.988	38.116	< 0.001
s(C410)		0.424	0.775	0.176
s(C511)		1.014	111.684	< 0.001

Grey Heron (*Ardea cinerea*) — Model C-NB-GAM				
**Parametric terms**	**Estimate**	**Standard error**	**z-value**	**p-value**
Intercept	−9.742	1.568	−6.213	< 0.001
Year 1995	0.046	0.221	0.207	0.836
Year 1996	0.269	0.211	1.278	0.201
Year 1999	0.814	0.199	4.091	< 0.001
Year 2000	0.886	0.193	4.593	< 0.001
Year 2001	0.947	0.193	4.910	< 0.001
Year 2002	0.637	0.214	2.973	0.003
Year 2003	0.776	0.213	3.646	< 0.001
Year 2004	0.700	0.236	2.968	0.003
Year 2005	0.855	0.192	4.446	< 0.001
Year 2006	0.762	0.196	3.895	< 0.001
Year 2007	0.452	0.211	2.147	0.032
Year 2008	0.538	0.209	2.577	0.010
Year 2009	0.628	0.202	3.110	0.002
Year 2010	0.590	0.204	2.890	0.004
Year 2011	0.810	0.200	4.050	< 0.001
Year 2012	0.635	0.206	3.085	0.002
Year 2013	0.910	0.199	4.571	< 0.001
Year 2014	0.909	0.201	4.525	< 0.001
Year 2015	0.743	0.202	3.669	< 0.001
Year 2016	0.847	0.202	4.196	< 0.001
Year 2017	0.793	0.217	3.661	< 0.001
Year 2018	0.505	0.221	2.287	0.022
Year 2019	0.621	0.219	2.838	0.005
Sin	0.003	0.031	0.105	0.916
Cos	0.015	0.047	0.326	0.744
**Smooth terms**		**edf**	**Chi square**	**p-value**
s(X,Y)		2.935	76.038	< 0.001
s(Elevation)		2.867	225.553	< 0.001
s(Slope)		2.795	31.002	< 0.001
s(C110)		1.235	93.546	< 0.001
s(C112)		1.021	28.564	< 0.001
s(C211)		0.821	3.683	0.025
s(C213)		2.738	162.063	< 0.001
s(C221)		0.640	1.695	0.102
s(C222)		0.877	6.600	0.006
s(C223)		0.006	0.004	0.393
s(C224)		2.408	28.354	< 0.001
s(C231)		0.019	0.013	0.343
s(C311)		2.739	27.867	< 0.001
s(C312)		0.829	3.816	0.031
s(C313)		0.416	0.676	0.200
s(C320)		0.746	2.794	0.052
s(C321)		0.538	1.386	0.108
s(C330)		0.000	0.000	0.472
s(C410)		0.980	38.584	< 0.001
s(C511)		0.911	9.891	< 0.001

**Black Kite (*Milvus migrans*) — Model C-ZINB-GAM**				
**Binomial component**				
**Parametric terms**	**Estimate**	**Standard error**	**z-value**	**p-value**
Intercept	−2.105	0.027	−77.000	< 0.001
**Smooth terms**		**edf**	**Chi square**	**p-value**
s(Elevation)		1.210	656.520	< 0.001
s(Urb_{2500})		1.040	47.190	< 0.001
s(For_{2500})		2.992	1251.260	< 0.001
**Count component**				
**Parametric terms**	**Estimate**	**Standard error**	**z-value**	**p-value**
Intercept	−2.701	0.394	−6.858	< 0.001
Year 1995	1.539	0.420	3.667	< 0.001
Year 1996	0.360	0.466	0.771	0.441
Year 1999	0.591	0.416	1.422	0.155
Year 2000	0.626	0.409	1.529	0.126
Year 2001	0.281	0.448	0.627	0.530
Year 2002	0.037	0.545	0.067	0.946
Year 2003	0.210	0.493	0.427	0.669
Year 2004	0.860	0.494	1.741	0.082
Year 2005	0.099	0.457	0.216	0.829
Year 2006	0.498	0.421	1.184	0.237
Year 2007	0.028	0.495	0.057	0.954
Year 2008	−0.171	0.519	−0.328	0.743
Year 2009	0.407	0.459	0.887	0.375
Year 2010	0.352	0.461	0.764	0.445
Year 2011	−0.826	0.530	−1.560	0.119
Year 2012	0.172	0.459	0.375	0.708
Year 2013	−0.007	0.482	−0.014	0.989
Year 2014	0.369	0.457	0.807	0.420
Year 2015	−0.050	0.482	−0.104	0.917
Year 2016	0.065	0.495	0.131	0.896
Year 2017	−0.387	0.571	−0.679	0.497
Year 2018	−0.200	0.534	−0.375	0.708
Year 2019	−0.141	0.532	−0.265	0.791
Sin	0.106	0.078	1.368	0.171
Cos	−0.111	0.118	−0.941	0.347
**Smooth terms**		**edf**	**Chi square**	**p-value**
s(X, Y)		1.952	49.583	< 0.001
s(Elevation)		1.284	41.739	< 0.001
s(Slope)		0.642	1.655	0.098
s(C110)		1.049	27.820	< 0.001
s(C112)		0.983	20.901	< 0.001
s(C211)		1.278	42.891	< 0.001
s(C213)		0.952	12.459	< 0.001
s(C221)		0.890	7.124	0.005
s(C222)		0.000	0.000	0.863
s(C223)		0.000	0.000	0.693
s(C224)		0.320	0.446	0.236
s(C231)		0.000	0.000	0.334
s(C311)		1.455	53.163	< 0.001
s(C312)		0.957	11.462	< 0.001
s(C313)		1.021	26.835	< 0.001
s(C320)		0.670	1.828	0.097
s(C321)		0.000	0.000	0.889
s(C330)		0.000	0.000	0.883
s(C410)		0.000	0.000	0.759
s(C511)		0.942	14.729	< 0.001

Common Buzzard (*Buteo buteo*) — Model C-P-GAM				
**Parametric terms**	**Estimate**	**Standard error**	**z-value**	**p-value**
Intercept	−4.698	0.451	−10.414	< 0.001
Year 1995	0.617	0.517	1.194	0.232
Year 1996	0.373	0.527	0.708	0.479
Year 1999	1.368	0.463	2.956	0.003
Year 2000	1.442	0.459	3.141	0.002
Year 2001	1.302	0.469	2.775	0.006
Year 2002	1.676	0.472	3.554	< 0.001
Year 2003	1.152	0.496	2.322	0.020
Year 2004	1.499	0.499	3.007	0.003
Year 2005	1.061	0.476	2.232	0.026
Year 2006	1.292	0.468	2.759	0.006
Year 2007	0.554	0.513	1.080	0.280
Year 2008	1.002	0.496	2.021	0.043
Year 2009	0.996	0.494	2.016	0.044
Year 2010	1.234	0.484	2.553	0.011
Year 2011	0.762	0.494	1.544	0.123
Year 2012	0.971	0.490	1.980	0.048
Year 2013	1.752	0.469	3.737	< 0.001
Year 2014	1.180	0.485	2.435	0.015
Year 2015	1.488	0.476	3.124	0.002
Year 2016	1.492	0.476	3.137	0.002
Year 2017	1.000	0.506	1.975	0.048
Year 2018	0.729	0.521	1.398	0.162
Year 2019	1.167	0.496	2.353	0.019
Sin	−0.013	0.053	−0.250	0.802
Cos	−0.035	0.080	−0.434	0.664
**Smooth terms**		**edf**	**Chi square**	**p-value**
s(X,Y)		2.959	48.721	< 0.001
s(Elevation)		2.575	75.756	< 0.001
s(Slope)		0.675	3.120	0.027
s(C110)		1.027	47.106	< 0.001
s(C112)		0.986	19.469	< 0.001
s(C211)		0.001	0.000	0.737
s(C213)		2.269	17.428	< 0.001
s(C221)		0.012	0.012	0.305
s(C222)		0.000	0.000	0.529
s(C223)		0.000	0.000	0.498
s(C224)		0.003	0.003	0.348
s(C231)		0.892	16.076	< 0.001
s(C311)		2.034	5.661	0.041
s(C312)		0.000	0.000	0.463
s(C313)		0.797	3.220	0.041
s(C320)		2.823	7.859	0.039
s(C321)		0.000	0.000	0.545
s(C330)		0.000	0.000	0.495
s(C410)		0.854	6.808	0.005
s(C511)		1.614	6.214	0.022

Common Kestrel (*Falcon tinnunculus*) — Model C-P-GAM				
**Parametric terms**	**Estimate**	**Standard error**	**z-value**	**p-value**
Intercept	−4.287	0.358	−11.964	< 0.001
Year 1995	−0.286	0.465	−0.614	0.539
Year 1996	−0.514	0.475	−1.083	0.279
Year 1999	0.254	0.414	0.614	0.539
Year 2000	0.630	0.384	1.642	0.101
Year 2001	0.946	0.378	2.502	0.012
Year 2002	0.633	0.414	1.531	0.126
Year 2003	0.868	0.399	2.176	0.030
Year 2004	1.137	0.404	2.814	0.005
Year 2005	1.043	0.373	2.797	0.005
Year 2006	1.278	0.371	3.442	< 0.001
Year 2007	0.970	0.387	2.506	0.012
Year 2008	1.145	0.384	2.985	0.003
Year 2009	0.670	0.395	1.696	0.090
Year 2010	0.992	0.386	2.568	0.010
Year 2011	0.764	0.389	1.966	0.049
Year 2012	0.906	0.386	2.349	0.019
Year 2013	1.022	0.382	2.676	0.007
Year 2014	1.158	0.379	3.052	0.002
Year 2015	1.212	0.378	3.205	0.001
Year 2016	1.361	0.376	3.617	< 0.001
Year 2017	1.383	0.384	3.604	< 0.001
Year 2018	0.475	0.425	1.117	0.264
Year 2019	1.019	0.396	2.575	0.010
Sin	0.030	0.051	0.590	0.555
Cos	−0.080	0.077	−1.041	0.298
**Smooth terms**		**edf**	**Chi square**	**p-value**
s(X,Y)		1.533	9.015	0.002
s(Elevation)		1.828	3.207	0.127
s(Slope)		2.620	22.790	< 0.001
s(C110)		0.656	4.368	0.006
s(C112)		0.437	0.964	0.126
s(C211)		0.967	26.608	< 0.001
s(C213)		0.000	0.000	0.533
s(C221)		0.807	3.283	0.040
s(C222)		0.000	0.000	0.688
s(C223)		0.004	0.000	0.959
s(C224)		0.000	0.000	0.987
s(C231)		1.179	48.353	< 0.001
s(C311)		1.985	13.373	< 0.001
s(C312)		0.940	14.292	< 0.001
s(C313)		0.854	9.731	< 0.001
s(C320)		0.391	0.652	0.186
s(C321)		0.921	14.734	0.000
s(C330)		0.911	11.302	< 0.001
s(C410)		0.001	0.000	0.552
s(C511)		0.894	4.980	0.017

**Common Quail (*Coturnix coturnix*) — Model C-ZIP-GAM**				
**Binomial component**				
**Parametric terms**	**Estimate**	**Standard error**	**z-value**	**p-value**
Intercept	−1.455	0.023	−62.340	< 0.001
**Smooth terms**		**edf**	**Chi square**	**p-value**
s(Elevation)		2.990	480.600	< 0.001
s(Urb_{2500})		1.106	179.800	< 0.001
s(For_{2500})		1.907	502.200	< 0.001
**Count component**				
**Parametric terms**	**Estimate**	**Standard error**	**z-value**	**p-value**
Intercept	−3.430	0.334	−10.270	< 0.001
Year 1995	0.562	0.347	1.621	0.105
Year 1996	−1.285	0.509	−2.524	0.012
Year 1999	0.208	0.380	0.547	0.585
Year 2000	0.660	0.338	1.953	0.051
Year 2001	0.762	0.334	2.281	0.023
Year 2002	0.168	0.395	0.425	0.671
Year 2003	0.237	0.400	0.592	0.554
Year 2004	0.113	0.440	0.256	0.798
Year 2005	1.370	0.316	4.339	< 0.001
Year 2006	0.741	0.334	2.222	0.026
Year 2007	0.115	0.366	0.313	0.754
Year 2008	0.269	0.381	0.706	0.480
Year 2009	0.157	0.375	0.418	0.676
Year 2010	0.797	0.342	2.328	0.020
Year 2011	0.094	0.371	0.253	0.800
Year 2012	−0.035	0.378	−0.093	0.926
Year 2013	−0.250	0.394	−0.636	0.525
Year 2014	−0.486	0.439	−1.107	0.268
Year 2015	−0.281	0.412	−0.684	0.494
Year 2016	−0.490	0.429	−1.143	0.253
Year 2017	−0.573	0.485	−1.182	0.237
Year 2018	−1.049	0.585	−1.793	0.073
Year 2019	0.240	0.394	0.609	0.543
Sin	0.013	0.064	0.202	0.840
Cos	−0.039	0.095	−0.411	0.681
**Smooth terms**		**edf**	**Chi square**	**p-value**
s(X, Y)		2.907	25.309	< 0.001
s(Elevation)		1.197	25.483	< 0.001
s(Slope)		1.035	7.790	0.002
s(C110)		0.808	6.106	0.004
s(C112)		0.869	7.004	0.004
s(C211)		1.090	42.005	< 0.001
s(C213)		0.861	9.760	< 0.001
s(C221)		2.506	21.686	< 0.001
s(C222)		0.929	12.181	< 0.001
s(C223)		0.000	0.000	0.534
s(C224)		0.001	0.000	0.724
s(C231)		1.665	63.280	< 0.001
s(C311)		2.629	17.432	< 0.001
s(C312)		1.809	8.574	0.007
s(C313)		0.877	5.583	0.011
s(C320)		0.000	0.000	0.706
s(C321)		0.193	0.235	0.261
s(C330)		2.907	25.309	< 0.001
s(C410)		1.197	25.483	< 0.001
s(C511)		1.035	7.790	0.002

Common Pheasant (*Phasianus colchicus*) — Model C-NB-GAM				
**Parametric terms**	**Estimate**	**Standard error**	**z-value**	**p-value**
Intercept	−4.305	0.222	−19.389	< 0.001
Year 1995	−1.141	0.272	−4.193	< 0.001
Year 1996	−0.584	0.241	−2.426	0.015
Year 1999	−0.046	0.200	−0.230	0.818
Year 2000	0.300	0.194	1.546	0.122
Year 2001	0.087	0.202	0.430	0.667
Year 2002	−0.048	0.232	−0.205	0.837
Year 2003	0.314	0.216	1.452	0.147
Year 2004	−0.290	0.294	−0.986	0.324
Year 2005	−0.012	0.201	−0.060	0.952
Year 2006	0.337	0.194	1.735	0.083
Year 2007	0.748	0.198	3.775	< 0.001
Year 2008	0.622	0.205	3.040	0.002
Year 2009	0.942	0.194	4.858	< 0.001
Year 2010	0.927	0.195	4.748	< 0.001
Year 2011	0.806	0.193	4.171	< 0.001
Year 2012	0.872	0.195	4.466	< 0.001
Year 2013	0.979	0.191	5.125	< 0.001
Year 2014	0.782	0.197	3.960	< 0.001
Year 2015	0.926	0.196	4.733	< 0.001
Year 2016	0.874	0.196	4.452	< 0.001
Year 2017	0.750	0.208	3.610	< 0.001
Year 2018	0.772	0.208	3.714	< 0.001
Year 2019	0.604	0.213	2.839	0.005
Sin	−0.001	0.034	−0.031	0.975
Cos	0.039	0.051	0.767	0.443
**Smooth terms**		**edf**	**Chi square**	**p-value**
s(X,Y)		1.981	101.266	< 0.001
s(Elevation)		1.395	93.493	< 0.001
s(Slope)		1.977	22.295	< 0.001
s(C110)		1.377	177.353	< 0.001
s(C112)		1.104	74.221	< 0.001
s(C211)		0.001	0.000	0.752
s(C213)		0.949	13.721	< 0.001
s(C221)		0.225	0.293	0.249
s(C222)		0.001	0.000	0.789
s(C223)		0.001	0.000	0.713
s(C224)		2.213	123.386	< 0.001
s(C231)		0.731	2.575	0.058
s(C311)		2.640	130.881	< 0.001
s(C312)		0.143	0.182	0.259
s(C313)		0.001	0.000	0.936
s(C320)		0.448	0.856	0.166
s(C321)		0.002	0.001	0.403
s(C330)		0.005	0.005	0.329
s(C410)		0.001	0.000	0.759
s(C511)		0.741	2.803	0.050

Common Moorhen (*Gallinula chloropus*) — Model C-NB-GAM				
**Parametric terms**	**Estimate**	**Standard error**	**z-value**	**p-value**
Intercept	−5.900	0.969	−6.086	< 0.001
Year 1995	−0.097	0.228	−0.426	0.670
Year 1996	0.248	0.221	1.125	0.261
Year 1999	−0.317	0.233	−1.363	0.173
Year 2000	0.461	0.206	2.235	0.025
Year 2001	0.416	0.207	2.010	0.044
Year 2002	0.026	0.243	0.109	0.913
Year 2003	0.140	0.250	0.560	0.576
Year 2004	−0.089	0.305	−0.291	0.771
Year 2005	0.272	0.207	1.312	0.189
Year 2006	0.158	0.215	0.737	0.461
Year 2007	−0.306	0.248	−1.236	0.217
Year 2008	0.211	0.229	0.922	0.357
Year 2009	0.187	0.220	0.851	0.395
Year 2010	0.436	0.215	2.025	0.043
Year 2011	−0.281	0.244	−1.154	0.248
Year 2012	0.050	0.238	0.210	0.834
Year 2013	0.228	0.226	1.012	0.312
Year 2014	0.047	0.239	0.195	0.846
Year 2015	0.223	0.224	0.995	0.320
Year 2016	0.227	0.228	0.996	0.320
Year 2017	0.380	0.243	1.559	0.119
Year 2018	0.132	0.245	0.540	0.589
Year 2019	0.081	0.257	0.314	0.753
Sin	0.018	0.042	0.438	0.661
Cos	0.049	0.063	0.769	0.442
**Smooth terms**		**edf**	**Chi square**	**p-value**
s(X,Y)		1.640	23.987	< 0.001
s(Elevation)		1.142	62.351	< 0.001
s(Slope)		0.876	5.000	0.013
s(C110)		1.184	79.117	< 0.001
s(C112)		0.959	13.000	< 0.001
s(C211)		0.088	0.098	0.276
s(C213)		1.316	253.632	< 0.001
s(C221)		0.006	0.004	0.370
s(C222)		0.579	1.204	0.149
s(C223)		0.632	0.144	0.633
s(C224)		0.669	1.944	0.085
s(C231)		0.001	0.000	0.772
s(C311)		2.484	26.561	< 0.001
s(C312)		0.000	0.000	0.786
s(C313)		0.839	3.918	0.030
s(C320)		0.666	1.842	0.095
s(C321)		0.000	0.000	0.634
s(C330)		0.857	5.362	0.012
s(C410)		2.848	371.328	< 0.001
s(C511)		2.891	107.743	< 0.001

Northern Lapwing (*Vanellus vanellus*) — Model C-NB-GAM				
**Parametric terms**	**Estimate**	**Standard error**	**z-value**	**p-value**
Intercept	−15.198	2.237	−6.794	< 0.001
Year 1996	−2.095	0.863	−2.428	0.015
Year 1999	−0.003	0.599	−0.004	0.997
Year 2000	0.074	0.513	0.145	0.885
Year 2001	0.651	0.492	1.322	0.186
Year 2002	0.494	0.547	0.903	0.367
Year 2003	1.027	0.582	1.765	0.078
Year 2004	1.425	0.572	2.489	0.013
Year 2005	1.036	0.482	2.150	0.032
Year 2006	0.443	0.524	0.846	0.398
Year 2007	0.922	0.514	1.793	0.073
Year 2008	1.345	0.500	2.692	0.007
Year 2009	1.105	0.488	2.267	0.023
Year 2010	1.510	0.481	3.135	0.002
Year 2011	1.102	0.502	2.194	0.028
Year 2012	0.923	0.511	1.808	0.071
Year 2013	1.573	0.499	3.154	0.002
Year 2014	2.585	0.481	5.375	< 0.001
Year 2015	1.679	0.490	3.429	< 0.001
Year 2016	1.962	0.482	4.069	< 0.001
Year 2017	2.134	0.525	4.063	< 0.001
Year 2018	1.338	0.534	2.506	0.012
Year 2019	1.780	0.526	3.388	< 0.001
Sin	0.343	0.097	3.520	< 0.001
Cos	−0.338	0.155	−2.176	0.030
**Smooth terms**		**edf**	**Chi square**	**p-value**
s(X,Y)		1.924	32.105	< 0.001
s(Elevation)		0.000	0.000	0.830
s(Slope)		0.952	17.976	< 0.001
s(C110)		0.911	7.397	0.004
s(C112)		0.000	0.000	0.481
s(C211)		1.773	85.008	< 0.001
s(C213)		2.475	257.576	< 0.001
s(C221)		0.000	0.000	0.966
s(C222)		0.000	0.000	0.651
s(C223)		0.000	0.000	0.816
s(C224)		0.946	14.829	< 0.001
s(C231)		0.000	0.000	0.827
s(C311)		0.002	0.002	0.327
s(C312)		0.002	0.001	0.455
s(C313)		0.673	1.615	0.121
s(C320)		0.586	1.094	0.172
s(C321)		0.604	2.243	0.054
s(C330)		0.778	2.644	0.065
s(C410)		0.955	20.504	< 0.001
s(C511)		0.974	29.611	< 0.001

Feral Pigeon (*Columba livia domestica*) — Model C-ZINB-GAM				
**Binomial component**				
**Parametric terms**	**Estimate**	**Standard error**	**z-value**	**p-value**
Intercept	0.502	0.027	18.950	< 0.001
**Smooth terms**		**edf**	**Chi square**	**p-value**
s(Elevation)		2.988	757.300	< 0.001
s(Urb_{2500})		1.162	1017.600	< 0.001
s(For_{2500})		2.723	464.300	< 0.001
**Count component**				
**Parametric terms**	**Estimate**	**Standard error**	**z-value**	**p-value**
Intercept	−2.205	0.174	−12.654	< 0.001
Year 1995	0.099	0.179	0.553	0.580
Year 1996	−0.297	0.178	−1.666	0.096
Year 1999	0.107	0.180	0.593	0.553
Year 2000	0.897	0.162	5.526	< 0.001
Year 2001	0.946	0.162	5.832	< 0.001
Year 2002	1.316	0.177	7.456	< 0.001
Year 2003	0.373	0.184	2.023	0.043
Year 2004	1.519	0.190	7.995	< 0.001
Year 2005	0.942	0.161	5.843	< 0.001
Year 2006	1.052	0.162	6.513	< 0.001
Year 2007	0.039	0.177	0.220	0.826
Year 2008	−0.039	0.178	−0.222	0.824
Year 2009	0.024	0.173	0.136	0.892
Year 2010	0.241	0.171	1.409	0.159
Year 2011	0.523	0.167	3.125	0.002
Year 2012	0.206	0.174	1.186	0.235
Year 2013	0.319	0.170	1.872	0.061
Year 2014	0.453	0.166	2.738	0.006
Year 2015	0.438	0.170	2.580	0.010
Year 2016	0.390	0.171	2.272	0.023
Year 2017	0.431	0.185	2.335	0.020
Year 2018	0.478	0.184	2.603	0.009
Year 2019	0.502	0.183	2.739	0.006
Sin	0.052	0.031	1.707	0.088
Cos	−0.060	0.047	−1.290	0.197
**Smooth terms**		**edf**	**Chi square**	**p-value**
s(X, Y)		1.996	323.828	< 0.001
s(Elevation)		1.138	11.942	< 0.001
s(Slope)		1.035	15.402	< 0.001
s(C110)		0.652	1.716	0.094
s(C112)		2.600	33.562	< 0.001
s(C211)		0.924	6.811	0.003
s(C213)		0.034	0.022	0.396
s(C221)		0.950	17.385	< 0.001
s(C222)		0.004	0.003	0.418
s(C223)		0.850	5.136	0.014
s(C224)		0.999	47.638	< 0.001
s(C231)		0.001	0.000	0.356
s(C311)		1.669	254.304	< 0.001
s(C312)		1.003	22.758	< 0.001
s(C313)		1.063	47.353	< 0.001
s(C320)		0.906	8.368	0.002
s(C321)		0.797	3.127	0.044
s(C330)		0.001	0.000	0.784
s(C410)		0.970	28.490	< 0.001
s(C511)		0.949	17.730	< 0.001

Common Wood Pigeon (*Columba palumbus*) — Model C-ZIP-GAM				
**Binomial component**				
**Parametric terms**	**Estimate**	**Standard error**	**z-value**	**p-value**
Intercept	0.575	0.020	28.700	< 0.001
**Smooth terms**		**edf**	**Chi square**	**p-value**
s(Elevation)		1.170	737.300	< 0.001
s(Urb_{2500})		2.981	647.900	< 0.001
s(For_{2500})		2.990	480.700	< 0.001
**Count component**				
**Parametric terms**	**Estimate**	**Standard error**	**z-value**	**p-value**
Intercept	−2.529	0.174	−14.505	< 0.001
Year 1995	−1.087	0.235	−4.626	< 0.001
Year 1996	−0.308	0.179	−1.720	0.085
Year 1999	−0.019	0.169	−0.110	0.912
Year 2000	−0.462	0.185	−2.494	0.013
Year 2001	−0.634	0.225	−2.818	0.005
Year 2002	−0.572	0.225	−2.540	0.011
Year 2003	−0.644	0.264	−2.440	0.015
Year 2004	−0.374	0.182	−2.057	0.040
Year 2005	0.327	0.163	2.007	0.045
Year 2006	0.072	0.180	0.402	0.688
Year 2007	0.272	0.175	1.554	0.120
Year 2008	0.420	0.171	2.461	0.014
Year 2009	0.684	0.165	4.139	< 0.001
Year 2010	0.963	0.159	6.054	< 0.001
Year 2011	0.832	0.165	5.057	< 0.001
Year 2012	1.007	0.161	6.269	< 0.001
Year 2013	1.086	0.157	6.897	< 0.001
Year 2014	1.058	0.160	6.602	< 0.001
Year 2015	1.299	0.158	8.233	< 0.001
Year 2016	1.177	0.163	7.216	< 0.001
Year 2017	1.334	0.161	8.274	< 0.001
Year 2018	1.333	0.161	8.276	< 0.001
Year 2019	0.006	0.027	0.219	0.827
Sin	−0.199	0.043	−4.605	< 0.001
Cos	−2.529	0.174	−14.505	< 0.001
**Smooth terms**		**edf**	**Chi square**	**p-value**
s(X, Y)		1.950	72.541	< 0.001
s(Elevation)		2.587	39.005	< 0.001
s(Slope)		1.306	54.017	< 0.001
s(C110)		0.001	0.000	0.753
s(C112)		0.988	21.845	< 0.001
s(C211)		2.090	12.796	< 0.001
s(C213)		1.009	34.233	< 0.001
s(C221)		0.888	7.562	0.003
s(C222)		0.002	0.000	0.828
s(C223)		0.666	1.789	0.101
s(C224)		0.386	0.639	0.197
s(C231)		0.001	0.000	0.729
s(C311)		2.702	82.454	< 0.001
s(C312)		0.043	0.043	0.316
s(C313)		0.856	5.323	0.012
s(C320)		0.173	0.212	0.266
s(C321)		0.847	4.372	0.022
s(C330)		0.804	3.792	0.029
s(C410)		0.315	0.438	0.238
s(C511)		0.002	0.000	0.891

**Eurasian Collared Dove (*Streptopelia decaocto*) — Model C-ZINB-GAM**				
**Binomial component**				
**Parametric terms**	**Estimate**	**Standard error**	**z-value**	**p-value**
Intercept	0.667	0.020	33.510	< 0.001
**Smooth terms**		**edf**	**Chi square**	**p-value**
s(Elevation)		1.206	857.000	< 0.001
s(Urb_{2500})		2.835	469.300	< 0.001
s(For_{2500})		2.522	1479.500	< 0.001
**Count component**				
**Parametric terms**	**Estimate**	**Standard error**	**z-value**	**p-value**
Intercept	−3.987	0.609	−6.547	< 0.001
Year 1995	−0.267	0.155	−1.723	0.085
Year 1996	−0.076	0.137	−0.553	0.581
Year 1999	0.441	0.137	3.205	0.001
Year 2000	0.816	0.123	6.625	< 0.001
Year 2001	0.793	0.124	6.409	< 0.001
Year 2002	0.859	0.130	6.612	< 0.001
Year 2003	0.789	0.130	6.049	< 0.001
Year 2004	0.782	0.141	5.558	< 0.001
Year 2005	0.999	0.120	8.328	< 0.001
Year 2006	0.920	0.121	7.572	< 0.001
Year 2007	0.784	0.126	6.243	< 0.001
Year 2008	0.723	0.126	5.739	< 0.001
Year 2009	0.932	0.123	7.592	< 0.001
Year 2010	0.888	0.124	7.139	< 0.001
Year 2011	0.754	0.123	6.146	< 0.001
Year 2012	0.951	0.123	7.710	< 0.001
Year 2013	0.792	0.123	6.462	< 0.001
Year 2014	0.721	0.122	5.893	< 0.001
Year 2015	0.666	0.124	5.391	< 0.001
Year 2016	0.810	0.123	6.564	< 0.001
Year 2017	0.895	0.128	6.977	< 0.001
Year 2018	0.938	0.127	7.368	< 0.001
Year 2019	0.977	0.127	7.712	< 0.001
Sin	0.018	0.018	0.990	0.322
Cos	−0.041	0.027	−1.521	0.128
**Smooth terms**		**edf**	**Chi square**	**p-value**
s(X, Y)		2.967	289.032	< 0.001
s(Elevation)		2.906	20.032	< 0.001
s(Slope)		2.010	66.178	< 0.001
s(C110)		2.590	1069.957	< 0.001
s(C112)		2.942	1047.404	< 0.001
s(C211)		0.005	0.001	0.661
s(C213)		1.831	62.062	< 0.001
s(C221)		0.269	0.379	0.233
s(C222)		2.739	5.245	0.125
s(C223)		0.000	0.000	0.552
s(C224)		0.956	24.219	< 0.001
s(C231)		0.886	8.591	0.002
s(C311)		2.159	96.835	< 0.001
s(C312)		0.001	0.000	0.543
s(C313)		0.932	11.455	< 0.001
s(C320)		1.291	3.072	0.101
s(C321)		0.229	0.315	0.241
s(C330)		0.944	18.358	< 0.001
s(C410)		2.109	25.165	< 0.001
s(C511)		0.958	23.479	< 0.001

European Turtle Dove (*Streptopelia turtur*) — Model C-ZIP-GAM				
**Binomial component**				
**Parametric terms**	**Estimate**	**Standard error**	**z-value**	**p-value**
Intercept	−0.469	0.019	−24.720	< 0.001
**Smooth terms**		**edf**	**Chi square**	**p-value**
s(Elevation)		3.000	2447.200	< 0.001
s(Urb_{2500})		1.157	288.500	< 0.001
s(For_{2500})		2.999	1788.800	< 0.001
**Count component**				
**Parametric terms**	**Estimate**	**Standard error**	**z-value**	**p-value**
Intercept	−2.546	0.209	−12.160	< 0.001
Year 1995	0.037	0.152	0.244	0.807
Year 1996	−1.128	0.211	−5.349	< 0.001
Year 1999	0.071	0.145	0.488	0.626
Year 2000	0.147	0.141	1.044	0.297
Year 2001	−0.386	0.162	−2.381	0.017
Year 2002	−0.075	0.162	−0.464	0.643
Year 2003	−0.687	0.202	−3.398	< 0.001
Year 2004	−0.371	0.243	−1.528	0.126
Year 2005	−0.355	0.154	−2.306	0.021
Year 2006	−0.332	0.153	−2.171	0.030
Year 2007	−0.010	0.151	−0.065	0.948
Year 2008	0.023	0.156	0.148	0.882
Year 2009	0.252	0.148	1.704	0.088
Year 2010	0.248	0.146	1.701	0.089
Year 2011	−0.302	0.155	−1.953	0.051
Year 2012	0.072	0.153	0.469	0.639
Year 2013	−0.149	0.157	−0.948	0.343
Year 2014	0.034	0.152	0.225	0.822
Year 2015	−0.204	0.162	−1.263	0.206
Year 2016	−0.103	0.157	−0.659	0.510
Year 2017	−0.107	0.168	−0.633	0.527
Year 2018	−0.246	0.172	−1.429	0.153
Year 2019	−0.323	0.178	−1.812	0.070
Sin	−0.047	0.031	−1.510	0.131
Cos	−0.080	0.050	−1.605	0.108
**Smooth terms**		**edf**	**Chi square**	**p-value**
s(X, Y)		2.996	458.094	< 0.001
s(Elevation)		2.654	39.242	< 0.001
s(Slope)		2.225	46.249	< 0.001
s(C110)		1.043	80.131	< 0.001
s(C112)		2.531	71.924	< 0.001
s(C211)		1.971	7.464	0.016
s(C213)		1.946	8.695	0.008
s(C221)		2.223	5.833	0.055
s(C222)		0.001	0.001	0.457
s(C223)		0.615	2.097	0.064
s(C224)		0.000	0.000	0.742
s(C231)		0.002	0.000	0.924
s(C311)		2.659	77.273	< 0.001
s(C312)		0.763	3.165	0.041
s(C313)		0.002	0.000	0.877
s(C320)		1.244	27.083	0.000
s(C321)		0.002	0.002	0.423
s(C330)		0.001	0.000	0.682
s(C410)		0.847	7.111	0.004
s(C511)		1.704	2.530	0.218

Common Cuckoo (*Cuculus canorus*) — Model C-ZIP-GAM				
**Binomial component**				
**Parametric terms**	**Estimate**	**Standard error**	**z-value**	**p-value**
Intercept	4.987	0.317	15.740	< 0.001
**Smooth terms**		**edf**	**Chi square**	**p-value**
s(Elevation)		2.946	1076.000	< 0.001
s(Urb_{2500})		2.984	1132.000	< 0.001
s(For_{2500})		2.991	1636.000	< 0.001
**Count component**				
**Parametric terms**	**Estimate**	**Standard error**	**z-value**	**p-value**
Intercept	−0.789	0.075	−10.538	< 0.001
Year 1995	−0.086	0.097	−0.887	0.375
Year 1996	−0.650	0.110	−5.937	< 0.001
Year 1999	−0.198	0.086	−2.308	0.021
Year 2000	−0.344	0.085	−4.042	< 0.001
Year 2001	−0.361	0.094	−3.838	< 0.001
Year 2002	−0.274	0.099	−2.781	0.005
Year 2003	−0.148	0.101	−1.473	0.141
Year 2004	−0.100	0.114	−0.875	0.382
Year 2005	0.018	0.087	0.211	0.833
Year 2006	−0.151	0.090	−1.692	0.091
Year 2007	−0.139	0.094	−1.481	0.139
Year 2008	−0.383	0.100	−3.812	< 0.001
Year 2009	−0.269	0.098	−2.757	0.006
Year 2010	−0.050	0.093	−0.537	0.591
Year 2011	−0.006	0.088	−0.071	0.943
Year 2012	−0.296	0.096	−3.066	0.002
Year 2013	−0.195	0.094	−2.081	0.037
Year 2014	0.096	0.090	1.070	0.285
Year 2015	0.009	0.092	0.097	0.922
Year 2016	−0.036	0.092	−0.390	0.696
Year 2017	−0.199	0.101	−1.968	0.049
Year 2018	−0.262	0.102	−2.573	0.010
Year 2019	−0.337	0.104	−3.233	0.001
Sin	−0.008	0.018	−0.463	0.643
Cos	0.015	0.027	0.549	0.583
**Smooth terms**		**edf**	**Chi square**	**p-value**
s(X, Y)		2.971	335.601	< 0.001
s(Elevation)		2.643	32.400	< 0.001
s(Slope)		2.795	77.192	< 0.001
s(C110)		2.941	90.119	< 0.001
s(C112)		2.885	16.403	0.001
s(C211)		0.001	0.000	0.571
s(C213)		2.365	31.445	< 0.001
s(C221)		0.954	19.392	< 0.001
s(C222)		0.496	1.006	0.154
s(C223)		0.000	0.000	0.753
s(C224)		2.236	99.193	< 0.001
s(C231)		1.013	40.345	< 0.001
s(C311)		2.887	119.877	< 0.001
s(C312)		0.718	4.209	0.014
s(C313)		1.812	27.925	< 0.001
s(C320)		0.961	12.854	0.000
s(C321)		2.847	50.306	0.000
s(C330)		2.255	25.863	< 0.001
s(C410)		2.924	209.291	< 0.001
s(C511)		0.940	14.920	< 0.001

Common Swift (*Apus apus*) — Model C-ZINB-GAM				
**Binomial component**				
**Parametric terms**	**Estimate**	**Standard error**	**z-value**	**p-value**
Intercept	0.831	0.022	37.700	< 0.001
**Smooth terms**		**edf**	**Chi square**	**p-value**
s(Elevation)		1.170	665.070	< 0.001
s(Urb_{2500})		1.112	1027.400	< 0.001
s(For_{2500})		2.833	60.010	< 0.001
**Count component**				
**Parametric terms**	**Estimate**	**Standard error**	**z-value**	**p-value**
Intercept	0.922	0.127	7.258	< 0.001
Year 1995	−0.780	0.159	−4.908	< 0.001
Year 1996	−0.669	0.156	−4.284	< 0.001
Year 1999	−0.278	0.147	−1.887	0.059
Year 2000	−0.601	0.142	−4.237	< 0.001
Year 2001	−0.594	0.148	−4.005	< 0.001
Year 2002	−0.458	0.162	−2.834	0.005
Year 2003	−0.217	0.163	−1.334	0.182
Year 2004	−0.335	0.178	−1.884	0.060
Year 2005	−0.485	0.146	−3.319	< 0.001
Year 2006	−0.601	0.144	−4.167	< 0.001
Year 2007	−1.300	0.162	−8.042	< 0.001
Year 2008	−0.949	0.159	−5.971	< 0.001
Year 2009	−1.072	0.157	−6.836	< 0.001
Year 2010	−0.561	0.154	−3.644	< 0.001
Year 2011	−0.793	0.150	−5.298	< 0.001
Year 2012	−0.834	0.156	−5.341	< 0.001
Year 2013	−0.886	0.155	−5.710	< 0.001
Year 2014	−0.848	0.150	−5.646	< 0.001
Year 2015	−0.853	0.155	−5.520	< 0.001
Year 2016	−1.361	0.159	−8.573	< 0.001
Year 2017	−1.510	0.175	−8.652	< 0.001
Year 2018	−1.113	0.171	−6.503	< 0.001
Year 2019	−0.453	0.167	−2.716	0.007
Sin	0.065	0.030	2.214	0.027
Cos	−0.127	0.044	−2.889	0.004
**Smooth terms**		**edf**	**Chi square**	**p-value**
s(X, Y)		1.906	52.724	< 0.001
s(Elevation)		2.895	50.998	< 0.001
s(Slope)		0.411	0.680	0.194
s(C110)		1.022	16.871	0.000
s(C112)		0.876	6.340	0.005
s(C211)		1.979	29.953	0.000
s(C213)		0.903	7.950	0.002
s(C221)		0.956	19.609	0.000
s(C222)		0.800	4.087	0.023
s(C223)		0.851	5.823	0.009
s(C224)		0.905	9.191	0.001
s(C231)		0.619	1.562	0.094
s(C311)		2.679	160.801	0.000
s(C312)		1.237	108.943	0.000
s(C313)		1.269	147.769	0.000
s(C320)		0.956	16.345	0.000
s(C321)		0.001	0.000	0.907
s(C330)		0.003	0.002	0.411
s(C410)		0.002	0.000	0.755
s(C511)		0.002	0.000	0.811

European Bee-eater (*Merops apiaster*) — Model C-NB-GAM				
**Parametric terms**	**Estimate**	**Standard error**	**z-value**	**p-value**
Intercept	−8.994	0.936	−9.608	< 0.001
Year 1995	0.906	0.917	0.989	0.323
Year 1996	−0.162	1.035	−0.157	0.875
Year 1999	1.951	0.857	2.276	0.023
Year 2000	1.608	0.869	1.851	0.064
Year 2001	1.745	0.863	2.022	0.043
Year 2002	1.867	0.886	2.108	0.035
Year 2003	2.746	0.864	3.178	0.001
Year 2004	1.430	0.996	1.437	0.151
Year 2005	2.495	0.843	2.959	0.003
Year 2006	0.904	0.895	1.009	0.313
Year 2007	2.532	0.860	2.944	0.003
Year 2008	2.023	0.886	2.283	0.022
Year 2009	1.956	0.877	2.229	0.026
Year 2010	3.261	0.850	3.838	< 0.001
Year 2011	2.418	0.857	2.821	0.005
Year 2012	2.152	0.870	2.474	0.013
Year 2013	2.944	0.849	3.467	< 0.001
Year 2014	2.853	0.855	3.338	< 0.001
Year 2015	2.744	0.860	3.192	0.001
Year 2016	2.826	0.855	3.306	< 0.001
Year 2017	2.987	0.867	3.444	< 0.001
Year 2018	3.262	0.865	3.771	< 0.001
Year 2019	3.177	0.865	3.675	< 0.001
Sin	−0.208	0.093	−2.246	0.025
Cos	0.140	0.138	1.014	0.311
**Smooth terms**		**edf**	**Chi square**	**p-value**
s(X,Y)		1.999	125.125	< 0.001
s(Elevation)		1.161	48.712	< 0.001
s(Slope)		2.311	22.706	< 0.001
s(C110)		2.144	18.500	< 0.001
s(C112)		0.956	13.830	< 0.001
s(C211)		0.003	0.002	0.394
s(C213)		1.017	37.487	< 0.001
s(C221)		0.855	5.454	0.011
s(C222)		0.740	2.881	0.048
s(C223)		0.000	0.000	0.558
s(C224)		0.158	0.169	0.301
s(C231)		0.046	0.042	0.336
s(C311)		2.604	22.156	< 0.001
s(C312)		0.001	0.001	0.470
s(C313)		0.000	0.000	0.580
s(C320)		0.000	0.000	0.490
s(C321)		0.000	0.000	0.585
s(C330)		0.939	12.666	< 0.001
s(C410)		0.000	0.000	0.373
s(C511)		0.931	12.223	< 0.001

Eurasian Wryneck (*Jynx torquilla*) — Model C-ZIP-GAM				
**Binomial component**				
**Parametric terms**	**Estimate**	**Standard error**	**z-value**	**p-value**
Intercept	−2.106	0.035	−60.520	< 0.001
**Smooth terms**		**edf**	**Chi square**	**p-value**
s(Elevation)		2.994	818.020	< 0.001
s(Urb_{2500})		2.979	72.450	< 0.001
s(For_{2500})		2.974	332.040	< 0.001
**Count component**				
**Parametric terms**	**Estimate**	**Standard error**	**z-value**	**p-value**
Intercept	−2.928	0.723	−4.052	< 0.001
Year 1995	0.072	0.298	0.242	0.809
Year 1996	−0.347	0.344	−1.008	0.313
Year 1999	0.310	0.286	1.081	0.280
Year 2000	0.146	0.267	0.545	0.586
Year 2001	−0.044	0.299	−0.147	0.883
Year 2002	0.208	0.332	0.626	0.532
Year 2003	0.271	0.349	0.776	0.438
Year 2004	0.120	0.411	0.293	0.770
Year 2005	−0.309	0.302	−1.023	0.306
Year 2006	−0.898	0.350	−2.561	0.010
Year 2007	0.298	0.285	1.045	0.296
Year 2008	−0.060	0.302	−0.197	0.844
Year 2009	−0.384	0.349	−1.100	0.271
Year 2010	−0.414	0.319	−1.299	0.194
Year 2011	−0.837	0.349	−2.399	0.016
Year 2012	−0.691	0.364	−1.898	0.058
Year 2013	−0.392	0.339	−1.159	0.246
Year 2014	−0.605	0.357	−1.693	0.090
Year 2015	−1.011	0.395	−2.561	0.010
Year 2016	−1.660	0.505	−3.287	0.001
Year 2017	−1.167	0.471	−2.477	0.013
Year 2018	−1.562	0.552	−2.828	0.005
Year 2019	−1.681	0.552	−3.043	0.002
Sin	−0.145	0.071	−2.029	0.042
Cos	−0.092	0.108	−0.855	0.393
**Smooth terms**		**edf**	**Chi square**	**p-value**
s(X, Y)		2.956	111.980	< 0.001
s(Elevation)		0.000	0.000	0.536
S(Slope)		0.928	7.061	0.004
s(C110)		0.049	0.055	0.284
s(C112)		2.681	4.330	0.182
s(C211)		0.000	0.000	0.746
s(C213)		0.734	2.549	0.062
s(C221)		2.680	98.820	< 0.001
s(C222)		0.842	5.123	0.013
s(C223)		0.889	8.045	0.003
s(C224)		2.567	15.429	< 0.001
s(C231)		1.527	40.971	< 0.001
s(C311)		2.446	51.158	< 0.001
s(C312)		0.330	0.499	0.217
s(C313)		0.000	0.000	0.803
s(C320)		0.674	2.167	0.072
s(C321)		1.787	6.650	0.024
s(C330)		0.750	3.748	0.025
s(C410)		0.001	0.001	0.411
s(C511)		0.906	19.469	< 0.001

European Green Woodpecker (*Picus viridis*) — Model C-P-GAM				
**Parametric terms**	**Estimate**	**Standard error**	**z-value**	**p-value**
Intercept	−4.031	0.293	−13.736	< 0.001
Year 1995	−0.622	0.429	−1.450	0.147
Year 1996	−0.097	0.378	−0.258	0.797
Year 1999	0.349	0.318	1.097	0.273
Year 2000	0.943	0.304	3.100	0.002
Year 2001	0.903	0.316	2.859	0.004
Year 2002	0.673	0.332	2.028	0.043
Year 2003	0.822	0.338	2.429	0.015
Year 2004	−0.015	0.442	−0.034	0.973
Year 2005	0.583	0.323	1.807	0.071
Year 2006	0.804	0.312	2.575	0.010
Year 2007	0.785	0.326	2.410	0.016
Year 2008	0.738	0.335	2.202	0.028
Year 2009	0.571	0.339	1.684	0.092
Year 2010	0.662	0.332	1.995	0.046
Year 2011	1.253	0.308	4.062	< 0.001
Year 2012	1.117	0.318	3.517	< 0.001
Year 2013	1.168	0.315	3.715	< 0.001
Year 2014	1.109	0.316	3.510	< 0.001
Year 2015	1.324	0.313	4.227	< 0.001
Year 2016	1.161	0.316	3.671	< 0.001
Year 2017	1.201	0.323	3.721	< 0.001
Year 2018	0.991	0.330	3.005	0.003
Year 2019	1.255	0.321	3.902	0.000
Sin	−0.018	0.043	−0.418	0.676
Cos	0.057	0.066	0.858	0.391
**Smooth terms**		**edf**	**Chi square**	**p-value**
s(X,Y)		2.973	137.189	< 0.001
s(Elevation)		2.942	72.707	< 0.001
s(Slope)		2.840	16.272	< 0.001
s(C110)		0.931	7.285	0.003
s(C112)		0.846	4.172	0.022
s(C211)		2.260	15.935	< 0.001
s(C213)		0.909	8.216	0.002
s(C221)		1.485	24.963	< 0.001
s(C222)		0.000	0.000	0.665
s(C223)		0.533	0.860	0.203
s(C224)		2.632	29.737	< 0.001
s(C231)		2.471	14.594	< 0.001
s(C311)		2.741	70.003	< 0.001
s(C312)		0.907	18.311	< 0.001
s(C313)		2.043	25.906	< 0.001
s(C320)		0.000	0.000	0.670
s(C321)		1.649	6.263	0.021
s(C330)		0.000	0.000	0.764
s(C410)		0.507	0.792	0.208
s(C511)		2.758	5.791	0.095

Great Spotted Woodpecker (*Dendrocopos major*) — Model C-P-GAM				
**Parametric terms**	**Estimate**	**Standard error**	**z-value**	**p-value**
Intercept	−3.562	0.245	−14.544	< 0.001
Year 1995	−0.514	0.338	−1.518	0.129
Year 1996	−0.714	0.361	−1.977	0.048
Year 1999	0.768	0.256	2.993	0.003
Year 2000	0.481	0.260	1.847	0.065
Year 2001	0.630	0.268	2.351	0.019
Year 2002	0.767	0.277	2.766	0.006
Year 2003	1.032	0.273	3.788	< 0.001
Year 2004	0.724	0.308	2.350	0.019
Year 2005	0.967	0.262	3.695	< 0.001
Year 2006	1.155	0.255	4.531	< 0.001
Year 2007	1.195	0.265	4.519	< 0.001
Year 2008	1.035	0.270	3.827	< 0.001
Year 2009	0.880	0.273	3.227	0.001
Year 2010	1.196	0.264	4.525	< 0.001
Year 2011	1.465	0.256	5.715	< 0.001
Year 2012	1.369	0.262	5.227	< 0.001
Year 2013	1.535	0.258	5.943	< 0.001
Year 2014	1.584	0.257	6.163	< 0.001
Year 2015	1.405	0.261	5.380	< 0.001
Year 2016	1.567	0.259	6.055	< 0.001
Year 2017	1.623	0.261	6.213	< 0.001
Year 2018	1.201	0.270	4.443	< 0.001
Year 2019	1.631	0.261	6.240	< 0.001
Sin	−0.025	0.032	−0.773	0.440
Cos	0.055	0.048	1.147	0.251
**Smooth terms**		**edf**	**Chi square**	**p-value**
s(X, Y)		2.984	116.397	< 0.001
s(Elevation)		2.976	124.603	< 0.001
s(Slope)		0.975	17.158	< 0.001
s(C110)		2.162	18.054	< 0.001
s(C112)		1.721	10.527	0.001
s(C211)		2.118	11.104	0.001
s(C213)		0.931	25.415	< 0.001
s(C221)		1.302	0.432	0.667
s(C222)		0.000	0.000	0.357
s(C223)		0.844	4.641	0.019
s(C224)		1.849	61.231	< 0.001
s(C231)		0.762	5.004	0.006
s(C311)		2.924	192.659	< 0.001
s(C312)		1.044	63.092	< 0.001
s(C313)		2.439	141.736	< 0.001
s(C320)		0.642	1.448	0.122
s(C321)		0.869	9.923	< 0.001
s(C330)		0.772	4.357	0.016
s(C410)		0.000	0.000	0.901
s(C511)		0.000	0.000	0.541

Eurasian Skylark (*Alauda arvensis*) — Model C-ZIP-GAM				
**Binomial component**				
**Parametric terms**	**Estimate**	**Standard error**	**z-value**	**p-value**
Intercept	0.168	0.018	9.125	< 0.001
**Smooth terms**		**edf**	**Chi square**	**p-value**
s(Elevation)		2.991	618.800	< 0.001
s(Urb_{2500})		1.121	295.900	< 0.001
s(For_{2500})		2.110	193.900	< 0.001
**Count component**				
**Parametric terms**	**Estimate**	**Standard error**	**z-value**	**p-value**
Intercept	−1.124	0.107	−10.466	< 0.001
Year 1995	−0.645	0.085	−7.623	< 0.001
Year 1996	−0.861	0.084	−10.286	< 0.001
Year 1999	−0.661	0.094	−7.067	< 0.001
Year 2000	−0.748	0.082	−9.161	< 0.001
Year 2001	−0.919	0.084	−10.953	< 0.001
Year 2002	−0.939	0.101	−9.250	< 0.001
Year 2003	−1.083	0.103	−10.488	< 0.001
Year 2004	−1.391	0.127	−10.973	< 0.001
Year 2005	−1.163	0.082	−14.132	< 0.001
Year 2006	−1.349	0.093	−14.544	< 0.001
Year 2007	−1.777	0.113	−15.709	< 0.001
Year 2008	−1.767	0.121	−14.652	< 0.001
Year 2009	−1.676	0.108	−15.491	< 0.001
Year 2010	−1.836	0.122	−15.035	< 0.001
Year 2011	−2.245	0.134	−16.783	< 0.001
Year 2012	−2.199	0.128	−17.136	< 0.001
Year 2013	−2.506	0.147	−17.029	< 0.001
Year 2014	−2.463	0.152	−16.191	< 0.001
Year 2015	−2.564	0.154	−16.608	< 0.001
Year 2016	−2.146	0.130	−16.560	< 0.001
Year 2017	−2.586	0.181	−14.321	< 0.001
Year 2018	−2.280	0.161	−14.195	< 0.001
Year 2019	−2.513	0.176	−14.259	< 0.001
Sin	0.020	0.026	0.793	0.428
Cos	0.040	0.038	1.063	0.288
**Smooth terms**		**edf**	**Chi square**	**p-value**
s(X, Y)		2.979	267.814	< 0.001
s(Elevation)		2.940	183.753	< 0.001
S(Slope)		2.553	170.265	< 0.001
s(C110)		1.048	81.880	< 0.001
s(C112)		2.736	50.882	< 0.001
s(C211)		2.742	62.335	< 0.001
s(C213)		1.671	31.182	< 0.001
s(C221)		1.955	6.344	0.029
s(C222)		0.001	0.000	0.871
s(C223)		0.878	5.259	0.014
s(C224)		2.638	40.149	< 0.001
s(C231)		0.992	44.294	< 0.001
s(C311)		1.849	117.667	< 0.001
s(C312)		2.820	54.836	< 0.001
s(C313)		2.941	32.039	< 0.001
s(C320)		1.635	42.053	< 0.001
s(C321)		2.641	104.323	< 0.001
s(C330)		2.376	59.652	< 0.001
s(C410)		0.884	8.908	0.001
s(C511)		0.246	0.389	0.201

Eurasian Crag Martin (*Ptyonoprogne rupestris*) — Model C-NB-GAM				
**Parametric terms**	**Estimate**	**Standard error**	**z-value**	**p-value**
Intercept	−7.042	4.740	−1.486	0.137
Year 1995	0.577	0.576	1.001	0.317
Year 1996	0.237	0.595	0.397	0.691
Year 1999	0.350	0.548	0.639	0.523
Year 2000	0.047	0.548	0.085	0.932
Year 2001	0.983	0.547	1.797	0.072
Year 2002	0.384	0.624	0.616	0.538
Year 2003	0.561	0.580	0.967	0.334
Year 2004	0.041	0.653	0.062	0.950
Year 2005	−0.187	0.579	−0.323	0.747
Year 2006	0.237	0.566	0.419	0.675
Year 2007	−0.628	0.664	−0.946	0.344
Year 2008	−0.006	0.623	−0.009	0.993
Year 2009	0.102	0.612	0.167	0.867
Year 2010	0.490	0.582	0.841	0.401
Year 2011	0.650	0.557	1.167	0.243
Year 2012	0.092	0.584	0.157	0.875
Year 2013	0.906	0.554	1.637	0.102
Year 2014	0.456	0.566	0.806	0.420
Year 2015	0.895	0.548	1.634	0.102
Year 2016	0.895	0.553	1.619	0.105
Year 2017	0.474	0.606	0.782	0.434
Year 2018	0.268	0.618	0.433	0.665
Year 2019	0.841	0.580	1.449	0.147
Sin	−0.062	0.083	−0.746	0.455
Cos	−0.264	0.124	−2.132	0.033
**Smooth terms**		**edf**	**Chi square**	**p-value**
s(X, Y)		1.993	153.522	< 0.001
s(Elevation)		2.600	94.491	< 0.001
s(Slope)		2.778	68.499	< 0.001
s(C110)		1.881	4.913	0.055
s(C112)		2.191	19.357	< 0.001
s(C211)		1.119	19.901	< 0.001
s(C213)		0.717	0.048	0.795
s(C221)		0.847	4.982	0.015
s(C222)		0.897	6.973	0.005
s(C223)		0.872	4.835	0.018
s(C224)		0.000	0.000	0.920
s(C231)		1.109	21.828	< 0.001
s(C311)		2.174	64.035	< 0.001
s(C312)		2.057	41.727	< 0.001
s(C313)		1.453	52.537	< 0.001
s(C320)		0.939	11.846	< 0.001
s(C321)		0.967	14.382	< 0.001
s(C330)		0.337	0.451	0.235
s(C410)		0.001	0.001	0.361
s(C511)		0.877	6.802	0.005

Barn Swallow (*Hirundo rustica*) — Model C-ZINB-GAM				
**Binomial component**				
**Parametric terms**	**Estimate**	**Standard error**	**z-value**	**p-value**
Intercept	2.130	0.043	50.000	< 0.001
**Smooth terms**		**edf**	**Chi square**	**p-value**
s(Elevation)		1.248	1648.500	< 0.001
s(Urb_{2500})		2.925	697.400	< 0.001
s(For_{2500})		2.991	1062.300	< 0.001
**Count component**				
**Parametric terms**	**Estimate**	**Standard error**	**z-value**	**p-value**
Intercept	−0.224	0.097	−2.315	0.021
Year 1995	−0.432	0.101	−4.271	< 0.001
Year 1996	−0.245	0.098	−2.492	0.013
Year 1999	−0.074	0.097	−0.764	0.445
Year 2000	−0.158	0.092	−1.710	0.087
Year 2001	−0.366	0.095	−3.844	< 0.001
Year 2002	−0.294	0.107	−2.738	0.006
Year 2003	−0.556	0.110	−5.051	< 0.001
Year 2004	−0.656	0.127	−5.182	< 0.001
Year 2005	−0.697	0.095	−7.325	< 0.001
Year 2006	−0.567	0.095	−5.969	< 0.001
Year 2007	−0.838	0.104	−8.031	< 0.001
Year 2008	−1.053	0.108	−9.788	< 0.001
Year 2009	−1.040	0.104	−9.989	< 0.001
Year 2010	−0.595	0.101	−5.882	< 0.001
Year 2011	−0.998	0.101	−9.889	< 0.001
Year 2012	−1.343	0.108	−12.394	< 0.001
Year 2013	−0.910	0.103	−8.871	< 0.001
Year 2014	−1.155	0.104	−11.140	< 0.001
Year 2015	−1.169	0.105	−11.130	< 0.001
Year 2016	−0.765	0.103	−7.452	< 0.001
Year 2017	−1.153	0.117	−9.844	< 0.001
Year 2018	−0.858	0.113	−7.593	< 0.001
Year 2019	−0.908	0.114	−8.003	< 0.001
Sin	0.052	0.021	2.532	0.011
Cos	−0.172	0.031	−5.485	< 0.001
**Smooth terms**		**edf**	**Chi square**	**p-value**
s(X, Y)		1.951	63.796	< 0.001
s(Elevation)		2.887	94.594	< 0.001
S(Slope)		1.292	32.069	< 0.001
s(C110)		2.466	140.681	< 0.001
s(C112)		2.873	109.102	< 0.001
s(C211)		1.308	33.685	< 0.001
s(C213)		1.020	35.423	< 0.001
s(C221)		0.984	37.342	< 0.001
s(C222)		0.039	0.037	0.330
s(C223)		0.337	0.463	0.240
s(C224)		1.016	72.137	< 0.001
s(C231)		0.301	0.405	0.223
s(C311)		2.538	463.975	< 0.001
s(C312)		2.700	62.920	< 0.001
s(C313)		1.508	215.796	< 0.001
s(C320)		1.012	48.342	< 0.001
s(C321)		0.916	9.275	0.001
s(C330)		0.864	6.274	0.007
s(C410)		0.007	0.005	0.411
s(C511)		0.694	2.240	0.068

**Common House Martin (*Delichon urbicum*) — Model C-ZINB-GAM**				
**Binomial component**				
**Parametric terms**	**Estimate**	**Standard error**	**z-value**	**p-value**
Intercept	0.136	0.018	7.698	< 0.001
**Smooth terms**		**edf**	**Chi square**	**p-value**
s(Elevation)		1.183	776.100	< 0.001
s(Urb_{2500})		2.812	1310.600	< 0.001
s(For_{2500})		2.977	1117.300	< 0.001
**Count component**				
**Parametric terms**	**Estimate**	**Standard error**	**z-value**	**p-value**
Intercept	−0.209	0.160	−1.311	0.190
Year 1995	−0.734	0.203	−3.619	< 0.001
Year 1996	−0.091	0.192	−0.476	0.634
Year 1999	0.014	0.183	0.078	0.938
Year 2000	0.040	0.175	0.229	0.819
Year 2001	−0.042	0.184	−0.226	0.821
Year 2002	−0.042	0.202	−0.209	0.834
Year 2003	−0.079	0.202	−0.391	0.696
Year 2004	−0.567	0.230	−2.468	0.014
Year 2005	−0.553	0.185	−2.981	0.003
Year 2006	−0.210	0.179	−1.174	0.240
Year 2007	−0.405	0.195	−2.077	0.038
Year 2008	−0.494	0.196	−2.516	0.012
Year 2009	−0.827	0.198	−4.179	0.000
Year 2010	−0.117	0.191	−0.611	0.541
Year 2011	−0.469	0.186	−2.522	0.012
Year 2012	−0.598	0.194	−3.084	0.002
Year 2013	−0.733	0.194	−3.773	< 0.001
Year 2014	−0.819	0.189	−4.342	< 0.001
Year 2015	−0.840	0.193	−4.350	< 0.001
Year 2016	−0.813	0.195	−4.164	< 0.001
Year 2017	−1.016	0.216	−4.708	< 0.001
Year 2018	−0.865	0.212	−4.073	< 0.001
Year 2019	−0.433	0.205	−2.110	0.035
Sin	0.124	0.036	3.460	0.001
Cos	−0.097	0.054	−1.805	0.071
**Smooth terms**		**edf**	**Chi square**	**p-value**
s(X, Y)		1.861	38.888	< 0.001
s(Elevation)		0.006	0.004	0.384
s(Slope)		0.714	2.206	0.072
s(C110)		2.675	43.802	< 0.001
s(C112)		1.059	52.284	< 0.001
s(C211)		1.131	22.634	< 0.001
s(C213)		0.402	0.603	0.215
s(C221)		0.420	0.634	0.216
s(C222)		0.002	0.001	0.409
s(C223)		0.001	0.000	0.751
s(C224)		0.902	8.998	0.001
s(C231)		0.001	0.000	0.478
s(C311)		2.327	194.343	< 0.001
s(C312)		1.132	78.190	< 0.001
s(C313)		1.069	56.371	< 0.001
s(C320)		0.000	0.000	0.670
s(C321)		0.889	7.069	0.004
s(C330)		0.846	5.084	0.014
s(C410)		0.002	0.000	0.646
s(C511)		0.698	2.184	0.075

**Tree Pipit (*Anthus trivialis*) — Model C-ZIP-GAM**				
**Binomial component**				
**Parametric terms**	**Estimate**	**Standard error**	**z-value**	**p-value**
Intercept	−2.504	0.041	−61.520	< 0.001
**Smooth terms**		**edf**	**Chi square**	**p-value**
s(Elevation)		2.999	2002.800	< 0.001
s(Urb_{2500})		2.935	169.500	< 0.001
s(For_{2500})		2.930	1322.400	< 0.001
**Count component**				
**Parametric terms**	**Estimate**	**Standard error**	**z-value**	**p-value**
Intercept	−4.531	0.601	−7.537	< 0.001
Year 1995	−0.954	0.392	−2.433	0.015
Year 1996	−0.717	0.351	−2.042	0.041
Year 1999	−0.417	0.294	−1.422	0.155
Year 2000	−0.480	0.286	−1.676	0.094
Year 2001	−0.590	0.301	−1.962	0.050
Year 2002	−0.446	0.308	−1.448	0.148
Year 2003	−0.560	0.305	−1.833	0.067
Year 2004	−0.464	0.312	−1.491	0.136
Year 2005	−0.054	0.286	−0.189	0.850
Year 2006	−0.357	0.300	−1.191	0.234
Year 2007	−0.403	0.293	−1.378	0.168
Year 2008	0.068	0.284	0.241	0.810
Year 2009	−0.243	0.294	−0.828	0.408
Year 2010	−0.529	0.305	−1.734	0.083
Year 2011	−0.125	0.285	−0.436	0.663
Year 2012	−0.062	0.288	−0.216	0.829
Year 2013	−0.419	0.294	−1.425	0.154
Year 2014	−0.272	0.288	−0.944	0.345
Year 2015	−0.497	0.298	−1.670	0.095
Year 2016	−0.436	0.293	−1.489	0.136
Year 2017	−0.465	0.299	−1.554	0.120
Year 2018	−0.461	0.298	−1.546	0.122
Year 2019	−0.513	0.300	−1.708	0.088
Sin	−0.073	0.039	−1.847	0.065
Cos	0.191	0.062	3.091	0.002
**Smooth terms**		**edf**	**Chi square**	**p-value**
s(X, Y)		2.934	95.377	< 0.001
s(Elevation)		2.829	81.805	< 0.001
s(Slope)		2.486	25.543	< 0.001
s(C110)		0.658	1.832	0.094
s(C112)		1.843	13.770	< 0.001
s(C211)		0.000	0.000	0.683
s(C213)		0.000	0.000	0.778
s(C221)		0.760	3.863	0.023
s(C222)		0.723	0.541	0.387
s(C223)		0.000	0.000	0.862
s(C224)		0.001	0.000	0.941
s(C231)		2.159	207.516	< 0.001
s(C311)		0.001	0.000	0.583
s(C312)		2.839	24.556	< 0.001
s(C313)		2.269	20.518	< 0.001
s(C320)		0.005	0.000	0.871
s(C321)		2.842	252.486	< 0.001
s(C330)		1.916	13.918	< 0.001
s(C410)		0.196	0.249	0.259
s(C511)		0.946	17.296	< 0.001

**Water Pipit (*Anthus spinoletta*) — Model C-ZINB-GAM**				
**Binomial component**				
**Parametric terms**	**Estimate**	**Standard error**	**z-value**	**p-value**
Intercept	−2.936	0.047	−62.850	< 0.001
**Smooth terms**		**edf**	**Chi square**	**p-value**
s(Elevation)		2.957	2245.000	< 0.001
s(Urb_{2500})		0.000	0.000	0.927
s(For_{2500})		2.989	193.600	< 0.001
**Count component**				
**Parametric terms**	**Estimate**	**Standard error**	**z-value**	**p-value**
Intercept	−6.617	1.323	−5.003	< 0.001
Year 1995	0.271	0.432	0.628	0.530
Year 1996	0.028	0.445	0.064	0.949
Year 1999	−0.469	0.466	−1.007	0.314
Year 2000	−0.444	0.427	−1.039	0.299
Year 2001	−0.388	0.434	−0.894	0.371
Year 2002	−0.210	0.428	−0.490	0.624
Year 2003	−0.763	0.433	−1.764	0.078
Year 2004	−0.513	0.432	−1.188	0.235
Year 2005	−0.258	0.422	−0.612	0.541
Year 2006	−0.584	0.434	−1.347	0.178
Year 2007	−0.156	0.429	−0.363	0.717
Year 2008	0.014	0.425	0.032	0.974
Year 2009	0.064	0.424	0.150	0.881
Year 2010	−0.094	0.430	−0.219	0.827
Year 2011	0.006	0.428	0.013	0.989
Year 2012	−0.273	0.425	−0.643	0.520
Year 2013	−0.187	0.428	−0.438	0.662
Year 2014	−0.240	0.427	−0.563	0.574
Year 2015	−0.098	0.425	−0.230	0.818
Year 2016	−0.270	0.429	−0.630	0.529
Year 2017	−0.258	0.429	−0.601	0.548
Year 2018	−0.149	0.428	−0.348	0.728
Year 2019	−0.203	0.429	−0.472	0.637
Sin	−0.124	0.043	−2.911	0.004
Cos	−0.049	0.063	−0.766	0.444
**Smooth terms**		**edf**	**Chi square**	**p-value**
s(X, Y)		1.989	195.963	< 0.001
s(Elevation)		2.335	121.779	< 0.001
s(Slope)		1.841	44.518	< 0.001
s(C110)		0.324	0.510	0.208
s(C112)		0.735	2.662	0.052
s(C211)		0.000	0.000	0.644
s(C213)		0.000	0.000	0.872
s(C221)		0.349	1.046	0.079
s(C222)		0.000	0.000	0.806
s(C223)		0.000	0.000	0.967
s(C224)		0.000	0.000	0.630
s(C231)		0.000	0.000	0.646
s(C311)		0.764	2.799	0.048
s(C312)		1.235	163.133	< 0.001
s(C313)		1.026	23.254	< 0.001
s(C320)		0.920	9.697	< 0.001
s(C321)		1.034	21.497	< 0.001
s(C330)		0.578	1.359	0.111
s(C410)		0.284	0.422	0.222
s(C511)		0.842	5.120	0.014

Western Yellow Wagtail (*Motacilla flava*) — Model C-ZIP-GAM				
**Binomial component**				
**Parametric terms**	**Estimate**	**Standard error**	**z-value**	**p-value**
Intercept	−0.986	0.024	−41.700	< 0.001
**Smooth terms**		**edf**	**Chi square**	**p-value**
s(Elevation)		2.869	798.400	< 0.001
s(Urb_{2500})		1.177	767.400	< 0.001
s(For_{2500})		2.989	1371.500	< 0.001
**Count component**				
**Parametric terms**	**Estimate**	**Standard error**	**z-value**	**p-value**
Intercept	−3.260	0.337	−9.663	< 0.001
Year 1995	−0.350	0.133	−2.640	0.008
Year 1996	−0.467	0.129	−3.624	< 0.001
Year 1999	−0.232	0.142	−1.638	0.101
Year 2000	−0.087	0.119	−0.732	0.464
Year 2001	−0.291	0.120	−2.422	0.015
Year 2002	−0.273	0.139	−1.969	0.049
Year 2003	−0.223	0.133	−1.681	0.093
Year 2004	−0.596	0.156	−3.823	< 0.001
Year 2005	−0.425	0.117	−3.626	< 0.001
Year 2006	−0.373	0.123	−3.029	0.002
Year 2007	−0.524	0.133	−3.947	< 0.001
Year 2008	−0.499	0.138	−3.620	< 0.001
Year 2009	−0.361	0.125	−2.899	0.004
Year 2010	−0.564	0.139	−4.046	< 0.001
Year 2011	−0.686	0.137	−5.020	< 0.001
Year 2012	−0.936	0.145	−6.435	< 0.001
Year 2013	−0.487	0.133	−3.670	< 0.001
Year 2014	−1.250	0.172	−7.266	< 0.001
Year 2015	−0.731	0.143	−5.123	< 0.001
Year 2016	−0.864	0.147	−5.894	< 0.001
Year 2017	−0.698	0.161	−4.335	< 0.001
Year 2018	−0.511	0.158	−3.242	0.001
Year 2019	−0.656	0.160	−4.098	0.000
Sin	−0.031	0.028	−1.114	0.265
Cos	0.127	0.040	3.138	0.002
**Smooth terms**		**edf**	**Chi square**	**p-value**
s(X, Y)		1.386	36.306	< 0.001
s(Elevation)		0.001	0.001	0.189
s(Slope)		0.989	31.566	< 0.001
s(C110)		2.583	63.728	< 0.001
s(C112)		1.796	46.375	< 0.001
s(C211)		2.971	113.624	< 0.001
s(C213)		0.704	1.437	0.145
s(C221)		0.972	12.057	< 0.001
s(C222)		0.001	0.000	0.616
s(C223)		0.721	1.679	0.127
s(C224)		2.243	30.767	< 0.001
s(C231)		2.569	26.944	< 0.001
s(C311)		1.147	62.737	< 0.001
s(C312)		0.830	3.497	0.040
s(C313)		0.859	4.196	0.026
s(C320)		2.746	27.263	< 0.001
s(C321)		0.819	2.882	0.060
s(C330)		1.803	7.215	0.015
s(C410)		0.873	3.647	0.039
s(C511)		0.993	8.382	0.003

Grey Wagtail (*Motacilla cinerea*) — Model C-P-GAM				
**Parametric terms**	**Estimate**	**Standard error**	**z-value**	**p-value**
Intercept	−5.006	0.335	−14.950	< 0.001
Year 1995	0.417	0.367	1.137	0.255
Year 1996	0.555	0.363	1.527	0.127
Year 1999	−0.028	0.357	−0.078	0.938
Year 2000	0.818	0.334	2.446	0.014
Year 2001	0.527	0.358	1.470	0.142
Year 2002	0.293	0.398	0.735	0.462
Year 2003	0.056	0.390	0.142	0.887
Year 2004	0.150	0.411	0.365	0.715
Year 2005	0.198	0.364	0.543	0.587
Year 2006	0.251	0.364	0.690	0.490
Year 2007	0.344	0.377	0.911	0.362
Year 2008	0.313	0.375	0.835	0.404
Year 2009	0.444	0.372	1.195	0.232
Year 2010	0.347	0.375	0.927	0.354
Year 2011	0.032	0.379	0.085	0.932
Year 2012	0.293	0.373	0.785	0.433
Year 2013	0.263	0.365	0.720	0.472
Year 2014	0.389	0.367	1.060	0.289
Year 2015	0.183	0.363	0.506	0.613
Year 2016	0.349	0.360	0.969	0.332
Year 2017	0.312	0.388	0.804	0.421
Year 2018	0.334	0.382	0.875	0.382
Year 2019	0.166	0.390	0.425	0.671
Sin	−0.042	0.059	−0.706	0.481
Cos	−0.428	0.094	−4.546	< 0.001
**Smooth terms**		**edf**	**Chi square**	**p-value**
s(X,Y)		2.943	74.973	< 0.001
s(Elevation)		2.808	11.297	0.007
s(Slope)		2.924	42.278	< 0.001
s(C110)		2.866	10.761	0.010
s(C112)		2.051	15.864	< 0.001
s(C211)		0.001	0.000	0.489
s(C213)		0.566	1.463	0.107
s(C221)		0.763	2.947	0.049
s(C222)		0.740	2.168	0.087
s(C223)		0.700	1.529	0.139
s(C224)		0.001	0.000	0.833
s(C231)		0.001	0.001	0.332
s(C311)		2.871	11.252	0.008
s(C312)		2.881	50.661	< 0.001
s(C313)		1.623	2.997	0.149
s(C320)		1.465	1.151	0.426
s(C321)		0.001	0.001	0.412
s(C330)		2.673	34.371	< 0.001
s(C410)		0.001	0.000	0.593
s(C511)		2.958	407.525	< 0.001

White Wagtail (*Motacilla alba*) — Model C-P-GAM				
**Parametric terms**	**Estimate**	**Standard error**	**z-value**	**p-value**
Intercept	−2.005	0.137	−14.678	< 0.001
Year 1995	−0.686	0.186	−3.690	< 0.001
Year 1996	−0.623	0.177	−3.531	< 0.001
Year 1999	−0.610	0.161	−3.794	< 0.001
Year 2000	−0.318	0.153	−2.088	0.037
Year 2001	−0.280	0.160	−1.754	0.079
Year 2002	−0.341	0.179	−1.908	0.056
Year 2003	−0.486	0.175	−2.778	0.005
Year 2004	−0.617	0.199	−3.095	0.002
Year 2005	−0.795	0.167	−4.768	< 0.001
Year 2006	−0.654	0.165	−3.969	< 0.001
Year 2007	−0.723	0.182	−3.979	< 0.001
Year 2008	−0.820	0.187	−4.382	< 0.001
Year 2009	−0.875	0.188	−4.659	< 0.001
Year 2010	−0.965	0.189	−5.119	< 0.001
Year 2011	−0.590	0.166	−3.560	< 0.001
Year 2012	−0.991	0.187	−5.298	< 0.001
Year 2013	−0.573	0.168	−3.407	< 0.001
Year 2014	−0.753	0.171	−4.398	< 0.001
Year 2015	−0.829	0.174	−4.762	< 0.001
Year 2016	−0.584	0.167	−3.503	< 0.001
Year 2017	−0.979	0.201	−4.865	< 0.001
Year 2018	−0.696	0.186	−3.739	< 0.001
Year 2019	−0.636	0.182	−3.492	< 0.001
Sin	−0.059	0.034	−1.757	0.079
Cos	0.073	0.051	1.438	0.150
**Smooth terms**		**edf**	**Chi square**	**p-value**
s(X,Y)		1.905	75.686	< 0.001
s(Elevation)		2.893	120.572	< 0.001
s(Slope)		2.656	45.114	< 0.001
s(C110)		1.929	6.750	0.022
s(C112)		2.270	29.553	< 0.001
s(C211)		0.920	10.535	< 0.001
s(C213)		0.884	7.721	0.003
s(C221)		1.236	14.036	< 0.001
s(C222)		0.901	6.280	0.008
s(C223)		0.609	1.197	0.160
s(C224)		0.834	5.396	0.011
s(C231)		0.000	0.000	0.616
s(C311)		2.472	205.990	< 0.001
s(C312)		2.142	172.025	< 0.001
s(C313)		2.055	132.343	< 0.001
s(C320)		1.015	76.723	< 0.001
s(C321)		2.781	41.179	< 0.001
s(C330)		2.076	41.001	< 0.001
s(C410)		0.001	0.000	0.447
s(C511)		2.761	189.471	< 0.001

**Eurasian Wren (*Troglodytes troglodytes*) — Model C-ZIP-GAM**				
**Binomial component**				
**Parametric terms**	**Estimate**	**Standard error**	**z-value**	**p-value**
Intercept	0.648	0.052	12.550	< 0.001
**Smooth terms**		**edf**	**Chi square**	**p-value**
s(Elevation)		2.992	1194.390	< 0.001
s(Urb_{2500})		2.927	97.270	< 0.001
s(For_{2500})		2.990	1050.900	< 0.001
**Count component**				
**Parametric terms**	**Estimate**	**Standard error**	**z-value**	**p-value**
Intercept	−1.295	0.105	−12.325	< 0.001
Year 1995	−0.137	0.130	−1.058	0.290
Year 1996	−0.098	0.130	−0.753	0.452
Year 1999	0.070	0.109	0.644	0.520
Year 2000	0.085	0.106	0.799	0.425
Year 2001	0.213	0.113	1.883	0.060
Year 2002	0.033	0.124	0.265	0.791
Year 2003	−0.097	0.127	−0.761	0.447
Year 2004	−0.102	0.140	−0.728	0.466
Year 2005	−0.245	0.122	−2.015	0.044
Year 2006	−0.277	0.121	−2.284	0.022
Year 2007	−0.376	0.132	−2.857	0.004
Year 2008	0.050	0.120	0.413	0.679
Year 2009	−0.352	0.132	−2.665	0.008
Year 2010	−0.249	0.130	−1.920	0.055
Year 2011	−0.058	0.118	−0.497	0.619
Year 2012	−0.314	0.129	−2.436	0.015
Year 2013	−0.516	0.132	−3.898	< 0.001
Year 2014	−0.359	0.128	−2.798	0.005
Year 2015	0.020	0.119	0.171	0.865
Year 2016	0.058	0.118	0.487	0.626
Year 2017	0.196	0.122	1.605	0.108
Year 2018	−0.018	0.127	−0.138	0.890
Year 2019	−0.218	0.133	−1.640	0.101
Sin	−0.083	0.022	−3.805	< 0.001
Cos	−0.091	0.032	−2.849	0.004
**Smooth terms**		**edf**	**Chi square**	**p-value**
s(X, Y)		1.336	4.695	0.029
s(Elevation)		2.999	114.151	< 0.001
s(Slope)		2.704	72.199	< 0.001
s(C110)		2.913	13.473	0.002
s(C112)		0.619	2.201	0.050
s(C211)		0.754	4.787	0.008
s(C213)		0.001	0.000	0.762
s(C221)		0.703	2.316	0.066
s(C222)		0.002	0.001	0.575
s(C223)		0.882	5.962	0.009
s(C224)		1.843	23.095	< 0.001
s(C231)		1.710	38.390	< 0.001
s(C311)		2.609	183.394	< 0.001
s(C312)		2.926	208.747	< 0.001
s(C313)		2.097	180.425	< 0.001
s(C320)		2.615	94.183	< 0.001
s(C321)		2.603	102.242	< 0.001
s(C330)		0.971	35.375	0.000
s(C410)		0.001	0.000	0.613
s(C511)		2.884	121.164	< 2e−16

Dunnock (*Prunella modularis*) — Model C-P-GAM				
**Parametric terms**	**Estimate**	**Standard error**	**z-value**	**p-value**
Intercept	−7.917	3.509	−2.256	0.024
Year 1995	−1.697	0.559	−3.037	0.002
Year 1996	0.286	0.393	0.726	0.468
Year 1999	−0.151	0.373	−0.405	0.686
Year 2000	0.254	0.362	0.700	0.484
Year 2001	0.419	0.364	1.153	0.249
Year 2002	0.783	0.368	2.124	0.034
Year 2003	0.131	0.372	0.352	0.725
Year 2004	0.259	0.384	0.674	0.500
Year 2005	0.287	0.357	0.803	0.422
Year 2006	0.241	0.381	0.632	0.528
Year 2007	0.285	0.364	0.785	0.433
Year 2008	0.275	0.360	0.765	0.444
Year 2009	0.428	0.360	1.191	0.234
Year 2010	0.374	0.362	1.032	0.302
Year 2011	0.512	0.354	1.447	0.148
Year 2012	0.699	0.352	1.985	0.047
Year 2013	0.679	0.351	1.936	0.053
Year 2014	0.740	0.350	2.114	0.035
Year 2015	0.877	0.348	2.524	0.012
Year 2016	0.580	0.352	1.651	0.099
Year 2017	0.468	0.360	1.300	0.194
Year 2018	0.469	0.360	1.303	0.193
Year 2019	0.628	0.357	1.758	0.079
Sin	−0.134	0.041	−3.247	0.001
Cos	0.001	0.058	0.009	0.993
**Smooth terms**		**edf**	**Chi square**	**p-value**
s(X,Y)		1.864	17.912	< 0.001
s(Elevation)		2.982	299.735	< 0.001
s(Slope)		2.485	21.832	< 0.001
s(C110)		2.807	15.766	< 0.001
s(C112)		1.825	10.233	0.003
s(C211)		0.057	0.056	0.317
s(C213)		0.710	0.043	0.807
s(C221)		0.559	0.319	0.450
s(C222)		0.001	0.000	0.743
s(C223)		0.000	0.000	0.867
s(C224)		0.001	0.000	0.492
s(C231)		2.245	17.772	< 0.001
s(C311)		0.894	8.162	0.002
s(C312)		2.778	32.628	< 0.001
s(C313)		2.239	7.858	0.018
s(C320)		2.255	40.383	< 0.001
s(C321)		2.433	35.524	< 0.001
s(C330)		0.002	0.001	0.493
s(C410)		0.004	0.003	0.396
s(C511)		0.001	0.001	0.435

European Robin (*Erithacus rubecula*) — Model C-ZIP-GAM				
**Binomial component**				
**Parametric terms**	**Estimate**	**Standard error**	**z-value**	**p-value**
Intercept	−0.030	0.034	−0.875	0.382
**Smooth terms**		**edf**	**Chi square**	**p-value**
s(Elevation)		2.985	515.800	< 0.001
s(Urb_{2500})		2.963	441.300	< 0.001
s(For_{2500})		2.976	1563.600	< 0.001
**Count component**				
**Parametric terms**	**Estimate**	**Standard error**	**z-value**	**p-value**
Intercept	−2.284	0.140	−16.281	< 0.001
Year 1995	0.016	0.142	0.115	0.909
Year 1996	0.173	0.138	1.261	0.207
Year 1999	0.285	0.115	2.467	0.014
Year 2000	0.221	0.113	1.948	0.051
Year 2001	0.497	0.118	4.205	< 0.001
Year 2002	0.300	0.123	2.440	0.015
Year 2003	0.056	0.132	0.425	0.671
Year 2004	0.090	0.143	0.628	0.530
Year 2005	0.267	0.123	2.166	0.030
Year 2006	0.115	0.123	0.937	0.349
Year 2007	0.316	0.126	2.513	0.012
Year 2008	0.139	0.131	1.062	0.288
Year 2009	0.054	0.134	0.404	0.686
Year 2010	0.076	0.135	0.562	0.574
Year 2011	0.355	0.122	2.904	0.004
Year 2012	0.106	0.131	0.805	0.421
Year 2013	−0.002	0.133	−0.012	0.990
Year 2014	0.194	0.128	1.512	0.131
Year 2015	0.410	0.125	3.285	0.001
Year 2016	0.415	0.124	3.355	0.001
Year 2017	0.417	0.130	3.215	< 0.001
Year 2018	0.402	0.131	3.070	0.002
Year 2019	0.152	0.138	1.101	0.271
Sin	0.020	0.020	1.012	0.312
Cos	−0.024	0.029	−0.821	0.411
**Smooth terms**		**edf**	**Chi square**	**p-value**
s(X, Y)		2.900	159.031	< 0.001
s(Elevation)		2.868	146.902	< 0.001
s(Slope)		0.001	0.000	0.594
s(C110)		1.057	96.924	< 0.001
s(C112)		1.031	93.059	< 0.001
s(C211)		2.189	42.451	< 0.001
s(C213)		0.937	10.092	< 0.001
s(C221)		0.001	0.000	0.889
s(C222)		0.001	0.000	0.604
s(C223)		0.870	5.067	0.016
s(C224)		0.181	0.240	0.248
s(C231)		0.001	0.000	0.565
s(C311)		2.551	399.320	< 0.001
s(C312)		1.388	376.424	< 0.001
s(C313)		2.147	412.555	< 0.001
s(C320)		2.152	58.493	< 0.001
s(C321)		2.818	26.209	< 0.001
s(C330)		1.208	1.970	0.189
s(C410)		0.882	6.815	0.005
s(C511)		1.817	10.741	0.003

Common Nigthingale (*Luscinia megarhynchos*) — Model C-ZIP-GAM				
**Binomial component**				
**Parametric terms**	**Estimate**	**Standard error**	**z-value**	**p-value**
Intercept	1.323	0.041	32.000	< 0.001
**Smooth terms**		**edf**	**Chi square**	**p-value**
s(Elevation)		2.991	2115.900	< 0.001
s(Urb_{2500})		2.991	1970.500	< 0.001
s(For_{2500})		2.651	936.900	< 0.001
**Count component**				
**Parametric terms**	**Estimate**	**Standard error**	**z-value**	**p-value**
Intercept	−1.057	0.106	−9.924	< 0.001
Year 1995	−0.546	0.065	−8.352	< 0.001
Year 1996	−0.562	0.069	−8.108	< 0.001
Year 1999	−0.441	0.063	−6.969	< 0.001
Year 2000	−0.518	0.062	−8.414	< 0.001
Year 2001	−0.665	0.064	−10.408	< 0.001
Year 2002	−0.802	0.081	−9.858	< 0.001
Year 2003	−0.615	0.078	−7.911	< 0.001
Year 2004	−0.594	0.095	−6.249	< 0.001
Year 2005	−0.488	0.062	−7.927	< 0.001
Year 2006	−0.592	0.064	−9.236	< 0.001
Year 2007	−0.300	0.066	−4.544	< 0.001
Year 2008	−0.390	0.070	−5.595	< 0.001
Year 2009	−0.473	0.068	−6.912	< 0.001
Year 2010	−0.457	0.068	−6.699	< 0.001
Year 2011	−0.623	0.071	−8.821	< 0.001
Year 2012	−0.699	0.074	−9.476	< 0.001
Year 2013	−0.473	0.068	−6.947	< 0.001
Year 2014	−0.537	0.072	−7.511	< 0.001
Year 2015	−0.722	0.075	−9.663	< 0.001
Year 2016	−0.594	0.072	−8.270	< 0.001
Year 2017	−0.460	0.076	−6.026	< 0.001
Year 2018	−0.496	0.077	−6.473	< 0.001
Year 2019	−0.435	0.076	−5.728	< 0.001
Sin	−0.046	0.016	−2.923	0.003
Cos	−0.007	0.024	−0.280	0.779
**Smooth terms**		**edf**	**Chi square**	**p-value**
s(X, Y)		2.957	37.354	< 0.001
s(Elevation)		2.114	110.467	< 0.001
s(Slope)		2.954	111.076	< 0.001
s(C110)		2.952	181.908	< 0.001
s(C112)		1.021	180.278	< 0.001
s(C211)		1.721	32.042	< 0.001
s(C213)		2.813	15.533	< 0.001
s(C221)		0.547	1.104	0.148
s(C222)		0.000	0.000	0.660
s(C223)		1.826	4.165	0.094
s(C224)		1.913	33.165	< 0.001
s(C231)		1.061	28.730	< 0.001
s(C311)		2.924	280.454	< 0.001
s(C312)		0.000	0.000	0.917
s(C313)		2.594	6.399	0.061
s(C320)		1.011	64.455	< 0.001
s(C321)		0.000	0.000	0.704
s(C330)		0.000	0.000	0.962
s(C410)		0.875	10.867	< 0.001
s(C511)		1.966	51.633	< 0.001

Black Redstart (*Phoenicurus ochruros*) — Model C-ZIP-GAM				
**Binomial component**				
**Parametric terms**	**Estimate**	**Standard error**	**z-value**	**p-value**
Intercept	−0.441	0.048	−9.120	< 0.001
**Smooth terms**		**edf**	**Chi square**	**p-value**
s(Elevation)		2.962	1147.600	< 0.001
s(Urb_{2500})		1.095	212.100	< 0.001
s(For_{2500})		2.342	27.200	< 0.001
**Count component**				
**Parametric terms**	**Estimate**	**Standard error**	**z-value**	**p-value**
Intercept	−3.120	0.326	−9.582	< 0.001
Year 1995	−0.020	0.365	−0.056	0.955
Year 1996	−0.196	0.389	−0.504	0.614
Year 1999	0.180	0.348	0.517	0.605
Year 2000	0.280	0.336	0.834	0.405
Year 2001	0.347	0.343	1.011	0.312
Year 2002	0.400	0.348	1.151	0.250
Year 2003	0.484	0.336	1.440	0.150
Year 2004	0.531	0.340	1.562	0.118
Year 2005	0.599	0.330	1.812	0.070
Year 2006	0.343	0.341	1.006	0.314
Year 2007	0.562	0.338	1.662	0.097
Year 2008	0.443	0.338	1.312	0.190
Year 2009	0.551	0.337	1.633	0.102
Year 2010	0.502	0.338	1.487	0.137
Year 2011	0.732	0.332	2.204	0.028
Year 2012	0.621	0.333	1.866	0.062
Year 2013	0.712	0.332	2.141	0.032
Year 2014	0.778	0.329	2.365	0.018
Year 2015	0.534	0.332	1.605	0.109
Year 2016	0.597	0.332	1.799	0.072
Year 2017	0.440	0.340	1.296	0.195
Year 2018	0.653	0.337	1.934	0.053
Year 2019	0.505	0.339	1.488	0.137
Sin	−0.009	0.034	−0.265	0.791
Cos	0.017	0.052	0.320	0.749
**Smooth terms**		**edf**	**Chi square**	**p-value**
s(X, Y)		1.771	27.221	< 0.001
s(Elevation)		2.906	186.283	< 0.001
s(Slope)		0.000	0.000	0.540
s(C110)		1.767	82.392	< 0.001
s(C112)		2.788	157.183	< 0.001
s(C211)		0.000	0.000	0.830
s(C213)		0.000	0.000	0.806
s(C221)		0.031	0.032	0.306
s(C222)		0.001	0.000	0.466
s(C223)		0.658	1.125	0.191
s(C224)		0.000	0.000	0.553
s(C231)		1.921	10.728	< 0.001
s(C311)		2.178	52.404	< 0.001
s(C312)		2.180	39.294	< 0.001
s(C313)		2.559	34.261	< 0.001
s(C320)		2.266	14.930	< 0.001
s(C321)		0.815	3.559	0.032
s(C330)		2.471	53.138	< 0.001
s(C410)		0.464	0.827	0.180
s(C511)		0.670	2.013	0.082

**Common Redstart (*Phoenicurus Phoenicurus*) — Model C-ZIP-GAM**				
**Binomial component**				
**Parametric terms**	**Estimate**	**Standard error**	**z-value**	**p-value**
Intercept	0.268	0.024	11.020	< 0.001
**Smooth terms**		**edf**	**Chi square**	**p-value**
s(Elevation)		2.999	1672.400	< 0.001
s(Urb_{2500})		0.981	24.140	< 0.001
s(For_{2500})		2.335	133.800	< 0.001
**Count component**				
**Parametric terms**	**Estimate**	**Standard error**	**z-value**	**p-value**
Intercept	−2.772	0.213	−12.989	< 0.001
Year 1995	0.327	0.250	1.307	0.191
Year 1996	−0.224	0.262	−0.854	0.393
Year 1999	0.594	0.222	2.677	0.007
Year 2000	0.923	0.215	4.288	< 0.001
Year 2001	0.753	0.226	3.329	< 0.001
Year 2002	0.675	0.238	2.840	0.005
Year 2003	0.415	0.246	1.688	0.091
Year 2004	0.240	0.276	0.870	0.384
Year 2005	0.868	0.221	3.925	< 0.001
Year 2006	0.716	0.222	3.220	0.001
Year 2007	0.824	0.226	3.647	< 0.001
Year 2008	0.867	0.228	3.796	< 0.001
Year 2009	0.644	0.234	2.751	0.006
Year 2010	0.792	0.227	3.481	< 0.001
Year 2011	0.865	0.220	3.940	< 0.001
Year 2012	0.826	0.224	3.694	< 0.001
Year 2013	1.004	0.220	4.559	< 0.001
Year 2014	0.995	0.218	4.558	< 0.001
Year 2015	0.836	0.224	3.740	< 0.001
Year 2016	0.666	0.225	2.968	0.003
Year 2017	0.667	0.237	2.819	0.005
Year 2018	0.541	0.241	2.245	0.025
Year 2019	0.642	0.236	2.716	0.007
Sin	−0.077	0.030	−2.588	0.010
Cos	0.029	0.045	0.657	0.511
**Smooth terms**		**edf**	**Chi square**	**p-value**
s(X, Y)		1.290	4.697	0.025
s(Elevation)		2.548	59.882	< 0.001
s(Slope)		2.524	53.958	< 0.001
s(C110)		1.107	243.910	< 0.001
s(C112)		2.924	312.361	< 0.001
s(C211)		0.133	0.249	0.163
s(C213)		0.878	7.432	0.003
s(C221)		2.267	85.016	< 0.001
s(C222)		0.872	10.295	< 0.001
s(C223)		1.783	2.915	0.185
s(C224)		0.000	0.000	0.777
s(C231)		2.325	118.859	< 0.001
s(C311)		2.707	35.278	< 0.001
s(C312)		0.002	0.000	0.853
s(C313)		1.873	5.090	0.053
s(C320)		0.833	5.676	0.008
s(C321)		2.738	44.594	< 0.001
s(C330)		0.011	0.011	0.314
s(C410)		0.892	7.395	0.004
s(C511)		0.679	1.978	0.087

African Stonechat (*Saxicola torquatus*) — Model C-ZIP-GAM				
**Binomial component**				
**Parametric terms**	**Estimate**	**Standard error**	**z-value**	**p-value**
Intercept	−1.162	0.020	−57.030	< 0.001
**Smooth terms**		**edf**	**Chi square**	**p-value**
s(Elevation)		1.127	494.500	< 0.001
s(Urb_{2500})		0.002	0.000	1.000
s(For_{2500})		2.943	451.500	< 0.001
**Count component**				
**Parametric terms**	**Estimate**	**Standard error**	**z-value**	**p-value**
Intercept	−2.886	0.258	−11.180	< 0.001
Year 1995	−0.337	0.316	−1.067	0.286
Year 1996	0.550	0.273	2.013	0.044
Year 1999	0.506	0.281	1.796	0.073
Year 2000	0.538	0.265	2.027	0.043
Year 2001	0.769	0.265	2.901	0.004
Year 2002	0.568	0.294	1.934	0.053
Year 2003	0.443	0.303	1.461	0.144
Year 2004	0.813	0.298	2.724	0.006
Year 2005	0.742	0.259	2.870	0.004
Year 2006	−0.485	0.308	−1.574	0.116
Year 2007	0.205	0.287	0.715	0.475
Year 2008	0.047	0.300	0.158	0.874
Year 2009	−0.751	0.359	−2.092	0.036
Year 2010	−0.369	0.326	−1.132	0.258
Year 2011	−1.109	0.366	−3.032	0.002
Year 2012	−1.311	0.410	−3.197	0.001
Year 2013	−0.418	0.320	−1.307	0.191
Year 2014	−0.851	0.375	−2.273	0.023
Year 2015	−0.580	0.336	−1.728	0.084
Year 2016	−0.645	0.340	−1.894	0.058
Year 2017	−1.150	0.447	−2.576	0.010
Year 2018	−0.670	0.384	−1.745	0.081
Year 2019	−1.455	0.507	−2.871	0.004
Sin	0.033	0.056	0.592	0.554
Cos	−0.002	0.083	−0.026	0.979
**Smooth terms**		**edf**	**Chi square**	**p-value**
s(X, Y)		2.970	95.359	< 0.001
s(Elevation)		2.784	76.100	< 0.001
s(Slope)		1.001	8.161	0.003
s(C110)		1.656	36.969	< 0.001
s(C112)		1.870	11.101	0.002
s(C211)		2.835	44.528	< 0.001
s(C213)		0.000	0.000	0.905
s(C221)		2.529	31.753	< 0.001
s(C222)		0.002	0.000	0.968
s(C223)		0.004	0.003	0.350
s(C224)		0.000	0.000	0.507
s(C231)		1.971	64.175	< 0.001
s(C311)		2.105	30.630	< 0.001
s(C312)		0.838	5.439	0.010
s(C313)		0.844	4.919	0.015
s(C320)		0.919	14.975	< 0.001
s(C321)		0.001	0.000	0.456
s(C330)		0.001	0.000	0.409
s(C410)		1.562	3.312	0.115
s(C511)		0.000	0.000	0.532

Northern Wheatear (*Oenanthe oenanthe*) — Model C-P-GAM				
**Parametric terms**	**Estimate**	**Standard error**	**z-value**	**p-value**
Intercept	−46.028	16.412	−2.805	0.005
Year 1995	−0.007	0.621	−0.011	0.991
Year 1996	−0.253	0.637	−0.397	0.691
Year 1999	−0.584	0.646	−0.904	0.366
Year 2000	−0.869	0.618	−1.407	0.159
Year 2001	−0.631	0.630	−1.001	0.317
Year 2002	−0.914	0.634	−1.442	0.149
Year 2003	−1.308	0.635	−2.058	0.040
Year 2004	−1.120	0.639	−1.753	0.080
Year 2005	−0.325	0.601	−0.541	0.589
Year 2006	−0.411	0.611	−0.672	0.502
Year 2007	−0.383	0.611	−0.626	0.531
Year 2008	−0.400	0.608	−0.658	0.510
Year 2009	−0.153	0.603	−0.253	0.800
Year 2010	−0.210	0.608	−0.346	0.729
Year 2011	−0.170	0.608	−0.279	0.780
Year 2012	−0.494	0.605	−0.817	0.414
Year 2013	−0.393	0.609	−0.646	0.518
Year 2014	−0.511	0.608	−0.840	0.401
Year 2015	−0.371	0.606	−0.611	0.541
Year 2016	−0.764	0.619	−1.234	0.217
Year 2017	−0.733	0.617	−1.188	0.235
Year 2018	−0.824	0.621	−1.327	0.184
Year 2019	−0.973	0.626	−1.554	0.120
Sin	−0.096	0.064	−1.504	0.132
Cos	−0.120	0.097	−1.245	0.213
**Smooth terms**		**edf**	**Chi square**	**p-value**
s(X,Y)		1.971	80.389	< 0.001
s(Elevation)		2.838	91.077	< 0.001
s(Slope)		1.647	26.696	< 0.001
s(C110)		0.002	0.000	0.930
s(C112)		1.890	6.191	0.034
s(C211)		0.661	0.703	0.302
s(C213)		0.000	0.000	1.000
s(C221)		1.258	15.182	< 0.001
s(C222)		0.000	0.000	0.929
s(C223)		0.000	0.000	0.967
s(C224)		0.001	0.000	0.755
s(C231)		0.001	0.001	0.315
s(C311)		1.054	15.231	< 0.001
s(C312)		2.455	50.942	< 0.001
s(C313)		0.960	13.382	< 0.001
s(C320)		2.072	5.880	0.041
s(C321)		1.779	22.435	< 0.001
s(C330)		2.350	4.676	0.106
s(C410)		0.002	0.001	0.560
s(C511)		0.772	3.066	0.046

**Common Blackbird (*Turdus merula*) — Model C-ZIP-GAM**				
**Binomial component**				
**Parametric terms**	**Estimate**	**Standard error**	**z-value**	**p-value**
Intercept	6.102	0.186	32.800	< 0.001
**Smooth terms**		**edf**	**Chi square**	**p-value**
s(Elevation)		2.973	614.400	< 0.001
s(Urb_{2500})		1.019	417.700	< 0.001
s(For_{2500})		2.970	300.700	< 0.001
**Count component**				
**Parametric terms**	**Estimate**	**Standard error**	**z-value**	**p-value**
Intercept	0.341	0.042	8.152	< 0.001
Year 1995	−0.366	0.055	−6.632	< 0.001
Year 1996	−0.437	0.055	−7.916	< 0.001
Year 1999	−0.150	0.048	−3.133	0.002
Year 2000	−0.465	0.048	−9.701	< 0.001
Year 2001	−0.382	0.051	−7.435	< 0.001
Year 2002	−0.410	0.057	−7.158	< 0.001
Year 2003	−0.517	0.061	−8.534	< 0.001
Year 2004	−0.671	0.071	−9.442	< 0.001
Year 2005	−0.514	0.052	−9.936	< 0.001
Year 2006	−0.491	0.050	−9.765	< 0.001
Year 2007	−0.434	0.055	−7.909	< 0.001
Year 2008	−0.442	0.055	−7.980	< 0.001
Year 2009	−0.242	0.052	−4.618	< 0.001
Year 2010	−0.300	0.053	−5.690	< 0.001
Year 2011	−0.339	0.051	−6.710	< 0.001
Year 2012	−0.337	0.053	−6.384	< 0.001
Year 2013	−0.290	0.052	−5.547	< 0.001
Year 2014	−0.312	0.051	−6.165	< 0.001
Year 2015	−0.284	0.052	−5.487	< 0.001
Year 2016	−0.176	0.051	−3.453	< 0.001
Year 2017	−0.102	0.054	−1.880	0.060
Year 2018	−0.161	0.055	−2.936	0.003
Year 2019	−0.125	0.054	−2.308	0.021
Sin	0.002	0.011	0.213	0.831
Cos	−0.018	0.016	−1.124	0.261
**Smooth terms**		**edf**	**Chi square**	**p-value**
s(X, Y)		2.741	18.702	< 0.001
s(Elevation)		2.940	517.923	< 0.001
s(Slope)		2.787	71.669	< 0.001
s(C110)		1.117	31.062	< 0.001
s(C112)		2.793	14.185	0.001
s(C211)		2.436	48.299	< 0.001
s(C213)		1.460	114.755	< 0.001
s(C221)		2.670	13.395	0.002
s(C222)		0.004	0.004	0.288
s(C223)		0.531	1.258	0.123
s(C224)		0.466	1.174	0.110
s(C231)		2.841	11.630	0.005
s(C311)		2.483	45.685	< 0.001
s(C312)		1.776	15.874	< 0.001
s(C313)		2.314	22.360	< 0.001
s(C320)		1.913	9.153	0.006
s(C321)		2.942	41.454	< 0.001
s(C330)		0.967	22.912	< 0.001
s(C410)		0.001	0.000	0.474
s(C511)		2.049	15.411	< 0.001

Song Thrush (*Turdus philomelos*) — Model C-ZIP-GAM				
**Binomial component**				
**Parametric terms**	**Estimate**	**Standard error**	**z-value**	**p-value**
Intercept	−3.425	0.084	−40.980	< 0.001
**Smooth terms**		**edf**	**Chi square**	**p-value**
s(Elevation)		1.314	2405.000	< 0.001
s(Urb_{2500})		0.000	0.000	0.564
s(For_{2500})		2.996	1408.000	< 0.001
**Count component**				
**Parametric terms**	**Estimate**	**Standard error**	**z-value**	**p-value**
Intercept	−4.322	0.822	−5.259	< 0.001
Year 1995	1.274	0.564	2.259	0.024
Year 1996	0.294	0.629	0.468	0.640
Year 1999	1.321	0.519	2.544	0.011
Year 2000	1.060	0.519	2.041	0.041
Year 2001	1.449	0.521	2.783	0.005
Year 2002	1.540	0.532	2.897	0.004
Year 2003	1.706	0.526	3.245	0.001
Year 2004	1.170	0.573	2.042	0.041
Year 2005	1.436	0.526	2.731	0.006
Year 2006	1.724	0.525	3.284	0.001
Year 2007	1.757	0.520	3.379	< 0.001
Year 2008	2.142	0.516	4.153	< 0.001
Year 2009	1.903	0.521	3.652	< 0.001
Year 2010	1.690	0.526	3.214	0.001
Year 2011	1.965	0.517	3.801	< 0.001
Year 2012	2.103	0.516	4.077	< 0.001
Year 2013	2.593	0.509	5.098	< 0.001
Year 2014	2.055	0.514	3.998	< 0.001
Year 2015	2.300	0.512	4.491	< 0.001
Year 2016	2.436	0.510	4.773	< 0.001
Year 2017	2.452	0.514	4.768	< 0.001
Year 2018	2.590	0.513	5.049	< 0.001
Year 2019	2.475	0.515	4.811	< 0.001
Sin	−0.007	0.039	−0.174	0.862
Cos	0.065	0.054	1.210	0.226
**Smooth terms**		**edf**	**Chi square**	**p-value**
s(X, Y)		2.999	114.672	< 0.001
s(Elevation)		2.254	37.870	< 0.001
s(Slope)		1.961	10.915	0.002
s(C110)		2.795	11.671	0.005
s(C112)		1.825	3.752	0.108
s(C211)		0.006	0.009	0.206
s(C213)		0.000	0.000	0.530
s(C221)		0.000	0.000	0.705
s(C222)		0.000	0.000	0.782
s(C223)		0.608	0.163	0.605
s(C224)		0.000	0.000	0.758
s(C231)		0.977	6.032	0.006
s(C311)		2.718	42.245	< 0.001
s(C312)		2.297	75.536	< 0.001
s(C313)		1.232	47.050	< 0.001
s(C320)		0.855	2.588	0.067
s(C321)		2.296	21.632	< 0.001
s(C330)		0.000	0.000	0.760
s(C410)		0.000	0.000	0.620
s(C511)		0.000	0.000	0.350

**Mistle Thrush (*Turdus viscivorus*) — Model C-ZIP-GAM**				
**Binomial component**				
**Parametric terms**	**Estimate**	**Standard error**	**z-value**	**p-value**
Intercept	−3.050	0.045	−67.660	< 0.001
**Smooth terms**		**edf**	**Chi square**	**p-value**
s(Elevation)		2.999	3103.800	< 0.001
s(Urb_{2500})		0.000	0.000	0.888
s(For_{2500})		1.437	186.200	< 0.001
**Count component**				
**Parametric terms**	**Estimate**	**Standard error**	**z-value**	**p-value**
Intercept	−13.327	45.476	−0.293	0.769
Year 1995	0.769	0.804	0.957	0.338
Year 1996	0.641	0.805	0.796	0.426
Year 1999	1.168	0.734	1.591	0.112
Year 2000	0.513	0.751	0.684	0.494
Year 2001	−0.337	0.820	−0.411	0.681
Year 2002	0.942	0.767	1.228	0.219
Year 2003	0.612	0.767	0.798	0.425
Year 2004	0.685	0.820	0.835	0.404
Year 2005	0.896	0.746	1.202	0.229
Year 2006	1.292	0.759	1.701	0.089
Year 2007	0.835	0.750	1.114	0.265
Year 2008	0.999	0.750	1.332	0.183
Year 2009	1.201	0.745	1.611	0.107
Year 2010	1.399	0.740	1.891	0.059
Year 2011	1.658	0.726	2.283	0.022
Year 2012	1.104	0.748	1.476	0.140
Year 2013	1.485	0.730	2.035	0.042
Year 2014	1.774	0.724	2.451	0.014
Year 2015	1.417	0.734	1.931	0.053
Year 2016	1.525	0.730	2.090	0.037
Year 2017	1.453	0.744	1.954	0.051
Year 2018	1.526	0.742	2.058	0.040
Year 2019	1.220	0.751	1.625	0.104
Sin	−0.195	0.069	−2.839	0.005
Cos	0.127	0.097	1.300	0.194
**Smooth terms**		**edf**	**Chi square**	**p-value**
s(X, Y)		1.085	2.118	0.138
s(Elevation)		2.754	25.728	< 0.001
s(Slope)		1.617	11.042	0.001
s(C110)		0.000	0.000	0.692
s(C112)		0.854	3.282	0.047
s(C211)		0.000	0.000	0.377
s(C213)		0.002	0.000	0.741
s(C221)		0.001	0.000	0.346
s(C222)		0.000	0.000	0.721
s(C223)		0.000	0.000	0.875
s(C224)		0.398	0.036	0.763
s(C231)		1.765	12.243	< 0.001
s(C311)		0.000	0.000	0.561
s(C312)		2.803	77.756	< 0.001
s(C313)		1.694	30.072	< 0.001
s(C320)		2.466	9.793	0.008
s(C321)		2.432	20.050	< 0.001
s(C330)		0.000	0.000	0.419
s(C410)		0.000	0.000	0.797
s(C511)		0.003	0.003	0.331

Cetti's Warbler (*Cettia cetti*) — Model C-ZIP-GAM				
**Binomial component**				
**Parametric terms**	**Estimate**	**Standard error**	**z-value**	**p-value**
Intercept	−1.328	0.026	−50.340	< 0.001
**Smooth terms**		**edf**	**Chi square**	**p-value**
s(Elevation)		1.148	533.500	< 0.001
s(Urb_{2500})		2.989	1014.300	< 0.001
s(For_{2500})		2.997	863.100	< 0.001
**Count component**				
**Parametric terms**	**Estimate**	**Standard error**	**z-value**	**p-value**
Intercept	−4.385	0.823	−5.325	< 0.001
Year 1995	−0.070	0.229	−0.305	0.761
Year 1996	0.138	0.245	0.565	0.572
Year 1999	0.561	0.221	2.542	0.011
Year 2000	0.537	0.222	2.421	0.015
Year 2001	0.925	0.216	4.284	< 0.001
Year 2002	−0.069	0.298	−0.230	0.818
Year 2003	−0.238	0.298	−0.796	0.426
Year 2004	0.624	0.275	2.264	0.024
Year 2005	0.419	0.219	1.913	0.056
Year 2006	−0.437	0.250	−1.753	0.080
Year 2007	0.078	0.250	0.310	0.757
Year 2008	0.438	0.241	1.820	0.069
Year 2009	−0.144	0.255	−0.566	0.572
Year 2010	−0.532	0.284	−1.873	0.061
Year 2011	−0.187	0.260	−0.721	0.471
Year 2012	−0.726	0.306	−2.371	0.018
Year 2013	−0.960	0.316	−3.040	0.002
Year 2014	−0.834	0.322	−2.592	0.010
Year 2015	−0.320	0.271	−1.184	0.236
Year 2016	−0.078	0.261	−0.300	0.764
Year 2017	−0.595	0.303	−1.967	0.049
Year 2018	−0.280	0.284	−0.986	0.324
Year 2019	−0.326	0.291	−1.121	0.262
Sin	0.044	0.045	0.978	0.328
Cos	−0.179	0.069	−2.576	0.010
**Smooth terms**		**edf**	**Chi square**	**p-value**
s(X, Y)		2.988	258.506	< 0.001
s(Elevation)		2.720	78.830	< 0.001
s(Slope)		2.829	10.599	0.010
s(C110)		1.028	46.390	< 0.001
s(C112)		0.996	42.708	< 0.001
s(C211)		2.117	13.297	< 0.001
s(C213)		0.477	0.982	0.150
s(C221)		0.856	5.460	0.011
s(C222)		0.001	0.001	0.443
s(C223)		0.001	0.001	0.486
s(C224)		2.004	8.385	0.010
s(C231)		0.001	0.000	0.645
s(C311)		2.246	29.157	< 0.001
s(C312)		0.002	0.001	0.428
s(C313)		0.828	3.154	0.050
s(C320)		2.809	29.063	< 0.001
s(C321)		0.001	0.000	0.690
s(C330)		0.001	0.000	0.824
s(C410)		2.880	293.059	< 0.001
s(C511)		2.375	61.413	< 0.001

Melodius Warbler (*Hippolais polyglotta*) — Model C-ZIP-GAM				
**Binomial component**				
**Parametric terms**	**Estimate**	**Standard error**	**z-value**	**p-value**
Intercept	−1.706	0.028	−61.690	< 0.001
**Smooth terms**		**edf**	**Chi square**	**p-value**
s(Elevation)		2.810	434.730	< 0.001
s(Urb_{2500})		1.050	39.040	< 0.001
s(For_{2500})		2.958	278.180	< 0.001
**Count component**				
**Parametric terms**	**Estimate**	**Standard error**	**z-value**	**p-value**
Intercept	−3.588	0.369	−9.721	< 0.001
Year 1995	0.105	0.383	0.274	0.784
Year 1996	−0.958	0.587	−1.633	0.103
Year 1999	0.589	0.347	1.698	0.089
Year 2000	0.101	0.366	0.277	0.782
Year 2001	0.324	0.361	0.895	0.371
Year 2002	0.400	0.385	1.040	0.299
Year 2003	0.459	0.379	1.213	0.225
Year 2004	0.110	0.548	0.202	0.840
Year 2005	0.132	0.368	0.358	0.720
Year 2006	0.460	0.348	1.322	0.186
Year 2007	0.599	0.359	1.671	0.095
Year 2008	0.732	0.383	1.910	0.056
Year 2009	0.855	0.356	2.406	0.016
Year 2010	0.935	0.347	2.696	0.007
Year 2011	0.920	0.343	2.685	0.007
Year 2012	1.151	0.338	3.404	< 0.001
Year 2013	0.965	0.345	2.795	0.005
Year 2014	0.958	0.348	2.756	0.006
Year 2015	1.451	0.343	4.229	< 0.001
Year 2016	0.697	0.364	1.917	0.055
Year 2017	0.785	0.375	2.093	0.036
Year 2018	0.847	0.376	2.249	0.025
Year 2019	1.007	0.359	2.801	0.005
Sin	−0.097	0.062	−1.557	0.120
Cos	0.055	0.098	0.555	0.579
**Smooth terms**		**edf**	**Chi square**	**p-value**
s(X, Y)		1.808	26.327	< 0.001
s(Elevation)		2.124	12.038	0.001
s(Slope)		2.600	5.539	0.086
s(C110)		2.866	20.838	< 0.001
s(C112)		0.973	43.136	< 0.001
s(C211)		2.202	23.420	< 0.001
s(C213)		0.951	18.540	< 0.001
s(C221)		0.895	11.618	< 0.001
s(C222)		0.001	0.000	0.738
s(C223)		0.001	0.001	0.402
s(C224)		1.975	8.857	0.008
s(C231)		0.000	0.000	0.655
s(C311)		2.341	23.101	< 0.001
s(C312)		0.560	1.181	0.145
s(C313)		1.888	4.066	0.105
s(C320)		2.270	92.558	< 0.001
s(C321)		0.000	0.000	0.892
s(C330)		1.867	35.193	< 0.001
s(C410)		1.897	3.395	0.157
s(C511)		1.725	14.918	< 0.001

Lesser Whitethroat (*Curruca curruca*) — Model C-ZIP-GAM				
**Binomial component**				
**Parametric terms**	**Estimate**	**Standard error**	**z-value**	**p-value**
Intercept	−3.533	0.056	−62.690	< 0.001
**Smooth terms**		**edf**	**Chi square**	**p-value**
s(Elevation)		2.992	2907.300	< 0.001
s(Urb_{2500})		0.000	0.000	1.000
s(For_{2500})		2.983	175.800	< 0.001
**Count component**				
**Parametric terms**	**Estimate**	**Standard error**	**z-value**	**p-value**
Intercept	−12.104	33.019	−0.367	0.714
Year 1995	−0.350	0.605	−0.579	0.562
Year 1996	−0.521	0.621	−0.838	0.402
Year 1999	−0.096	0.545	−0.176	0.860
Year 2000	−0.295	0.556	−0.530	0.596
Year 2001	0.185	0.552	0.335	0.738
Year 2002	−1.289	0.771	−1.673	0.094
Year 2003	−0.426	0.576	−0.739	0.460
Year 2004	−0.669	0.635	−1.054	0.292
Year 2005	−0.276	0.546	−0.506	0.613
Year 2006	−0.021	0.568	−0.037	0.970
Year 2007	0.526	0.532	0.989	0.323
Year 2008	−0.110	0.543	−0.203	0.839
Year 2009	−0.147	0.548	−0.269	0.788
Year 2010	0.158	0.538	0.294	0.769
Year 2011	0.332	0.531	0.624	0.533
Year 2012	0.295	0.534	0.553	0.581
Year 2013	0.173	0.534	0.323	0.746
Year 2014	0.282	0.533	0.530	0.596
Year 2015	−0.043	0.535	−0.080	0.936
Year 2016	0.245	0.530	0.461	0.645
Year 2017	0.153	0.540	0.283	0.777
Year 2018	0.069	0.543	0.128	0.899
Year 2019	−0.005	0.544	−0.008	0.993
Sin	−0.254	0.064	−3.939	< 0.001
Cos	−0.117	0.086	−1.355	0.176
**Smooth terms**		**edf**	**Chi square**	**p-value**
s(X, Y)		1.875	26.971	< 0.001
s(Elevation)		0.001	0.000	0.722
s(Slope)		2.194	14.377	< 0.001
s(C110)		1.832	10.298	0.003
s(C112)		0.916	7.685	0.003
s(C211)		0.768	2.913	0.047
s(C213)		0.001	0.000	0.550
s(C221)		0.610	0.355	0.445
s(C222)		0.001	0.000	0.772
s(C223)		0.655	0.048	0.786
s(C224)		0.384	0.066	0.679
s(C231)		1.004	8.518	0.002
s(C311)		0.943	9.521	< 0.001
s(C312)		2.406	33.668	< 0.001
s(C313)		1.808	7.924	0.008
s(C320)		2.357	27.009	< 0.001
s(C321)		2.930	29.635	< 0.001
s(C330)		2.775	21.759	< 0.001
s(C410)		0.007	0.008	0.289
s(C511)		0.760	2.366	0.077

**Eurasian Blackcap (*Sylvia atricapilla*) — Model C-ZIP-GAM**				
**Binomial component**				
**Parametric terms**	**Estimate**	**Standard error**	**z-value**	**p-value**
Intercept	8.224	0.393	20.950	< 0.001
**Smooth terms**		**edf**	**Chi square**	**p-value**
s(Elevation)		2.991	1225.920	< 0.001
s(Urb_{2500})		2.969	160.510	< 0.001
s(For_{2500})		2.549	62.870	< 0.001
**Count component**				
**Parametric terms**	**Estimate**	**Standard error**	**z-value**	**p-value**
Intercept	0.243	0.043	5.587	< 0.001
Year 1995	−0.248	0.056	−4.450	< 0.001
Year 1996	−0.143	0.055	−2.612	0.009
Year 1999	0.001	0.048	0.025	0.980
Year 2000	−0.107	0.048	−2.246	0.025
Year 2001	−0.092	0.051	−1.814	0.070
Year 2002	−0.126	0.056	−2.257	0.024
Year 2003	−0.187	0.058	−3.221	0.001
Year 2004	−0.299	0.068	−4.422	< 0.001
Year 2005	−0.145	0.051	−2.855	0.004
Year 2006	−0.126	0.050	−2.513	0.012
Year 2007	−0.066	0.054	−1.222	0.222
Year 2008	−0.015	0.054	−0.282	0.778
Year 2009	−0.008	0.053	−0.148	0.882
Year 2010	0.035	0.053	0.667	0.505
Year 2011	0.053	0.050	1.046	0.295
Year 2012	−0.015	0.053	−0.286	0.775
Year 2013	−0.102	0.054	−1.892	0.059
Year 2014	−0.103	0.053	−1.945	0.052
Year 2015	−0.111	0.054	−2.066	0.039
Year 2016	−0.120	0.054	−2.233	0.026
Year 2017	0.031	0.056	0.557	0.577
Year 2018	−0.030	0.056	−0.533	0.594
Year 2019	0.012	0.056	0.211	0.833
Sin	−0.010	0.010	−1.050	0.294
Cos	−0.030	0.015	−2.038	0.042
**Smooth terms**		**edf**	**Chi square**	**p-value**
s(X, Y)		2.952	73.506	< 0.001
s(Elevation)		2.577	206.946	< 0.001
s(Slope)		2.815	67.971	< 0.001
s(C110)		2.655	49.698	< 0.001
s(C112)		2.927	56.104	< 0.001
s(C211)		1.754	23.568	< 0.001
s(C213)		0.000	0.000	0.652
s(C221)		2.249	8.264	0.015
s(C222)		0.002	0.000	0.709
s(C223)		2.542	10.151	0.008
s(C224)		1.950	97.983	< 0.001
s(C231)		0.832	7.419	0.002
s(C311)		2.942	513.563	< 0.001
s(C312)		1.808	21.421	< 0.001
s(C313)		2.621	185.940	< 0.001
s(C320)		2.214	49.262	< 0.001
s(C321)		2.873	35.964	< 0.001
s(C330)		0.919	11.937	< 0.001
s(C410)		2.772	5.764	0.100
s(C511)		1.966	23.925	< 0.001

**Western Bonelli's Warbler (*Phylloscopus bonelli*) — Model C-ZINB-GAM**				
**Binomial component**				
**Parametric terms**	**Estimate**	**Standard error**	**z-value**	**p-value**
Intercept	−2.444	0.037	−66.350	< 0.001
**Smooth terms**		**edf**	**Chi square**	**p-value**
s(Elevation)		2.920	359.300	< 0.001
s(Urb_{2500})		1.067	144.900	< 0.001
s(For_{2500})		2.335	114.700	< 0.001
**Count component**				
**Parametric terms**	**Estimate**	**Standard error**	**z-value**	**p-value**
Intercept	−5.220	4.888	−1.068	0.286
Year 1995	1.184	0.351	3.371	< 0.001
Year 1996	0.827	0.363	2.279	0.023
Year 1999	1.285	0.321	4.006	< 0.001
Year 2000	1.041	0.317	3.291	0.001
Year 2001	1.148	0.329	3.486	< 0.001
Year 2002	1.022	0.344	2.966	0.003
Year 2003	0.883	0.350	2.523	0.012
Year 2004	0.647	0.370	1.750	0.080
Year 2005	1.034	0.331	3.121	0.002
Year 2006	0.533	0.361	1.476	0.140
Year 2007	0.901	0.342	2.631	0.009
Year 2008	1.000	0.335	2.984	0.003
Year 2009	0.728	0.353	2.064	0.039
Year 2010	0.977	0.344	2.839	0.005
Year 2011	0.937	0.337	2.781	0.005
Year 2012	1.170	0.333	3.508	< 0.001
Year 2013	1.268	0.332	3.821	< 0.001
Year 2014	1.206	0.338	3.569	< 0.001
Year 2015	1.236	0.338	3.661	< 0.001
Year 2016	1.435	0.330	4.344	< 0.001
Year 2017	1.305	0.339	3.846	< 0.001
Year 2018	1.179	0.345	3.414	< 0.001
Year 2019	1.225	0.343	3.567	< 0.001
Sin	0.190	0.042	4.473	< 0.001
Cos	−0.443	0.067	−6.581	< 0.001
**Smooth terms**		**edf**	**Chi square**	**p-value**
s(X, Y)		2.000	332.708	< 0.001
s(Elevation)		1.091	22.044	< 0.001
s(Slope)		2.827	170.716	< 0.001
s(C110)		0.860	5.078	0.014
s(C112)		0.985	22.914	< 0.001
s(C211)		0.898	5.892	0.009
s(C213)		0.741	0.039	0.818
s(C221)		0.754	2.628	0.061
s(C222)		0.648	1.544	0.122
s(C223)		0.000	0.000	0.545
s(C224)		0.001	0.000	0.506
s(C231)		0.001	0.000	0.378
s(C311)		1.117	23.209	< 0.001
s(C312)		0.002	0.000	0.842
s(C313)		0.262	0.349	0.236
s(C320)		0.937	12.215	< 0.001
s(C321)		0.932	10.762	< 0.001
s(C330)		0.966	23.641	< 0.001
s(C410)		0.000	0.000	0.768
s(C511)		0.973	34.796	< 0.001

Common Chiffchaff (*Phylloscopus collybita*) — Model C-ZIP-GAM				
**Binomial component**				
**Parametric terms**	**Estimate**	**Standard error**	**z-value**	**p-value**
Intercept	−0.679	0.032	−21.230	< 0.001
**Smooth terms**		**edf**	**Chi square**	**p-value**
s(Elevation)		2.990	2239.300	< 0.001
s(Urb_{2500})		0.000	0.000	0.835
s(For_{2500})		2.995	968.400	< 0.001
**Count component**				
**Parametric terms**	**Estimate**	**Standard error**	**z-value**	**p-value**
Intercept	−1.880	0.162	−11.575	< 0.001
Year 1995	0.145	0.145	1.001	0.317
Year 1996	0.527	0.134	3.930	< 0.001
Year 1999	0.077	0.125	0.615	0.538
Year 2000	−0.090	0.123	−0.728	0.466
Year 2001	−0.055	0.132	−0.415	0.678
Year 2002	−0.390	0.145	−2.685	0.007
Year 2003	−0.230	0.142	−1.619	0.105
Year 2004	−0.107	0.151	−0.709	0.478
Year 2005	−0.396	0.139	−2.853	0.004
Year 2006	−0.499	0.147	−3.394	< 0.001
Year 2007	−0.224	0.139	−1.609	0.108
Year 2008	−0.106	0.136	−0.778	0.437
Year 2009	−0.493	0.151	−3.265	0.001
Year 2010	−0.191	0.142	−1.353	0.176
Year 2011	−0.234	0.136	−1.727	0.084
Year 2012	−0.770	0.157	−4.913	< 0.001
Year 2013	−0.572	0.146	−3.907	< 0.001
Year 2014	−0.423	0.144	−2.948	0.003
Year 2015	−0.203	0.137	−1.475	0.140
Year 2016	−0.114	0.134	−0.848	0.397
Year 2017	−0.380	0.148	−2.574	0.010
Year 2018	−0.409	0.149	−2.735	0.006
Year 2019	−0.341	0.148	−2.305	0.021
Sin	0.003	0.024	0.115	0.909
Cos	−0.007	0.035	−0.194	0.846
**Smooth terms**		**edf**	**Chi square**	**p-value**
s(X, Y)		1.938	199.344	< 0.001
s(Elevation)		2.999	187.752	< 0.001
s(Slope)		2.206	42.085	< 0.001
s(C110)		1.020	48.577	< 0.001
s(C112)		0.987	50.352	< 0.001
s(C211)		0.933	12.981	< 0.001
s(C213)		0.863	4.100	0.029
s(C221)		0.780	3.195	0.042
s(C222)		0.842	4.788	0.017
s(C223)		0.796	2.820	0.060
s(C224)		0.003	0.003	0.334
s(C231)		0.000	0.000	0.389
s(C311)		1.976	42.642	< 0.001
s(C312)		2.426	57.767	< 0.001
s(C313)		2.455	75.281	< 0.001
s(C320)		2.065	91.614	< 0.001
s(C321)		2.800	36.050	< 0.001
s(C330)		0.905	9.699	< 0.001
s(C410)		0.671	2.327	0.062
s(C511)		2.836	36.915	< 0.001

Goldcrest (*Regulus regulus*) — Model C-ZIP-GAM				
**Binomial component**				
**Parametric terms**	**Estimate**	**Standard error**	**z-value**	**p-value**
Intercept	−1.747	0.032	−54.360	< 0.001
**Smooth terms**		**edf**	**Chi square**	**p-value**
s(Elevation)		2.993	3335.700	< 0.001
s(Urb_{2500})		0.002	0.000	0.787
s(For_{2500})		2.372	158.900	< 0.001
**Count component**				
**Parametric terms**	**Estimate**	**Standard error**	**z-value**	**p-value**
Intercept	−17.281	50.304	−0.344	0.731
Year 1995	−0.327	0.289	−1.131	0.258
Year 1996	0.480	0.238	2.012	0.044
Year 1999	0.237	0.222	1.070	0.285
Year 2000	0.237	0.217	1.095	0.274
Year 2001	0.187	0.226	0.826	0.409
Year 2002	0.297	0.231	1.283	0.199
Year 2003	−0.025	0.248	−0.103	0.918
Year 2004	−0.539	0.330	−1.635	0.102
Year 2005	−0.266	0.249	−1.069	0.285
Year 2006	−0.297	0.263	−1.130	0.258
Year 2007	−0.011	0.240	−0.046	0.963
Year 2008	−0.139	0.254	−0.546	0.585
Year 2009	−0.794	0.316	−2.513	0.012
Year 2010	−0.147	0.255	−0.577	0.564
Year 2011	−0.248	0.250	−0.994	0.320
Year 2012	0.090	0.246	0.364	0.716
Year 2013	−0.478	0.262	−1.821	0.069
Year 2014	−0.447	0.263	−1.701	0.089
Year 2015	−0.084	0.246	−0.342	0.732
Year 2016	−0.355	0.251	−1.414	0.157
Year 2017	0.183	0.252	0.727	0.468
Year 2018	−0.367	0.291	−1.258	0.208
Year 2019	−0.687	0.322	−2.134	0.033
Sin	0.005	0.040	0.124	0.901
Cos	−0.033	0.055	−0.601	0.548
**Smooth terms**		**edf**	**Chi square**	**p-value**
s(X, Y)		2.970	72.865	< 0.001
s(Elevation)		0.867	4.433	0.019
s(Slope)		2.314	8.217	0.016
s(C110)		2.521	22.495	< 0.001
s(C112)		1.915	5.498	0.047
s(C211)		1.814	3.307	0.160
s(C213)		0.753	0.023	0.861
s(C221)		0.492	0.043	0.767
s(C222)		0.001	0.001	0.432
s(C223)		0.611	0.024	0.843
s(C224)		0.002	0.001	0.503
s(C231)		2.768	8.453	0.025
s(C311)		2.194	6.473	0.036
s(C312)		2.854	155.391	< 0.001
s(C313)		2.290	74.842	< 0.001
s(C320)		0.001	0.000	0.885
s(C321)		1.782	11.781	0.001
s(C330)		0.954	19.631	< 0.001
s(C410)		0.340	0.439	0.256
s(C511)		0.599	1.394	0.127

Common Firecrest (*Regulus ignicapilla*) — Model C-ZIP-GAM				
**Binomial component**				
**Parametric terms**	**Estimate**	**Standard error**	**z-value**	**p-value**
Intercept	−1.497	0.023	−66.390	< 0.001
**Smooth terms**		**edf**	**Chi square**	**p-value**
s(Elevation)		2.992	787.800	< 0.001
s(Urb_{2500})		0.002	0.000	0.918
s(For_{2500})		2.990	551.000	< 0.001
**Count component**				
**Parametric terms**	**Estimate**	**Standard error**	**z-value**	**p-value**
Intercept	−17.761	72.373	−0.245	0.806
Year 1995	0.755	0.416	1.814	0.070
Year 1996	−0.015	0.448	−0.033	0.974
Year 1999	−0.090	0.398	−0.225	0.822
Year 2000	0.442	0.375	1.177	0.239
Year 2001	0.696	0.386	1.803	0.071
Year 2002	0.375	0.400	0.937	0.349
Year 2003	0.041	0.425	0.096	0.924
Year 2004	0.170	0.460	0.370	0.711
Year 2005	0.442	0.400	1.107	0.268
Year 2006	0.179	0.407	0.440	0.660
Year 2007	0.643	0.394	1.633	0.102
Year 2008	0.549	0.399	1.376	0.169
Year 2009	0.246	0.428	0.575	0.566
Year 2010	0.615	0.400	1.537	0.124
Year 2011	0.861	0.383	2.246	0.025
Year 2012	−0.160	0.426	−0.377	0.706
Year 2013	0.874	0.386	2.265	0.023
Year 2014	0.797	0.388	2.053	0.040
Year 2015	0.986	0.381	2.590	0.010
Year 2016	1.161	0.376	3.091	0.002
Year 2017	1.247	0.390	3.198	0.001
Year 2018	1.315	0.392	3.354	< 0.001
Year 2019	1.133	0.398	2.851	0.004
Sin	0.022	0.054	0.413	0.680
Cos	−0.208	0.076	−2.723	0.006
**Smooth terms**		**edf**	**Chi square**	**p-value**
s(X, Y)		2.984	98.478	< 0.001
s(Elevation)		2.811	18.809	< 0.001
s(Slope)		2.990	78.196	< 0.001
s(C110)		0.005	0.011	0.102
s(C112)		0.962	6.689	0.007
s(C211)		1.045	35.230	< 0.001
s(C213)		0.760	0.070	0.762
s(C221)		0.912	6.962	0.006
s(C222)		0.820	3.547	0.037
s(C223)		0.000	0.000	0.570
s(C224)		0.477	0.031	0.797
s(C231)		1.144	52.389	< 0.001
s(C311)		1.142	46.024	< 0.001
s(C312)		2.867	137.639	< 0.001
s(C313)		2.219	20.704	< 0.001
s(C320)		1.045	33.549	< 0.001
s(C321)		1.074	50.962	< 0.001
s(C330)		0.902	5.160	0.016
s(C410)		0.896	5.573	0.013
s(C511)		0.939	12.601	< 0.001

Spotted Flycatcher (*Muscicapa striata*) — Model C-P-GAM				
**Parametric terms**	**Estimate**	**Standard error**	**z-value**	**p-value**
Intercept	−3.096	0.180	−17.207	< 0.001
Year 1995	−0.581	0.249	−2.339	0.019
Year 1996	−0.924	0.272	−3.395	< 0.001
Year 1999	0.407	0.194	2.096	0.036
Year 2000	0.186	0.191	0.972	0.331
Year 2001	0.846	0.192	4.407	< 0.001
Year 2002	0.488	0.211	2.316	0.021
Year 2003	0.125	0.228	0.547	0.585
Year 2004	−0.014	0.266	−0.052	0.958
Year 2005	0.121	0.203	0.596	0.551
Year 2006	0.330	0.197	1.679	0.093
Year 2007	0.487	0.206	2.363	0.018
Year 2008	0.753	0.198	3.808	< 0.001
Year 2009	0.519	0.203	2.549	0.011
Year 2010	0.211	0.211	1.000	0.317
Year 2011	0.682	0.192	3.551	< 0.001
Year 2012	1.026	0.190	5.392	< 0.001
Year 2013	0.912	0.194	4.708	< 0.001
Year 2014	0.877	0.192	4.578	< 0.001
Year 2015	0.705	0.196	3.594	< 0.001
Year 2016	0.759	0.195	3.891	< 0.001
Year 2017	1.137	0.194	5.860	< 0.001
Year 2018	0.808	0.201	4.024	< 0.001
Year 2019	0.931	0.198	4.713	< 0.001
Sin	−0.030	0.030	−0.975	0.330
Cos	−0.033	0.046	−0.722	0.470
**Smooth terms**		**edf**	**Chi square**	**p-value**
s(X,Y)		2.991	302.316	< 0.001
s(Elevation)		2.739	101.454	< 0.001
s(Slope)		2.471	32.920	< 0.001
s(C110)		0.635	2.506	0.032
s(C112)		1.691	12.669	< 0.001
s(C211)		0.992	32.287	< 0.001
s(C213)		0.937	15.935	< 0.001
s(C221)		0.858	9.394	< 0.001
s(C222)		0.018	0.015	0.361
s(C223)		0.917	14.974	< 0.001
s(C224)		0.000	0.000	0.459
s(C231)		2.241	10.438	0.002
s(C311)		2.736	44.717	< 0.001
s(C312)		2.503	12.527	0.002
s(C313)		2.128	11.300	0.001
s(C320)		0.002	0.000	0.603
s(C321)		0.890	11.502	< 0.001
s(C330)		0.806	4.328	0.019
s(C410)		1.774	4.493	0.070
s(C511)		1.839	4.244	0.090

Long-tailed Tit (*Aegithalos caudatus*) — Model C-P-GAM				
**Parametric terms**	**Estimate**	**Standard error**	**z-value**	**p-value**
Intercept	−4.025	0.293	−13.755	< 0.001
Year 1995	0.912	0.321	2.837	0.005
Year 1996	0.359	0.344	1.045	0.296
Year 1999	0.825	0.305	2.707	0.007
Year 2000	0.456	0.307	1.485	0.138
Year 2001	1.233	0.306	4.026	< 0.001
Year 2002	0.974	0.320	3.039	0.002
Year 2003	0.858	0.330	2.600	0.009
Year 2004	0.708	0.360	1.966	0.049
Year 2005	0.614	0.319	1.922	0.055
Year 2006	0.900	0.307	2.928	0.003
Year 2007	1.098	0.317	3.459	< 0.001
Year 2008	1.007	0.321	3.138	0.002
Year 2009	0.683	0.330	2.068	0.039
Year 2010	0.643	0.331	1.941	0.052
Year 2011	1.283	0.307	4.181	< 0.001
Year 2012	0.927	0.321	2.886	0.004
Year 2013	0.947	0.322	2.942	0.003
Year 2014	1.163	0.314	3.706	< 0.001
Year 2015	1.186	0.315	3.767	< 0.001
Year 2016	0.999	0.321	3.117	0.002
Year 2017	1.542	0.313	4.930	< 0.001
Year 2018	0.858	0.334	2.572	0.010
Year 2019	1.020	0.328	3.114	0.002
Sin	−0.084	0.040	−2.109	0.035
Cos	0.004	0.059	0.075	0.940
**Smooth terms**		**edf**	**Chi square**	**p-value**
s(X,Y)		0.003	0.003	0.369
s(Elevation)		2.219	67.388	< 0.001
s(Slope)		2.871	18.680	< 0.001
s(C110)		0.001	0.001	0.453
s(C112)		0.955	17.269	< 0.001
s(C211)		2.215	13.138	< 0.001
s(C213)		0.869	7.351	0.003
s(C221)		0.056	0.059	0.307
s(C222)		0.002	0.001	0.543
s(C223)		0.005	0.001	0.648
s(C224)		0.003	0.002	0.421
s(C231)		1.734	7.360	0.011
s(C311)		2.691	226.157	< 0.001
s(C312)		1.778	35.482	< 0.001
s(C313)		2.039	77.152	< 0.001
s(C320)		0.869	8.040	0.002
s(C321)		0.734	3.286	0.032
s(C330)		0.002	0.000	0.702
s(C410)		0.887	9.246	0.001
s(C511)		0.963	20.142	< 0.001

Marsh Tit (*Poecile palustris*) — Model C-ZIP-GAM				
**Binomial component**				
**Parametric terms**	**Estimate**	**Standard error**	**z-value**	**p-value**
Intercept	−2.118	0.042	−50.010	< 0.001
**Smooth terms**		**edf**	**Chi square**	**p-value**
s(Elevation)		2.984	750.600	< 0.001
s(Urb_{2500})		2.978	538.200	< 0.001
s(For_{2500})		2.985	1006.400	< 0.001
**Count component**				
**Parametric terms**	**Estimate**	**Standard error**	**z-value**	**p-value**
Intercept	−3.897	0.388	−10.031	< 0.001
Year 1995	1.045	0.430	2.429	0.015
Year 1996	0.491	0.459	1.071	0.284
Year 1999	1.040	0.395	2.636	0.008
Year 2000	0.642	0.398	1.614	0.106
Year 2001	1.506	0.400	3.764	< 0.001
Year 2002	1.631	0.398	4.096	< 0.001
Year 2003	1.482	0.407	3.644	< 0.001
Year 2004	1.706	0.412	4.145	< 0.001
Year 2005	1.278	0.406	3.151	0.002
Year 2006	1.386	0.395	3.511	< 0.001
Year 2007	1.677	0.399	4.200	< 0.001
Year 2008	1.420	0.408	3.482	< 0.001
Year 2009	1.826	0.397	4.599	< 0.001
Year 2010	1.848	0.400	4.620	< 0.001
Year 2011	2.331	0.387	6.022	< 0.001
Year 2012	1.760	0.399	4.416	< 0.001
Year 2013	1.730	0.399	4.333	< 0.001
Year 2014	1.927	0.394	4.894	< 0.001
Year 2015	1.558	0.402	3.874	< 0.001
Year 2016	1.644	0.401	4.104	< 0.001
Year 2017	1.439	0.411	3.502	< 0.001
Year 2018	1.554	0.407	3.815	< 0.001
Year 2019	1.951	0.399	4.887	< 0.001
Sin	0.059	0.038	1.536	0.124
Cos	0.234	0.053	4.392	< 0.001
**Smooth terms**		**edf**	**Chi square**	**p-value**
s(X, Y)		2.992	194.740	< 0.001
s(Elevation)		0.423	0.612	0.225
s(Slope)		0.001	0.000	0.509
s(C110)		0.604	2.558	0.035
s(C112)		0.001	0.001	0.396
s(C211)		1.723	7.268	0.012
s(C213)		0.000	0.000	0.501
s(C221)		0.000	0.000	0.879
s(C222)		0.000	0.000	0.871
s(C223)		0.000	0.000	0.634
s(C224)		0.000	0.000	0.670
s(C231)		1.297	13.503	< 0.001
s(C311)		2.415	293.817	< 0.001
s(C312)		1.021	30.814	< 0.001
s(C313)		1.858	266.747	< 0.001
s(C320)		0.362	0.574	0.201
s(C321)		0.000	0.000	0.443
s(C330)		0.770	3.551	0.031
s(C410)		0.000	0.000	0.923
s(C511)		0.000	0.000	0.919

Willow Tit (*Poecile montanus*) — Model C-ZIP-GAM				
**Binomial component**				
**Parametric terms**	**Estimate**	**Standard error**	**z-value**	**p-value**
Intercept	−3.750	0.067	−55.560	< 0.001
**Smooth terms**		**edf**	**Chi square**	**p-value**
s(Elevation)		2.996	1676.880	< 0.001
s(Urb_{2500})		0.927	11.560	< 0.001
s(For_{2500})		2.990	572.420	< 0.001
**Count component**				
**Parametric terms**	**Estimate**	**Standard error**	**z-value**	**p-value**
Intercept	−38.206	53.235	−0.718	0.473
Year 1995	−0.290	0.508	−0.572	0.568
Year 1996	−0.555	0.540	−1.029	0.304
Year 1999	−0.174	0.433	−0.403	0.687
Year 2000	−0.787	0.475	−1.657	0.098
Year 2001	0.691	0.408	1.692	0.091
Year 2002	0.174	0.458	0.380	0.704
Year 2003	0.273	0.421	0.649	0.517
Year 2004	−0.246	0.500	−0.493	0.622
Year 2005	−0.316	0.434	−0.728	0.467
Year 2006	−0.634	0.542	−1.170	0.242
Year 2007	0.112	0.429	0.260	0.795
Year 2008	−0.793	0.491	−1.614	0.107
Year 2009	0.063	0.443	0.141	0.888
Year 2010	0.117	0.432	0.271	0.787
Year 2011	0.328	0.418	0.786	0.432
Year 2012	−0.876	0.491	−1.782	0.075
Year 2013	0.282	0.420	0.671	0.503
Year 2014	−0.318	0.439	−0.724	0.469
Year 2015	−0.091	0.430	−0.211	0.833
Year 2016	−0.142	0.429	−0.331	0.740
Year 2017	−0.168	0.459	−0.367	0.714
Year 2018	−0.081	0.449	−0.181	0.857
Year 2019	0.115	0.447	0.258	0.797
Sin	0.009	0.063	0.149	0.882
Cos	0.044	0.084	0.521	0.602
**Smooth terms**		**edf**	**Chi square**	**p-value**
s(X, Y)		2.829	95.393	< 0.001
s(Elevation)		0.000	0.000	0.463
s(Slope)		0.000	0.000	0.356
s(C110)		0.912	7.866	0.003
s(C112)		0.918	9.441	0.001
s(C211)		0.642	0.437	0.410
s(C213)		0.000	0.000	0.911
s(C221)		0.000	0.000	0.650
s(C222)		0.000	0.000	0.756
s(C223)		0.000	0.000	0.827
s(C224)		0.001	0.000	0.864
s(C231)		1.002	25.088	< 0.001
s(C311)		0.000	0.000	0.666
s(C312)		2.827	60.282	< 0.001
s(C313)		0.000	0.000	0.446
s(C320)		0.000	0.000	0.748
s(C321)		2.401	30.703	< 0.001
s(C330)		0.924	6.796	0.006
s(C410)		0.000	0.000	0.553
s(C511)		0.857	5.673	0.010

European Crested Tit (*Lophophanes cristatus*) — Model C-ZIP-GAM				
**Binomial component**				
**Parametric terms**	**Estimate**	**Standard error**	**z-value**	**p-value**
Intercept	−2.771	0.057	−48.600	< 0.001
**Smooth terms**		**edf**	**Chi square**	**p-value**
s(Elevation)		2.959	1204.730	< 0.001
s(Urb_{2500})		0.943	14.910	< 0.001
s(For_{2500})		2.970	593.070	< 0.001
**Count component**				
**Parametric terms**	**Estimate**	**Standard error**	**z-value**	**p-value**
Intercept	−7.138	8.012	−0.891	0.373
Year 1995	−0.256	0.488	−0.526	0.599
Year 1996	−0.199	0.445	−0.448	0.654
Year 1999	0.924	0.351	2.633	0.008
Year 2000	0.636	0.352	1.809	0.070
Year 2001	−0.332	0.391	−0.850	0.395
Year 2002	0.619	0.366	1.690	0.091
Year 2003	0.465	0.376	1.236	0.217
Year 2004	0.695	0.420	1.656	0.098
Year 2005	0.349	0.372	0.937	0.349
Year 2006	0.369	0.385	0.959	0.338
Year 2007	0.759	0.367	2.069	0.039
Year 2008	0.273	0.386	0.709	0.478
Year 2009	0.804	0.384	2.094	0.036
Year 2010	0.543	0.379	1.434	0.152
Year 2011	0.430	0.377	1.139	0.255
Year 2012	0.210	0.392	0.537	0.591
Year 2013	0.753	0.369	2.043	0.041
Year 2014	0.652	0.371	1.757	0.079
Year 2015	0.361	0.384	0.941	0.347
Year 2016	0.852	0.365	2.332	0.020
Year 2017	0.808	0.385	2.100	0.036
Year 2018	0.868	0.383	2.265	0.023
Year 2019	0.634	0.399	1.589	0.112
Sin	0.075	0.050	1.517	0.129
Cos	−0.003	0.064	−0.040	0.968
**Smooth terms**		**edf**	**Chi square**	**p-value**
s(X, Y)		1.757	15.215	< 0.001
s(Elevation)		0.871	5.537	0.008
s(Slope)		1.099	33.314	< 0.001
s(C110)		1.209	22.095	< 0.001
s(C112)		2.768	38.612	< 0.001
s(C211)		0.948	10.440	< 0.001
s(C213)		0.776	0.030	0.845
s(C221)		0.645	0.116	0.671
s(C222)		0.628	0.787	0.262
s(C223)		0.498	0.021	0.837
s(C224)		0.661	1.053	0.206
s(C231)		0.993	21.683	< 0.001
s(C311)		1.080	48.107	< 0.001
s(C312)		2.913	131.970	< 0.001
s(C313)		0.640	6.136	< 0.001
s(C320)		2.909	31.260	< 0.001
s(C321)		1.707	49.303	< 0.001
s(C330)		0.001	0.000	0.568
s(C410)		0.675	1.590	0.120
s(C511)		0.947	19.296	< 0.001

Coal Tit (*Periparus ater*) — Model C-ZIP-GAM				
**Binomial component**				
**Parametric terms**	**Estimate**	**Standard error**	**z-value**	**p-value**
Intercept	−1.813	0.048	−37.660	< 0.001
**Smooth terms**		**edf**	**Chi square**	**p-value**
s(Elevation)		2.992	2645.119	< 0.001
s(Urb_{2500})		0.784	3.921	0.025
s(For_{2500})		2.977	596.639	< 0.001
**Count component**				
**Parametric terms**	**Estimate**	**Standard error**	**z-value**	**p-value**
Intercept	−2.247	2.547	−0.882	0.378
Year 1995	−0.543	0.138	−3.934	< 0.001
Year 1996	−0.137	0.122	−1.123	0.261
Year 1999	−0.181	0.106	−1.704	0.088
Year 2000	−0.416	0.106	−3.939	< 0.001
Year 2001	−0.112	0.108	−1.037	0.300
Year 2002	−0.214	0.118	−1.817	0.069
Year 2003	−0.227	0.116	−1.954	0.051
Year 2004	−0.250	0.136	−1.835	0.067
Year 2005	−0.568	0.118	−4.831	< 0.001
Year 2006	−0.046	0.115	−0.400	0.689
Year 2007	−0.115	0.112	−1.030	0.303
Year 2008	−0.603	0.125	−4.842	< 0.001
Year 2009	−0.620	0.133	−4.663	< 0.001
Year 2010	−0.310	0.120	−2.585	0.010
Year 2011	−0.221	0.113	−1.955	0.051
Year 2012	−0.329	0.117	−2.805	0.005
Year 2013	−0.232	0.113	−2.058	0.040
Year 2014	−0.362	0.115	−3.139	0.002
Year 2015	−0.449	0.118	−3.815	< 0.001
Year 2016	−0.102	0.110	−0.928	0.354
Year 2017	−0.191	0.122	−1.561	0.118
Year 2018	−0.106	0.122	−0.868	0.386
Year 2019	−0.548	0.133	−4.121	< 0.001
Sin	−0.019	0.020	−0.960	0.337
Cos	0.054	0.028	1.950	0.051
**Smooth terms**		**edf**	**Chi square**	**p-value**
s(X, Y)		1.983	214.084	< 0.001
s(Elevation)		2.527	93.431	< 0.001
s(Slope)		2.387	17.269	< 0.001
s(C110)		2.699	20.897	< 0.001
s(C112)		2.065	22.912	< 0.001
s(C211)		1.023	17.445	< 0.001
s(C213)		0.752	0.121	0.689
s(C221)		0.904	8.814	0.002
s(C222)		0.872	5.747	0.010
s(C223)		0.784	1.818	0.128
s(C224)		0.860	4.790	0.018
s(C231)		1.562	20.596	< 0.001
s(C311)		2.735	25.661	< 0.001
s(C312)		2.956	281.594	< 0.001
s(C313)		2.513	98.128	< 0.001
s(C320)		2.842	27.074	< 0.001
s(C321)		2.648	65.987	< 0.001
s(C330)		2.848	10.926	0.009
s(C410)		0.887	5.414	0.013
s(C511)		0.980	23.867	< 0.001

**Eurasian Blue Tit (*Cyanistes caeruleus*) — Model C-ZIP-GAM**				
**Binomial component**				
**Parametric terms**	**Estimate**	**Standard error**	**z-value**	**p-value**
Intercept	−0.432	0.022	−19.920	< 0.001
**Smooth terms**		**edf**	**Chi square**	**p-value**
s(Elevation)		2.796	1698.100	< 0.001
s(Urb_{2500})		2.943	306.300	< 0.001
s(For_{2500})		2.962	1448.100	< 0.001
**Count component**				
**Parametric terms**	**Estimate**	**Standard error**	**z-value**	**p-value**
Intercept	−2.293	0.166	−13.834	< 0.001
Year 1995	−0.026	0.214	−0.122	0.903
Year 1996	0.584	0.188	3.105	0.002
Year 1999	0.352	0.174	2.017	0.044
Year 2000	0.534	0.170	3.142	0.002
Year 2001	0.869	0.176	4.937	< 0.001
Year 2002	0.608	0.184	3.306	< 0.001
Year 2003	0.937	0.182	5.144	< 0.001
Year 2004	0.329	0.220	1.495	0.135
Year 2005	0.478	0.183	2.614	0.009
Year 2006	0.599	0.175	3.423	< 0.001
Year 2007	0.620	0.184	3.374	< 0.001
Year 2008	0.830	0.181	4.580	< 0.001
Year 2009	0.696	0.184	3.791	< 0.001
Year 2010	0.819	0.182	4.500	< 0.001
Year 2011	0.992	0.172	5.753	< 0.001
Year 2012	0.717	0.181	3.962	< 0.001
Year 2013	0.621	0.182	3.406	< 0.001
Year 2014	0.716	0.180	3.973	< 0.001
Year 2015	0.450	0.189	2.385	0.017
Year 2016	0.572	0.186	3.071	0.002
Year 2017	0.654	0.188	3.468	< 0.001
Year 2018	0.418	0.196	2.136	0.033
Year 2019	0.423	0.196	2.154	0.031
Sin	−0.052	0.026	−1.996	0.046
Cos	0.093	0.039	2.387	0.017
**Smooth terms**		**edf**	**Chi square**	**p-value**
s(X, Y)		2.984	155.942	< 0.001
s(Elevation)		2.372	48.588	< 0.001
S(Slope)		1.856	4.496	0.071
s(C110)		0.979	17.841	< 0.001
s(C112)		2.833	10.779	0.009
s(C211)		2.241	30.049	< 0.001
s(C213)		0.980	19.241	< 0.001
s(C221)		2.410	16.012	< 0.001
s(C222)		0.793	6.065	0.005
s(C223)		0.553	1.106	0.156
s(C224)		1.864	13.713	< 0.001
s(C231)		1.765	4.958	0.041
s(C311)		2.608	69.971	< 0.001
s(C312)		1.867	8.433	0.007
s(C313)		2.221	30.566	< 0.001
s(C320)		2.501	2.874	0.314
s(C321)		1.414	9.298	0.003
s(C330)		0.723	2.752	0.048
s(C410)		0.770	4.851	0.011
s(C511)		0.000	0.000	0.902

Great Tit (*Parus major*) — Model C-ZIP-GAM				
**Binomial component**				
**Parametric terms**	**Estimate**	**Standard error**	**z-value**	**p-value**
Intercept	3.033	0.061	49.620	< 0.001
**Smooth terms**		**edf**	**Chi square**	**p-value**
s(Elevation)		2.941	1262.800	< 0.001
s(Urb_{2500})		2.984	332.000	< 0.001
s(For_{2500})		2.996	829.100	< 0.001
**Count component**				
**Parametric terms**	**Estimate**	**Standard error**	**z-value**	**p-value**
Intercept	−0.625	0.069	−9.079	< 0.001
Year 1995	−0.617	0.092	−6.679	< 0.001
Year 1996	−0.707	0.094	−7.500	< 0.001
Year 1999	−0.168	0.075	−2.225	0.026
Year 2000	−0.512	0.077	−6.661	< 0.001
Year 2001	−0.280	0.080	−3.509	< 0.001
Year 2002	−0.437	0.092	−4.755	< 0.001
Year 2003	−0.021	0.086	−0.244	0.807
Year 2004	−0.259	0.102	−2.542	0.011
Year 2005	−0.194	0.078	−2.483	0.013
Year 2006	−0.152	0.076	−2.007	0.045
Year 2007	−0.075	0.082	−0.912	0.362
Year 2008	−0.128	0.084	−1.524	0.128
Year 2009	−0.027	0.081	−0.332	0.740
Year 2010	0.074	0.079	0.941	0.346
Year 2011	−0.055	0.078	−0.707	0.480
Year 2012	−0.060	0.081	−0.738	0.460
Year 2013	0.022	0.079	0.280	0.779
Year 2014	−0.093	0.080	−1.172	0.241
Year 2015	−0.015	0.080	−0.185	0.853
Year 2016	0.049	0.079	0.612	0.541
Year 2017	0.109	0.083	1.321	0.186
Year 2018	0.124	0.082	1.511	0.131
Year 2019	0.112	0.082	1.363	0.173
Sin	0.012	0.015	0.807	0.420
Cos	0.015	0.023	0.665	0.506
**Smooth terms**		**edf**	**Chi square**	**p-value**
s(X, Y)		2.873	71.283	< 0.001
s(Elevation)		2.934	202.019	< 0.001
s(Slope)		2.356	13.474	< 0.001
s(C110)		1.336	4.231	0.036
s(C112)		2.223	12.596	0.001
s(C211)		0.897	5.497	0.007
s(C213)		0.998	23.605	< 0.001
s(C221)		0.244	0.450	0.164
s(C222)		0.002	0.001	0.381
s(C223)		0.001	0.001	0.427
s(C224)		2.627	37.439	< 0.001
s(C231)		2.748	21.450	< 0.001
s(C311)		2.853	204.343	< 0.001
s(C312)		1.365	13.620	< 0.001
s(C313)		2.285	48.279	< 0.001
s(C320)		0.002	0.001	0.477
s(C321)		1.999	4.977	0.070
s(C330)		0.877	4.508	0.022
s(C410)		0.860	4.359	0.023
s(C511)		2.699	13.864	0.002

Eurasian Nuthatch (*Sitta europaea*) — Model C-ZIP-GAM				
**Binomial component**				
**Parametric terms**	**Estimate**	**Standard error**	**z-value**	**p-value**
Intercept	−2.286	0.045	−50.830	< 0.001
**Smooth terms**		**edf**	**Chi square**	**p-value**
s(Elevation)		2.995	604.100	< 0.001
s(Urb_{2500})		2.996	592.900	< 0.001
s(For_{2500})		2.993	888.300	< 0.001
**Count component**				
**Parametric terms**	**Estimate**	**Standard error**	**z-value**	**p-value**
Intercept	−2.747	0.271	−10.154	< 0.001
Year 1995	−0.299	0.350	−0.853	0.393
Year 1996	−1.232	0.434	−2.838	0.005
Year 1999	0.240	0.272	0.883	0.377
Year 2000	−0.144	0.276	−0.522	0.601
Year 2001	0.328	0.285	1.151	0.250
Year 2002	0.292	0.290	1.008	0.313
Year 2003	−0.079	0.333	−0.236	0.813
Year 2004	−1.285	0.481	−2.670	0.008
Year 2005	−0.038	0.306	−0.123	0.902
Year 2006	0.423	0.280	1.510	0.131
Year 2007	−0.097	0.319	−0.303	0.762
Year 2008	−0.146	0.327	−0.446	0.656
Year 2009	0.070	0.307	0.229	0.819
Year 2010	−0.248	0.344	−0.720	0.471
Year 2011	0.253	0.293	0.863	0.388
Year 2012	0.372	0.295	1.264	0.206
Year 2013	−0.291	0.340	−0.857	0.391
Year 2014	0.260	0.296	0.879	0.380
Year 2015	−0.081	0.317	−0.256	0.798
Year 2016	−0.292	0.344	−0.848	0.397
Year 2017	0.154	0.324	0.474	0.635
Year 2018	−0.392	0.367	−1.070	0.285
Year 2019	0.253	0.317	0.799	0.424
Sin	0.016	0.050	0.325	0.745
Cos	0.295	0.070	4.241	< 0.001
**Smooth terms**		**edf**	**Chi square**	**p-value**
s(X, Y)		2.992	130.001	< 0.001
s(Elevation)		2.882	29.477	< 0.001
s(Slope)		0.755	2.916	0.045
s(C110)		2.624	12.085	0.003
s(C112)		0.003	0.001	0.648
s(C211)		0.000	0.000	0.962
s(C213)		0.000	0.000	0.540
s(C221)		0.002	0.001	0.385
s(C222)		0.002	0.001	0.387
s(C223)		0.000	0.000	0.515
s(C224)		0.000	0.000	0.439
s(C231)		0.716	2.434	0.062
s(C311)		2.498	218.444	< 0.001
s(C312)		1.075	31.987	< 0.001
s(C313)		1.463	137.484	< 0.001
s(C320)		0.000	0.000	1.000
s(C321)		0.000	0.000	0.952
s(C330)		2.843	19.925	< 0.001
s(C410)		0.757	2.363	0.077
s(C511)		2.902	41.362	< 0.001

**Short-toed Treecreeper (*Certhia brachydactyla*) — Model C-ZIP-GAM**				
**Binomial component**				
**Parametric terms**	**Estimate**	**Standard error**	**z-value**	**p-value**
Intercept	−2.663	0.049	−53.940	< 0.001
**Smooth terms**		**edf**	**Chi square**	**p-value**
s(Elevation)		2.988	1005.700	< 0.001
s(Urb_{2500})		1.114	232.800	< 0.001
s(For_{2500})		2.973	741.600	< 0.001
**Count component**				
**Parametric terms**	**Estimate**	**Standard error**	**z-value**	**p-value**
Intercept	−6.025	1.011	−5.961	< 0.001
Year 1995	0.724	1.157	0.626	0.531
Year 1996	2.033	1.016	2.001	0.045
Year 1999	1.758	1.021	1.722	0.085
Year 2000	1.822	1.051	1.734	0.083
Year 2001	2.707	1.019	2.657	0.008
Year 2002	2.983	1.020	2.924	0.003
Year 2003	2.684	1.043	2.573	0.010
Year 2004	2.202	1.034	2.129	0.033
Year 2005	2.980	1.008	2.956	0.003
Year 2006	2.483	1.031	2.409	0.016
Year 2007	2.741	1.028	2.666	0.008
Year 2008	1.664	1.081	1.539	0.124
Year 2009	2.293	1.046	2.194	0.028
Year 2010	2.883	1.019	2.828	0.005
Year 2011	2.965	1.021	2.904	0.004
Year 2012	3.042	1.023	2.973	0.003
Year 2013	2.937	1.021	2.877	0.004
Year 2014	2.571	1.036	2.481	0.013
Year 2015	2.937	1.025	2.866	0.004
Year 2016	3.004	1.027	2.925	0.003
Year 2017	3.236	1.022	3.167	0.002
Year 2018	3.180	1.022	3.112	0.002
Year 2019	0.065	0.069	0.941	0.347
Sin	0.135	0.102	1.319	0.187
Cos	−6.025	1.011	−5.961	< 0.001
**Smooth terms**		**edf**	**Chi square**	**p-value**
s(X, Y)		2.870	114.120	< 0.001
s(Elevation)		0.000	0.000	0.871
s(Slope)		2.132	22.630	< 0.001
s(C110)		1.685	13.536	< 0.001
s(C112)		1.981	11.735	0.001
s(C211)		0.000	0.000	0.739
s(C213)		0.001	0.000	0.401
s(C221)		0.840	4.347	0.023
s(C222)		0.182	0.238	0.251
s(C223)		0.000	0.000	0.612
s(C224)		0.000	0.000	0.622
s(C231)		0.635	1.946	0.074
s(C311)		2.613	82.052	< 0.001
s(C312)		1.752	20.578	< 0.001
s(C313)		2.098	101.850	< 0.001
s(C320)		0.125	0.141	0.281
s(C321)		0.000	0.000	0.800
s(C330)		0.574	1.185	0.149
s(C410)		0.187	0.234	0.261
s(C511)		0.866	7.435	0.003

Eurasian Golden Oriole (*Oriolus oriolus*) — Model C-ZIP-GAM				
**Binomial component**				
**Parametric terms**	**Estimate**	**Standard error**	**z-value**	**p-value**
Intercept	−0.683	0.022	−30.830	< 0.001
**Smooth terms**		**edf**	**Chi square**	**p-value**
s(Elevation)		2.949	2012.600	< 0.001
s(Urb_{2500})		1.190	1228.600	< 0.001
s(For_{2500})		2.994	818.900	< 0.001
**Count component**				
**Parametric terms**	**Estimate**	**Standard error**	**z-value**	**p-value**
Intercept	−2.696	0.205	−13.138	< 0.001
Year 1995	−0.404	0.169	−2.390	0.017
Year 1996	−0.880	0.204	−4.320	< 0.001
Year 1999	−0.557	0.154	−3.617	< 0.001
Year 2000	−0.443	0.159	−2.794	0.005
Year 2001	−0.650	0.176	−3.699	< 0.001
Year 2002	−0.374	0.185	−2.021	0.043
Year 2003	0.066	0.168	0.392	0.695
Year 2004	−0.022	0.236	−0.095	0.924
Year 2005	−0.078	0.152	−0.513	0.608
Year 2006	−0.496	0.166	−2.991	0.003
Year 2007	0.057	0.155	0.366	0.714
Year 2008	−0.078	0.170	−0.460	0.646
Year 2009	−0.174	0.169	−1.026	0.305
Year 2010	−0.076	0.165	−0.458	0.647
Year 2011	−0.348	0.166	−2.099	0.036
Year 2012	−0.120	0.164	−0.733	0.464
Year 2013	0.099	0.152	0.653	0.514
Year 2014	0.101	0.157	0.643	0.520
Year 2015	0.077	0.161	0.478	0.633
Year 2016	0.031	0.161	0.195	0.846
Year 2017	0.162	0.165	0.980	0.327
Year 2018	0.027	0.172	0.159	0.873
Year 2019	0.133	0.166	0.798	0.425
Sin	−0.016	0.034	−0.456	0.648
Cos	0.063	0.052	1.223	0.221
**Smooth terms**		**edf**	**Chi square**	**p-value**
s(X, Y)		2.937	188.880	< 0.001
s(Elevation)		1.877	8.298	0.009
s(Slope)		2.375	39.808	< 0.001
s(C110)		2.886	60.827	< 0.001
s(C112)		1.752	41.716	< 0.001
s(C211)		2.505	72.325	< 0.001
s(C213)		0.778	5.226	0.009
s(C221)		0.001	0.000	0.840
s(C222)		0.790	3.211	0.044
s(C223)		0.001	0.000	0.585
s(C224)		2.944	276.559	< 0.001
s(C231)		0.001	0.000	0.729
s(C311)		2.634	145.798	< 0.001
s(C312)		0.001	0.000	0.934
s(C313)		0.001	0.000	0.814
s(C320)		0.548	1.291	0.124
s(C321)		0.000	0.000	0.870
s(C330)		1.848	8.288	0.010
s(C410)		0.006	0.005	0.358
s(C511)		0.003	0.002	0.342

Red-backed Shrike (*Lanius collurio*) — Model C-ZIP-GAM				
**Binomial component**				
**Parametric terms**	**Estimate**	**Standard error**	**z-value**	**p-value**
Intercept	−0.863	0.020	−42.370	< 0.001
**Smooth terms**		**edf**	**Chi square**	**p-value**
s(Elevation)		2.985	1121.200	< 0.001
s(Urb_{2500})		1.558	175.900	< 0.001
s(For_{2500})		2.970	392.100	< 0.001
**Count component**				
**Parametric terms**	**Estimate**	**Standard error**	**z-value**	**p-value**
Intercept	−1.488	0.153	−9.757	< 0.001
Year 1995	−0.072	0.183	−0.392	0.695
Year 1996	−0.701	0.203	−3.462	< 0.001
Year 1999	−0.576	0.180	−3.194	0.001
Year 2000	−0.681	0.176	−3.873	< 0.001
Year 2001	−0.387	0.180	−2.153	0.031
Year 2002	−0.881	0.227	−3.889	< 0.001
Year 2003	−1.922	0.363	−5.299	< 0.001
Year 2004	−1.233	0.334	−3.694	< 0.001
Year 2005	−0.843	0.196	−4.299	< 0.001
Year 2006	−1.119	0.212	−5.275	< 0.001
Year 2007	−0.680	0.201	−3.388	< 0.001
Year 2008	−0.917	0.221	−4.141	< 0.001
Year 2009	−1.187	0.244	−4.866	< 0.001
Year 2010	−0.987	0.223	−4.422	< 0.001
Year 2011	−1.608	0.251	−6.413	< 0.001
Year 2012	−1.859	0.287	−6.484	< 0.001
Year 2013	−1.161	0.230	−5.044	< 0.001
Year 2014	−1.323	0.248	−5.338	< 0.001
Year 2015	−1.274	0.244	−5.219	< 0.001
Year 2016	−1.091	0.222	−4.911	< 0.001
Year 2017	−1.632	0.294	−5.548	< 0.001
Year 2018	−1.739	0.311	−5.594	< 0.001
Year 2019	−2.025	0.346	−5.852	< 0.001
Sin	−0.055	0.051	−1.088	0.277
Cos	−0.132	0.079	−1.682	0.093
**Smooth terms**		**edf**	**Chi square**	**p-value**
s(X, Y)		1.931	52.891	< 0.001
s(Elevation)		2.320	10.213	0.004
s(Slope)		2.078	24.021	< 0.001
s(C110)		1.506	31.453	< 0.001
s(C112)		1.758	28.458	< 0.001
s(C211)		0.000	0.000	0.866
s(C213)		0.529	1.463	0.095
s(C221)		0.795	5.718	0.007
s(C222)		0.001	0.000	0.481
s(C223)		0.002	0.000	0.912
s(C224)		1.974	5.889	0.041
s(C231)		0.990	59.606	< 0.001
s(C311)		2.344	73.707	< 0.001
s(C312)		1.379	39.741	< 0.001
s(C313)		1.977	34.830	< 0.001
s(C320)		2.494	21.804	< 0.001
s(C321)		0.008	0.007	0.348
s(C330)		0.881	7.668	0.003
s(C410)		0.730	2.397	0.070
s(C511)		0.108	0.123	0.283

Eurasian Jay (*Garrulus glandarius*) — Model C-P-GAM				
**Parametric terms**	**Estimate**	**Standard error**	**z-value**	**p-value**
Intercept	−3.949	0.235	−16.772	< 0.001
Year 1995	0.318	0.293	1.086	0.277
Year 1996	−0.303	0.315	−0.964	0.335
Year 1999	0.458	0.251	1.821	0.069
Year 2000	0.532	0.245	2.171	0.030
Year 2001	0.464	0.260	1.786	0.074
Year 2002	0.727	0.259	2.812	0.005
Year 2003	0.931	0.263	3.533	< 0.001
Year 2004	0.659	0.293	2.248	0.025
Year 2005	0.642	0.257	2.503	0.012
Year 2006	0.816	0.251	3.248	0.001
Year 2007	0.854	0.255	3.349	< 0.001
Year 2008	0.588	0.269	2.186	0.029
Year 2009	0.800	0.260	3.074	0.002
Year 2010	0.614	0.266	2.303	0.021
Year 2011	1.013	0.250	4.057	< 0.001
Year 2012	0.871	0.256	3.409	< 0.001
Year 2013	1.096	0.250	4.378	< 0.001
Year 2014	0.925	0.255	3.631	< 0.001
Year 2015	0.781	0.260	3.003	0.003
Year 2016	1.167	0.251	4.647	< 0.001
Year 2017	1.014	0.261	3.893	< 0.001
Year 2018	0.946	0.263	3.594	< 0.001
Year 2019	0.847	0.266	3.178	0.001
Sin	0.068	0.035	1.965	0.049
Cos	−0.072	0.051	−1.400	0.161
**Smooth terms**		**edf**	**Chi square**	**p-value**
s(X,Y)		2.999	422.098	< 0.001
s(Elevation)		2.692	161.667	< 0.001
s(Slope)		2.597	40.981	< 0.001
s(C110)		0.865	12.151	< 0.001
s(C112)		0.767	3.796	0.020
s(C211)		2.006	6.691	0.017
s(C213)		0.887	12.070	< 0.001
s(C221)		0.911	9.764	< 0.001
s(C222)		0.586	1.602	0.094
s(C223)		0.003	0.002	0.421
s(C224)		2.837	18.247	< 0.001
s(C231)		0.919	24.467	< 0.001
s(C311)		2.735	77.426	< 0.001
s(C312)		1.831	69.811	< 0.001
s(C313)		2.255	62.657	< 0.001
s(C320)		2.618	6.387	0.056
s(C321)		0.002	0.001	0.494
s(C330)		0.879	8.041	0.002
s(C410)		0.256	0.348	0.237
s(C511)		2.896	19.981	< 0.001

**Eurasian Magpie (*Pica pica*) — Model C-ZIP-GAM**				
**Binomial component**				
**Parametric terms**	**Estimate**	**Standard error**	**z-value**	**p-value**
Intercept	0.130	0.022	5.935	< 0.001
**Smooth terms**		**edf**	**Chi square**	**p-value**
s(Elevation)		2.832	1228.500	< 0.001
s(Urb_{2500})		2.991	427.300	< 0.001
s(For_{2500})		2.985	496.000	< 0.001
**Count component**				
**Parametric terms**	**Estimate**	**Standard error**	**z-value**	**p-value**
Intercept	−4.327	0.317	−13.663	< 0.001
Year 1995	0.851	0.273	3.121	0.002
Year 1996	0.280	0.280	1.000	0.317
Year 1999	0.658	0.283	2.321	0.020
Year 2000	0.687	0.270	2.550	0.011
Year 2001	0.675	0.270	2.500	0.012
Year 2002	1.194	0.266	4.483	< 0.001
Year 2003	1.203	0.265	4.532	< 0.001
Year 2004	0.848	0.297	2.856	0.004
Year 2005	1.348	0.253	5.332	< 0.001
Year 2006	1.366	0.255	5.359	< 0.001
Year 2007	0.919	0.267	3.441	< 0.001
Year 2008	1.069	0.265	4.034	< 0.001
Year 2009	1.275	0.259	4.928	< 0.001
Year 2010	1.321	0.259	5.106	< 0.001
Year 2011	1.271	0.257	4.949	< 0.001
Year 2012	1.600	0.255	6.278	< 0.001
Year 2013	1.687	0.252	6.687	< 0.001
Year 2014	1.345	0.256	5.254	< 0.001
Year 2015	1.490	0.255	5.850	< 0.001
Year 2016	1.777	0.252	7.042	< 0.001
Year 2017	1.821	0.257	7.083	< 0.001
Year 2018	2.158	0.254	8.488	< 0.001
Year 2019	1.923	0.256	7.508	< 0.001
Sin	−0.027	0.029	−0.929	0.353
Cos	0.092	0.041	2.260	0.024
**Smooth terms**		**edf**	**Chi square**	**p-value**
s(X, Y)		2.989	538.714	< 0.001
s(Elevation)		2.740	23.190	< 0.001
s(Slope)		2.253	49.829	< 0.001
s(C110)		2.879	98.385	< 0.001
s(C112)		1.000	17.180	< 0.001
s(C211)		2.693	26.948	< 0.001
s(C213)		0.968	27.905	< 0.001
s(C221)		1.667	6.188	0.023
s(C222)		0.897	7.571	0.004
s(C223)		0.896	18.516	< 0.001
s(C224)		2.936	43.789	< 0.001
s(C231)		2.342	5.083	0.077
s(C311)		2.063	14.276	< 0.001
s(C312)		2.104	18.278	< 0.001
s(C313)		0.000	0.000	0.200
s(C320)		0.001	0.000	0.549
s(C321)		0.685	2.502	0.054
s(C330)		0.710	2.785	0.045
s(C410)		0.291	0.593	0.139
s(C511)		1.985	51.380	< 0.001

**Carrion Crow (*Corvus corone*) — Model C-ZIP-GAM**				
**Binomial component**				
**Parametric terms**	**Estimate**	**Standard error**	**z-value**	**p-value**
Intercept	−2.863	0.053	−54.160	< 0.001
**Smooth terms**		**edf**	**Chi square**	**p-value**
s(Elevation)		2.984	1431.700	< 0.001
s(Urb_{2500})		2.994	376.100	< 0.001
s(For_{2500})		2.996	542.100	< 0.001
**Count component**				
**Parametric terms**	**Estimate**	**Standard error**	**z-value**	**p-value**
Intercept	−5.174	0.647	−7.995	< 0.001
Year 1995	−0.692	0.767	−0.903	0.367
Year 1996	0.181	0.576	0.315	0.753
Year 1999	0.282	0.532	0.530	0.596
Year 2000	0.667	0.525	1.272	0.203
Year 2001	0.632	0.522	1.211	0.226
Year 2002	0.041	0.604	0.069	0.945
Year 2003	−0.474	0.589	−0.805	0.421
Year 2004	−0.366	0.636	−0.576	0.565
Year 2005	0.373	0.526	0.709	0.478
Year 2006	1.170	0.535	2.189	0.029
Year 2007	0.229	0.543	0.422	0.673
Year 2008	0.079	0.550	0.144	0.886
Year 2009	0.464	0.535	0.867	0.386
Year 2010	0.672	0.528	1.271	0.204
Year 2011	−0.285	0.557	−0.512	0.609
Year 2012	0.326	0.532	0.612	0.540
Year 2013	0.407	0.529	0.769	0.442
Year 2014	−0.004	0.537	−0.008	0.994
Year 2015	0.057	0.530	0.108	0.914
Year 2016	−0.086	0.542	−0.159	0.873
Year 2017	−0.610	0.596	−1.024	0.306
Year 2018	−0.033	0.564	−0.059	0.953
Year 2019	0.041	0.553	0.074	0.941
Sin	0.018	0.059	0.309	0.757
Cos	0.150	0.079	1.904	0.057
**Smooth terms**		**edf**	**Chi square**	**p-value**
s(X, Y)		2.934	259.492	< 0.001
s(Elevation)		2.890	160.879	< 0.001
s(Slope)		1.688	8.534	0.006
s(C110)		0.000	0.000	0.568
s(C112)		1.859	6.212	0.027
s(C211)		0.000	0.000	0.883
s(C213)		0.000	0.000	0.918
s(C221)		0.006	0.006	0.314
s(C222)		0.751	1.916	0.110
s(C223)		0.001	0.000	0.440
s(C224)		0.000	0.000	0.602
s(C231)		1.749	41.659	< 0.001
s(C311)		0.000	0.000	0.924
s(C312)		2.810	27.190	< 0.001
s(C313)		0.497	1.046	0.139
s(C320)		0.550	2.109	0.040
s(C321)		0.737	7.744	< 0.001
s(C330)		1.684	2.559	0.200
s(C410)		0.000	0.000	0.828
s(C511)		0.000	0.000	0.581

**Hooded Crow (*Corvus cornix*) — Model C-ZINB-GAM**				
**Binomial component**				
**Parametric terms**	**Estimate**	**Standard error**	**z-value**	**p-value**
Intercept	5.315	0.158	33.630	< 0.001
**Smooth terms**		**edf**	**Chi square**	**p-value**
s(Elevation)		1.457	1456.400	< 0.001
s(Urb_{2500})		0.001	0.000	0.910
s(For_{2500})		2.980	661.300	< 0.001
**Count component**				
**Parametric terms**	**Estimate**	**Standard error**	**z-value**	**p-value**
Intercept	−0.088	0.062	−1.420	0.156
Year 1995	−0.471	0.080	−5.924	< 0.001
Year 1996	−0.475	0.078	−6.081	< 0.001
Year 1999	−0.016	0.071	−0.221	0.825
Year 2000	−0.076	0.069	−1.099	0.272
Year 2001	−0.061	0.071	−0.860	0.390
Year 2002	0.020	0.078	0.251	0.802
Year 2003	−0.231	0.083	−2.779	0.005
Year 2004	−0.123	0.091	−1.345	0.179
Year 2005	−0.092	0.071	−1.302	0.193
Year 2006	0.039	0.070	0.554	0.579
Year 2007	−0.161	0.076	−2.106	0.035
Year 2008	−0.080	0.076	−1.050	0.294
Year 2009	−0.249	0.076	−3.280	0.001
Year 2010	−0.098	0.075	−1.314	0.189
Year 2011	0.042	0.072	0.575	0.565
Year 2012	−0.041	0.075	−0.542	0.588
Year 2013	0.001	0.074	0.009	0.992
Year 2014	0.026	0.073	0.361	0.718
Year 2015	−0.057	0.075	−0.757	0.449
Year 2016	−0.038	0.075	−0.504	0.615
Year 2017	−0.075	0.082	−0.917	0.359
Year 2018	−0.106	0.081	−1.309	0.191
Year 2019	−0.162	0.082	−1.979	0.048
Sin	0.064	0.014	4.471	< 0.001
Cos	0.004	0.022	0.190	0.850
**Smooth terms**		**edf**	**Chi square**	**p-value**
s(X, Y)		1.979	241.191	< 0.001
s(Elevation)		1.864	138.533	< 0.001
s(Slope)		2.505	42.907	< 0.001
s(C110)		1.934	36.587	< 0.001
s(C112)		1.061	34.312	< 0.001
s(C211)		1.861	36.774	< 0.001
s(C213)		2.669	225.123	< 0.001
s(C221)		0.822	4.528	0.017
s(C222)		0.515	1.050	0.151
s(C223)		0.640	1.698	0.103
s(C224)		2.389	93.992	< 0.001
s(C231)		2.691	98.033	< 0.001
s(C311)		2.837	117.467	< 0.001
s(C312)		2.080	16.599	< 0.001
s(C313)		0.967	12.294	< 0.001
s(C320)		0.985	25.176	< 0.001
s(C321)		0.806	4.028	0.022
s(C330)		0.915	10.360	< 0.001
s(C410)		0.001	0.000	0.597
s(C511)		0.945	16.368	< 0.001

**Common Starling (*Sturnus vulgaris*) — Model C-ZINB-GAM**				
**Binomial component**				
**Parametric terms**	**Estimate**	**Standard error**	**z-value**	**p-value**
Intercept	4.055	0.115	35.330	< 0.001
**Smooth terms**		**edf**	**Chi square**	**p-value**
s(Elevation)		2.933	1429.140	< 0.001
s(Urb_{2500})		0.999	77.630	< 0.001
s(For_{2500})		1.495	561.930	< 0.001
**Count component**				
**Parametric terms**	**Estimate**	**Standard error**	**z-value**	**p-value**
Intercept	−1.444	0.180	−8.023	< 0.001
Year 1995	−0.441	0.083	−5.316	< 0.001
Year 1996	−0.876	0.086	−10.163	< 0.001
Year 1999	−0.117	0.078	−1.502	0.133
Year 2000	−0.127	0.074	−1.717	0.086
Year 2001	−0.510	0.078	−6.519	< 0.001
Year 2002	−0.367	0.087	−4.210	< 0.001
Year 2003	−0.580	0.091	−6.363	< 0.001
Year 2004	−0.473	0.103	−4.607	< 0.001
Year 2005	−0.305	0.074	−4.114	< 0.001
Year 2006	−0.309	0.075	−4.112	< 0.001
Year 2007	−0.412	0.081	−5.087	< 0.001
Year 2008	−0.331	0.081	−4.063	< 0.001
Year 2009	−0.480	0.081	−5.915	< 0.001
Year 2010	−0.360	0.080	−4.505	< 0.001
Year 2011	−0.456	0.079	−5.801	< 0.001
Year 2012	−0.340	0.080	−4.254	< 0.001
Year 2013	−0.509	0.080	−6.350	< 0.001
Year 2014	−0.332	0.077	−4.282	< 0.001
Year 2015	−0.577	0.082	−7.067	< 0.001
Year 2016	−0.380	0.080	−4.741	< 0.001
Year 2017	−0.694	0.094	−7.409	< 0.001
Year 2018	−0.609	0.092	−6.616	< 0.001
Year 2019	−0.742	0.094	−7.868	< 0.001
Sin	0.012	0.017	0.692	0.489
Cos	0.004	0.025	0.142	0.887
**Smooth terms**		**edf**	**Chi square**	**p-value**
s(X, Y)		2.030	334.840	< 0.001
s(Elevation)		2.659	69.480	< 0.001
s(Slope)		1.671	176.691	< 0.001
s(C110)		2.227	14.884	< 0.001
s(C112)		2.303	45.970	< 0.001
s(C211)		0.603	1.347	0.112
s(C213)		0.955	14.573	< 0.001
s(C221)		0.974	26.401	< 0.001
s(C222)		0.779	3.577	0.031
s(C223)		0.001	0.000	0.937
s(C224)		0.001	0.000	0.788
s(C231)		1.656	85.363	< 0.001
s(C311)		2.446	135.150	< 0.001
s(C312)		1.017	26.918	< 0.001
s(C313)		2.138	65.853	< 0.001
s(C320)		0.926	11.379	< 0.001
s(C321)		0.654	2.098	0.072
s(C330)		0.750	2.873	0.049
s(C410)		0.013	0.009	0.402
s(C511)		0.775	3.409	0.032

Italian Sparrow (*Passer italiae*) — Model C-ZIP-GAM				
**Binomial component**				
**Parametric terms**	**Estimate**	**Standard error**	**z-value**	**p-value**
Intercept	6.307	0.180	35.120	< 0.001
**Smooth terms**		**edf**	**Chi square**	**p-value**
s(Elevation)		2.150	1884.500	< 0.001
s(Urb_{2500})		0.999	729.900	< 0.001
s(For_{2500})		0.000	0.000	0.552
**Count component**				
**Parametric terms**	**Estimate**	**Standard error**	**z-value**	**p-value**
Intercept	−0.322	0.052	−6.163	< 0.001
Year 1995	−0.351	0.058	−6.045	< 0.001
Year 1996	−0.534	0.056	−9.451	< 0.001
Year 1999	−0.073	0.055	−1.333	0.183
Year 2000	−0.268	0.052	−5.182	< 0.001
Year 2001	−0.560	0.056	−10.082	< 0.001
Year 2002	−0.393	0.064	−6.179	< 0.001
Year 2003	−0.593	0.068	−8.675	< 0.001
Year 2004	−0.621	0.077	−8.047	< 0.001
Year 2005	−0.643	0.055	−11.637	< 0.001
Year 2006	−0.791	0.057	−13.808	< 0.001
Year 2007	−0.916	0.064	−14.283	< 0.001
Year 2008	−0.916	0.064	−14.220	< 0.001
Year 2009	−1.003	0.063	−15.837	< 0.001
Year 2010	−0.998	0.064	−15.556	< 0.001
Year 2011	−1.119	0.063	−17.764	< 0.001
Year 2012	−1.157	0.068	−17.088	< 0.001
Year 2013	−1.245	0.067	−18.446	< 0.001
Year 2014	−1.388	0.067	−20.794	< 0.001
Year 2015	−1.177	0.065	−18.127	< 0.001
Year 2016	−1.355	0.071	−19.011	< 0.001
Year 2017	−1.145	0.078	−14.722	< 0.001
Year 2018	−0.977	0.073	−13.454	< 0.001
Year 2019	−1.017	0.073	−13.869	< 0.001
Sin	0.000	0.014	−0.034	0.973
Cos	0.000	0.022	0.010	0.992
**Smooth terms**		**edf**	**Chi square**	**p-value**
s(X, Y)		2.964	194.297	< 0.001
s(Elevation)		1.377	127.582	< 0.001
S(Slope)		1.065	25.331	< 0.001
s(C110)		2.300	179.201	< 0.001
s(C112)		2.965	655.634	< 0.001
s(C211)		1.323	33.441	< 0.001
s(C213)		2.153	54.436	< 0.001
s(C221)		2.490	17.050	< 0.001
s(C222)		0.758	3.026	0.044
s(C223)		0.931	9.306	0.001
s(C224)		1.584	3.588	0.093
s(C231)		1.845	71.784	< 0.001
s(C311)		2.905	171.857	< 0.001
s(C312)		1.000	66.285	< 0.001
s(C313)		2.198	94.083	< 0.001
s(C320)		0.959	29.269	< 0.001
s(C321)		1.836	3.547	0.129
s(C330)		1.635	8.853	0.005
s(C410)		0.897	13.400	< 0.001
s(C511)		2.695	6.356	0.068

**Eurasian Tree Sparrow (*Passer montanus*) — Model C-ZIP-GAM**				
**Binomial component**				
**Parametric terms**	**Estimate**	**Standard error**	**z-value**	**p-value**
Intercept	2.657	0.056	47.220	< 0.001
**Smooth terms**		**edf**	**Chi square**	**p-value**
s(Elevation)		1.274	1367.000	< 0.001
s(Urb_{2500})		2.987	1536.000	< 0.001
s(For_{2500})		2.992	1285.000	< 0.001
**Count component**				
**Parametric terms**	**Estimate**	**Standard error**	**z-value**	**p-value**
Intercept	−1.851	0.140	−13.218	< 0.001
Year 1995	−0.352	0.108	−3.245	0.001
Year 1996	−0.464	0.106	−4.387	< 0.001
Year 1999	−0.079	0.105	−0.756	0.449
Year 2000	−0.145	0.097	−1.498	0.134
Year 2001	−0.222	0.098	−2.274	0.023
Year 2002	−0.523	0.123	−4.253	< 0.001
Year 2003	−0.806	0.138	−5.845	< 0.001
Year 2004	−0.526	0.148	−3.557	< 0.001
Year 2005	−0.378	0.099	−3.828	< 0.001
Year 2006	−0.344	0.101	−3.415	< 0.001
Year 2007	−0.311	0.106	−2.945	0.003
Year 2008	−0.286	0.107	−2.681	0.007
Year 2009	−0.291	0.103	−2.835	0.005
Year 2010	−0.482	0.108	−4.444	< 0.001
Year 2011	−0.660	0.108	−6.092	< 0.001
Year 2012	−0.669	0.114	−5.889	< 0.001
Year 2013	−0.674	0.112	−5.998	< 0.001
Year 2014	−0.528	0.109	−4.862	< 0.001
Year 2015	−0.517	0.107	−4.821	< 0.001
Year 2016	−0.583	0.111	−5.256	< 0.001
Year 2017	−0.670	0.128	−5.224	< 0.001
Year 2018	−0.766	0.131	−5.853	< 0.001
Year 2019	−0.921	0.137	−6.706	< 0.001
Sin	0.025	0.023	1.085	0.278
Cos	0.064	0.035	1.811	0.070
**Smooth terms**		**edf**	**Chi square**	**p-value**
s(X, Y)		2.956	60.667	< 0.001
s(Elevation)		2.791	36.345	< 0.001
s(Slope)		1.047	25.221	< 0.001
s(C110)		2.252	15.416	< 0.001
s(C112)		2.931	120.242	< 0.001
s(C211)		1.069	18.045	< 0.001
s(C213)		1.121	32.702	< 0.001
s(C221)		1.890	13.353	< 0.001
s(C222)		0.877	5.564	0.011
s(C223)		0.000	0.000	0.531
s(C224)		1.610	1.741	0.314
s(C231)		1.717	20.534	< 0.001
s(C311)		2.146	50.230	< 0.001
s(C312)		2.784	23.823	< 0.001
s(C313)		2.059	32.807	< 0.001
s(C320)		0.000	0.000	0.304
s(C321)		0.002	0.000	0.903
s(C330)		0.742	3.555	0.028
s(C410)		0.897	13.829	< 0.001
s(C511)		1.905	4.068	0.105

**Common Chaffinch (*Fringilla coelebs*) — Model C-ZIP-GAM**				
**Binomial component**				
**Parametric terms**	**Estimate**	**Standard error**	**z-value**	**p-value**
Intercept	6.300	1.912	3.296	< 0.001
**Smooth terms**		**edf**	**Chi square**	**p-value**
s(Elevation)		0.003	0.000	1.000
s(Urb_{2500})		2.981	786.300	< 0.001
s(For_{2500})		1.639	1612.800	< 0.001
**Count component**				
**Parametric terms**	**Estimate**	**Standard error**	**z-value**	**p-value**
Intercept	0.205	0.050	4.072	< 0.001
Year 1995	−0.173	0.065	−2.672	0.008
Year 1996	−0.133	0.063	−2.093	0.036
Year 1999	−0.025	0.055	−0.455	0.649
Year 2000	−0.099	0.055	−1.817	0.069
Year 2001	−0.032	0.058	−0.547	0.584
Year 2002	−0.097	0.062	−1.553	0.120
Year 2003	−0.054	0.062	−0.873	0.383
Year 2004	−0.056	0.068	−0.823	0.411
Year 2005	0.014	0.058	0.238	0.812
Year 2006	0.025	0.056	0.440	0.660
Year 2007	0.019	0.060	0.319	0.750
Year 2008	0.011	0.061	0.174	0.862
Year 2009	0.008	0.061	0.131	0.896
Year 2010	−0.054	0.061	−0.884	0.377
Year 2011	−0.087	0.058	−1.483	0.138
Year 2012	−0.041	0.060	−0.681	0.496
Year 2013	−0.170	0.061	−2.779	0.005
Year 2014	−0.143	0.059	−2.397	0.017
Year 2015	−0.136	0.060	−2.254	0.024
Year 2016	−0.199	0.061	−3.270	0.001
Year 2017	−0.067	0.064	−1.054	0.292
Year 2018	−0.117	0.065	−1.813	0.070
Year 2019	−0.134	0.065	−2.063	0.039
Sin	−0.012	0.010	−1.142	0.254
Cos	0.009	0.015	0.584	0.559
**Smooth terms**		**edf**	**Chi square**	**p-value**
s(X, Y)		2.976	402.465	< 0.001
s(Elevation)		2.668	110.564	< 0.001
s(Slope)		2.697	66.233	< 0.001
s(C110)		1.508	1.714	0.241
s(C112)		1.764	3.218	0.125
s(C211)		2.893	31.125	< 0.001
s(C213)		0.907	17.705	< 0.001
s(C221)		2.883	23.012	< 0.001
s(C222)		1.738	3.473	0.125
s(C223)		1.781	10.761	0.002
s(C224)		1.423	24.062	< 0.001
s(C231)		0.483	2.811	0.007
s(C311)		2.462	97.402	< 0.001
s(C312)		2.860	209.555	< 0.001
s(C313)		2.461	141.634	< 0.001
s(C320)		0.697	3.709	0.011
s(C321)		2.949	136.362	< 0.001
s(C330)		0.977	57.090	< 0.001
s(C410)		0.912	13.229	< 0.001
s(C511)		2.808	9.517	0.017

**European Serin (*Serinus serinus*) — Model C-ZIP-GAM**				
**Binomial component**				
**Parametric terms**	**Estimate**	**Standard error**	**z-value**	**p-value**
Intercept	0.357	0.026	13.560	< 0.001
**Smooth terms**		**edf**	**Chi square**	**p-value**
s(Elevation)		2.999	2188.000	< 0.001
s(Urb_{2500})		2.989	1835.000	< 0.001
s(For_{2500})		2.985	1446.000	< 0.001
**Count component**				
**Parametric terms**	**Estimate**	**Standard error**	**z-value**	**p-value**
Intercept	−1.300	0.114	−11.431	< 0.001
Year 1995	−0.476	0.147	−3.234	0.001
Year 1996	−0.802	0.154	−5.203	< 0.001
Year 1999	−0.271	0.135	−2.016	0.044
Year 2000	−0.037	0.121	−0.308	0.758
Year 2001	−0.389	0.136	−2.865	0.004
Year 2002	−0.126	0.146	−0.867	0.386
Year 2003	−0.378	0.154	−2.447	0.014
Year 2004	−0.188	0.164	−1.146	0.252
Year 2005	−0.192	0.126	−1.525	0.127
Year 2006	−0.088	0.122	−0.719	0.472
Year 2007	−0.203	0.130	−1.555	0.120
Year 2008	−0.159	0.129	−1.232	0.218
Year 2009	−0.113	0.129	−0.874	0.382
Year 2010	−0.241	0.129	−1.875	0.061
Year 2011	−0.463	0.129	−3.598	< 0.001
Year 2012	−0.231	0.129	−1.790	0.073
Year 2013	−0.524	0.137	−3.824	< 0.001
Year 2014	−0.555	0.128	−4.320	< 0.001
Year 2015	−0.565	0.134	−4.221	< 0.001
Year 2016	−0.448	0.133	−3.380	< 0.001
Year 2017	−0.440	0.147	−2.997	0.003
Year 2018	−0.333	0.143	−2.329	0.020
Year 2019	−0.317	0.141	−2.250	0.024
Sin	−0.050	0.026	−1.894	0.058
Cos	−0.079	0.042	−1.878	0.060
**Smooth terms**		**edf**	**Chi square**	**p-value**
s(X, Y)		2.988	< 0.001	< 0.001
s(Elevation)		0.001	0.000	0.604
s(Slope)		2.895	113.883	< 0.001
s(C110)		2.386	121.305	< 0.001
s(C112)		1.746	67.878	< 0.001
s(C211)		0.542	0.944	0.164
s(C213)		0.988	29.669	< 0.001
s(C221)		2.895	110.787	< 0.001
s(C222)		0.909	16.834	< 0.001
s(C223)		0.721	3.434	0.026
s(C224)		0.675	1.737	0.106
s(C231)		2.081	8.550	0.005
s(C311)		2.886	118.367	< 0.001
s(C312)		1.860	31.369	< 0.001
s(C313)		2.228	63.291	< 0.001
s(C320)		0.773	2.389	0.071
s(C321)		1.911	7.472	0.015
s(C330)		1.915	15.494	< 0.001
s(C410)		0.954	11.390	< 0.001
s(C511)		1.291	0.428	0.665

**European Greenfinch (*Chloris chloris*) — Model C-ZINB-GAM**				
**Binomial component**				
**Parametric terms**	**Estimate**	**Standard error**	**z-value**	**p-value**
Intercept	−0.146	0.019	−7.711	< 0.001
**Smooth terms**		**edf**	**Chi square**	**p-value**
s(Elevation)		2.999	1202.150	< 0.001
s(Urb_{2500})		2.978	1058.550	< 0.001
s(For_{2500})		1.262	54.350	< 0.001
**Count component**				
**Parametric terms**	**Estimate**	**Standard error**	**z-value**	**p-value**
Intercept	−0.632	0.109	−5.807	< 0.001
Year 1995	−0.378	0.121	−3.118	0.002
Year 1996	−0.627	0.122	−5.143	< 0.001
Year 1999	−0.084	0.115	−0.733	0.464
Year 2000	−0.024	0.106	−0.226	0.821
Year 2001	−0.659	0.118	−5.569	< 0.001
Year 2002	−0.634	0.137	−4.641	< 0.001
Year 2003	−0.767	0.141	−5.426	< 0.001
Year 2004	−1.120	0.176	−6.369	< 0.001
Year 2005	−1.016	0.121	−8.411	< 0.001
Year 2006	−1.107	0.122	−9.098	< 0.001
Year 2007	−1.093	0.134	−8.154	< 0.001
Year 2008	−0.987	0.129	−7.631	< 0.001
Year 2009	−1.450	0.145	−10.017	< 0.001
Year 2010	−1.443	0.144	−10.047	< 0.001
Year 2011	−1.255	0.129	−9.746	< 0.001
Year 2012	−0.986	0.129	−7.644	< 0.001
Year 2013	−1.446	0.144	−10.072	< 0.001
Year 2014	−1.582	0.136	−11.645	< 0.001
Year 2015	−1.429	0.137	−10.428	< 0.001
Year 2016	−1.658	0.151	−11.019	< 0.001
Year 2017	−1.592	0.168	−9.460	< 0.001
Year 2018	−1.973	0.191	−10.323	< 0.001
Year 2019	−1.691	0.173	−9.750	< 0.001
Sin	0.058	0.030	1.966	0.049
Cos	−0.061	0.046	−1.329	0.184
**Smooth terms**		**edf**	**Chi square**	**p-value**
s(X, Y)		2.984	329.850	< 0.001
s(Elevation)		2.704	19.338	< 0.001
s(Slope)		0.998	9.789	< 0.001
s(C110)		2.500	125.367	< 0.001
s(C112)		0.944	12.391	< 0.001
s(C211)		0.825	3.710	0.027
s(C213)		0.982	22.378	< 0.001
s(C221)		0.936	13.978	< 0.001
s(C222)		0.028	0.029	0.305
s(C223)		0.902	9.298	0.001
s(C224)		0.920	10.673	< 0.001
s(C231)		0.001	0.000	0.633
s(C311)		2.713	123.871	< 0.001
s(C312)		0.998	21.063	< 0.001
s(C313)		1.033	33.425	< 0.001
s(C320)		0.000	0.000	0.845
s(C321)		0.333	0.470	0.231
s(C330)		0.000	0.000	0.903
s(C410)		0.933	12.960	< 0.001
s(C511)		0.693	2.180	0.075

**European Goldfinch (*Carduelis carduelis*) — Model C-ZINB-GAM**				
**Binomial component**				
**Parametric terms**	**Estimate**	**Standard error**	**z-value**	**p-value**
Intercept	0.065	0.019	3.525	< 0.001
**Smooth terms**		**edf**	**Chi square**	**p-value**
s(Elevation)		2.941	1045.600	< 0.001
s(Urb_{2500})		2.984	181.300	< 0.001
s(For_{2500})		2.952	59.300	< 0.001
**Count component**				
**Parametric terms**	**Estimate**	**Standard error**	**z-value**	**p-value**
Intercept	−0.281	0.099	−2.850	0.004
Year 1995	−0.638	0.125	−5.087	< 0.001
Year 1996	−0.316	0.115	−2.745	0.006
Year 1999	−0.168	0.112	−1.500	0.134
Year 2000	−0.131	0.107	−1.225	0.221
Year 2001	−0.298	0.112	−2.670	0.008
Year 2002	−0.503	0.130	−3.871	< 0.001
Year 2003	−0.450	0.129	−3.497	< 0.001
Year 2004	−0.728	0.153	−4.745	< 0.001
Year 2005	−0.728	0.114	−6.369	< 0.001
Year 2006	−1.077	0.123	−8.789	< 0.001
Year 2007	−1.136	0.136	−8.377	< 0.001
Year 2008	−1.247	0.139	−8.958	< 0.001
Year 2009	−1.447	0.145	−9.992	< 0.001
Year 2010	−1.541	0.148	−10.402	< 0.001
Year 2011	−1.676	0.144	−11.677	< 0.001
Year 2012	−1.289	0.135	−9.517	< 0.001
Year 2013	−1.268	0.134	−9.449	< 0.001
Year 2014	−1.549	0.139	−11.151	< 0.001
Year 2015	−1.373	0.136	−10.064	< 0.001
Year 2016	−1.532	0.141	−10.846	< 0.001
Year 2017	−1.458	0.159	−9.188	< 0.001
Year 2018	−1.871	0.181	−10.326	< 0.001
Year 2019	−1.947	0.184	−10.578	< 0.001
Sin	−0.023	0.028	−0.845	0.398
Cos	0.005	0.041	0.125	0.901
**Smooth terms**		**edf**	**Chi square**	**p-value**
s(X, Y)		1.934	85.697	< 0.001
s(Elevation)		2.717	68.253	< 0.001
s(Slope)		2.561	35.419	< 0.001
s(C110)		2.540	79.730	< 0.001
s(C112)		1.005	23.050	< 0.001
s(C211)		0.967	6.051	0.007
s(C213)		0.001	0.001	0.396
s(C221)		0.934	13.401	< 0.001
s(C222)		0.074	0.080	0.296
s(C223)		0.352	0.571	0.202
s(C224)		0.001	0.001	0.403
s(C231)		1.850	35.231	< 0.001
s(C311)		2.677	119.049	< 0.001
s(C312)		1.185	56.521	< 0.001
s(C313)		2.428	57.803	< 0.001
s(C320)		0.897	7.960	0.003
s(C321)		0.291	0.398	0.238
s(C330)		0.003	0.001	0.530
s(C410)		0.873	6.535	0.006
s(C511)		0.801	4.036	0.023

Common Linnet (*Linaria cannabina*) — Model C-ZINB-GAM				
**Binomial component**				
**Parametric terms**	**Estimate**	**Standard error**	**z-value**	**p-value**
Intercept	−2.930	0.044	−67.050	< 0.001
**Smooth terms**		**edf**	**Chi square**	**p-value**
s(Elevation)		2.686	3830.038	< 0.001
s(Urb_{2500})		0.000	0.000	0.946
s(For_{2500})		0.500	2.865	0.011
**Count component**				
**Parametric terms**	**Estimate**	**Standard error**	**z-value**	**p-value**
Intercept	−9.612	20.227	−0.475	0.635
Year 1996	−1.059	0.633	−1.672	0.094
Year 1999	−0.150	0.439	−0.341	0.733
Year 2000	−0.131	0.410	−0.319	0.750
Year 2001	−0.413	0.465	−0.888	0.374
Year 2002	−0.373	0.473	−0.790	0.429
Year 2003	−0.109	0.429	−0.254	0.799
Year 2004	−0.727	0.500	−1.454	0.146
Year 2005	0.552	0.388	1.423	0.155
Year 2006	0.233	0.414	0.564	0.573
Year 2007	−0.073	0.420	−0.175	0.861
Year 2008	0.183	0.406	0.449	0.653
Year 2009	−0.096	0.420	−0.228	0.820
Year 2010	−0.268	0.421	−0.637	0.524
Year 2011	0.278	0.401	0.692	0.489
Year 2012	0.150	0.402	0.373	0.709
Year 2013	−0.115	0.411	−0.280	0.780
Year 2014	−0.227	0.410	−0.553	0.580
Year 2015	−0.205	0.409	−0.501	0.616
Year 2016	−0.460	0.419	−1.098	0.272
Year 2017	−0.372	0.423	−0.880	0.379
Year 2018	−0.417	0.430	−0.969	0.333
Year 2019	0.117	0.408	0.286	0.775
Sin	−0.114	0.071	−1.598	0.110
Cos	−0.072	0.114	−0.635	0.525
**Smooth terms**		**edf**	**Chi square**	**p-value**
s(X, Y)		1.944	56.401	< 0.001
s(Elevation)		2.730	98.546	< 0.001
s(Slope)		1.882	5.297	0.049
s(C110)		0.000	0.000	0.634
s(C112)		2.049	16.564	< 0.001
s(C211)		0.000	0.000	0.415
s(C213)		0.705	0.012	0.899
s(C221)		0.940	13.156	< 0.001
s(C222)		0.000	0.000	0.930
s(C223)		0.000	0.000	0.827
s(C224)		0.267	0.056	0.646
s(C231)		0.000	0.000	0.850
s(C311)		1.090	22.332	< 0.001
s(C312)		1.964	79.921	< 0.001
s(C313)		1.038	32.620	< 0.001
s(C320)		2.260	15.396	< 0.001
s(C321)		0.000	0.000	0.940
s(C330)		0.984	32.762	< 0.001
s(C410)		0.002	0.001	0.383
s(C511)		0.823	4.278	0.022

**Common Redpoll (*Acanthis flammea*) — Model C-ZINB-GAM**				
**Binomial component**				
**Parametric terms**	**Estimate**	**Standard error**	**z-value**	**p-value**
Intercept	−3.551	0.067	−53.160	< 0.001
**Smooth terms**		**edf**	**Chi square**	**p-value**
s(Elevation)		2.892	2178.420	< 0.001
s(Urb_{2500})		0.000	0.000	0.929
s(For_{2500})		2.306	42.360	< 0.001
**Count component**				
**Parametric terms**	**Estimate**	**Standard error**	**z-value**	**p-value**
Intercept	−7.041	25.421	−0.277	0.782
Year 1995	0.295	0.547	0.539	0.590
Year 1996	0.634	0.551	1.152	0.249
Year 1999	−0.609	0.562	−1.085	0.278
Year 2000	0.423	0.528	0.801	0.423
Year 2001	−0.185	0.548	−0.337	0.736
Year 2002	0.314	0.548	0.574	0.566
Year 2003	−0.752	0.557	−1.349	0.177
Year 2004	0.155	0.542	0.285	0.775
Year 2005	0.206	0.518	0.397	0.691
Year 2006	0.349	0.541	0.645	0.519
Year 2007	−0.378	0.543	−0.697	0.486
Year 2008	−0.574	0.544	−1.055	0.291
Year 2009	−0.701	0.549	−1.276	0.202
Year 2010	−0.875	0.561	−1.560	0.119
Year 2011	−0.022	0.527	−0.042	0.967
Year 2012	0.173	0.521	0.333	0.739
Year 2013	−0.154	0.530	−0.291	0.771
Year 2014	0.013	0.523	0.026	0.979
Year 2015	0.085	0.518	0.163	0.870
Year 2016	−0.586	0.537	−1.090	0.276
Year 2017	−0.551	0.544	−1.014	0.311
Year 2018	−0.902	0.566	−1.595	0.111
Year 2019	−0.641	0.549	−1.168	0.243
Sin	−0.124	0.064	−1.929	0.054
Cos	0.344	0.090	3.811	< 0.001
**Smooth terms**		**edf**	**Chi square**	**p-value**
s(X, Y)		1.783	16.389	< 0.001
s(Elevation)		2.496	34.507	< 0.001
s(Slope)		2.535	25.271	< 0.001
s(C110)		0.002	0.001	0.413
s(C112)		0.000	0.000	0.400
s(C211)		0.597	0.743	0.259
s(C213)		0.000	0.000	0.860
s(C221)		0.000	0.000	0.624
s(C222)		0.000	0.000	0.852
s(C223)		0.000	0.000	0.902
s(C224)		0.227	0.013	0.810
s(C231)		0.000	0.000	1.000
s(C311)		0.000	0.000	0.881
s(C312)		2.209	28.429	< 0.001
s(C313)		0.831	4.305	0.021
s(C320)		2.035	7.829	0.013
s(C321)		2.198	18.131	< 0.001
s(C330)		0.529	1.112	0.142
s(C410)		0.000	0.000	0.605
s(C511)		0.001	0.000	0.446

Eurasian Bullfinch (*Pyrrhula pyrrhula*) — Model C-ZIP-GAM				
**Binomial component**				
**Parametric terms**	**Estimate**	**Standard error**	**z-value**	**p-value**
Intercept	−2.553	0.039	−64.760	< 0.001
**Smooth terms**		**edf**	**Chi square**	**p-value**
s(Elevation)		2.996	2722.820	< 0.001
s(Urb_{2500})		0.002	0.000	1.000
s(For_{2500})		2.006	97.430	< 0.001
**Count component**				
**Parametric terms**	**Estimate**	**Standard error**	**z-value**	**p-value**
Intercept	−6.245	3.029	−2.061	0.039
Year 1995	1.299	0.514	2.529	0.011
Year 1996	−0.663	0.734	−0.903	0.367
Year 1999	0.845	0.478	1.770	0.077
Year 2000	0.961	0.469	2.048	0.041
Year 2001	1.529	0.469	3.259	0.001
Year 2002	1.383	0.477	2.898	0.004
Year 2003	1.080	0.486	2.224	0.026
Year 2004	1.254	0.510	2.461	0.014
Year 2005	1.019	0.480	2.124	0.034
Year 2006	0.209	0.551	0.380	0.704
Year 2007	0.293	0.519	0.563	0.573
Year 2008	1.248	0.481	2.595	0.009
Year 2009	0.517	0.523	0.987	0.323
Year 2010	1.083	0.489	2.216	0.027
Year 2011	1.275	0.478	2.668	0.008
Year 2012	1.071	0.491	2.181	0.029
Year 2013	0.780	0.496	1.574	0.115
Year 2014	0.699	0.498	1.402	0.161
Year 2015	1.051	0.487	2.161	0.031
Year 2016	0.992	0.487	2.038	0.042
Year 2017	0.822	0.516	1.594	0.111
Year 2018	0.599	0.530	1.131	0.258
Year 2019	0.653	0.530	1.234	0.217
Sin	−0.008	0.056	−0.148	0.882
Cos	0.069	0.079	0.879	0.380
**Smooth terms**		**edf**	**Chi square**	**p-value**
s(X, Y)		1.918	55.513	< 0.001
s(Elevation)		2.835	34.892	< 0.001
s(Slope)		0.931	8.684	0.002
s(C110)		1.939	14.939	< 0.001
s(C112)		0.962	14.599	< 0.001
s(C211)		0.821	3.303	0.042
s(C213)		0.001	0.000	0.595
s(C221)		0.647	0.327	0.477
s(C222)		0.609	0.040	0.797
s(C223)		0.001	0.000	0.820
s(C224)		0.000	0.000	0.706
s(C231)		1.426	33.316	< 0.001
s(C311)		1.013	13.524	< 0.001
s(C312)		2.459	28.298	< 0.001
s(C313)		0.000	0.000	0.386
s(C320)		2.637	14.036	0.001
s(C321)		2.823	44.499	< 0.001
s(C330)		1.774	11.105	0.002
s(C410)		0.000	0.000	0.507
s(C511)		0.882	7.720	0.003

Figure S2. Plot of population indices with confidence intervals and regression line for the 76 studied species. Y-axis represents estimated number of breeding pairs per point count (see Section 2.3 for details). Dot and bars represent the median and the 2.5th-97.5th percentiles of 1000 bootstrapped values, respectively. Fitted WLS-regression lines with confidence intervals (shaded area) are superimposed.

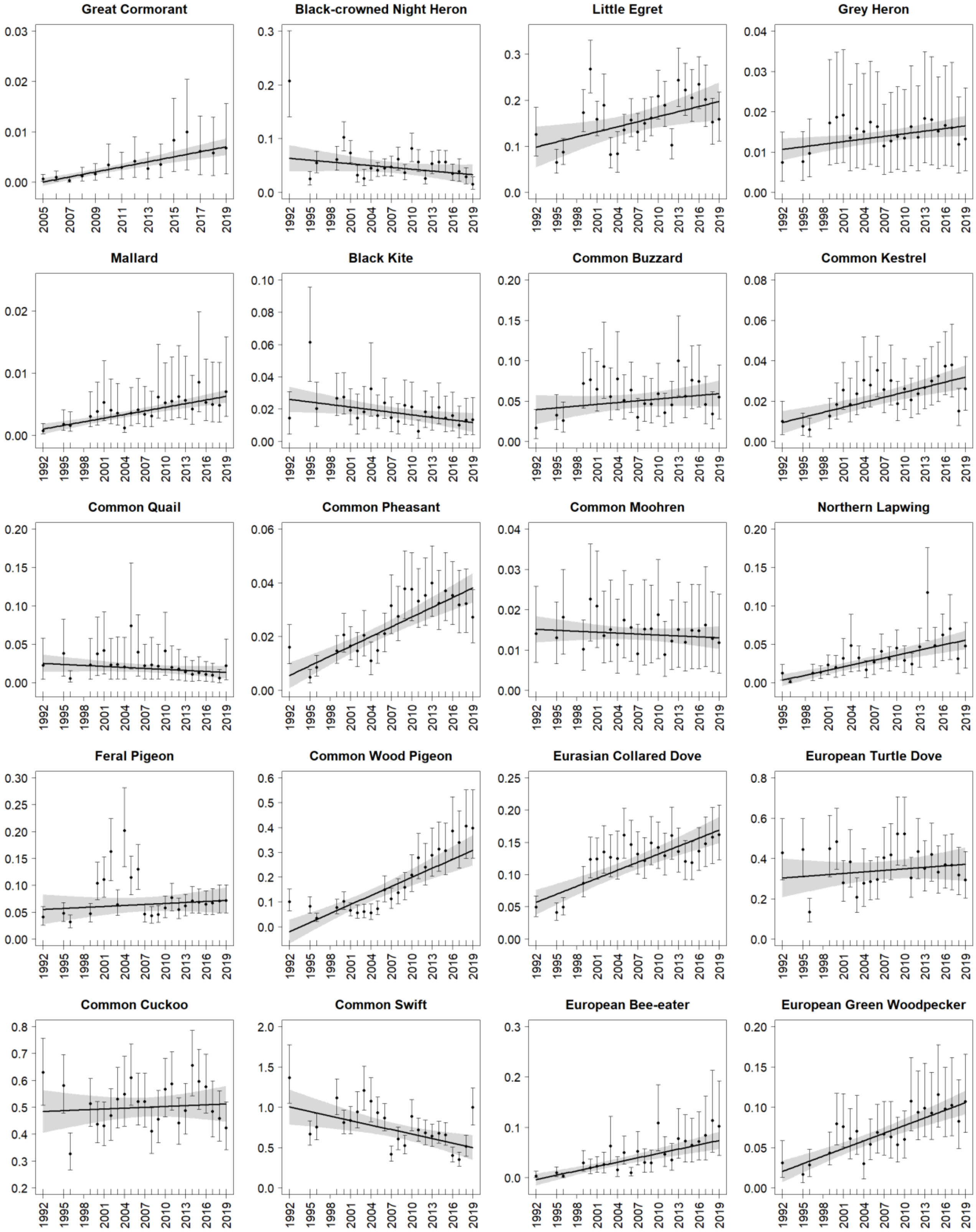

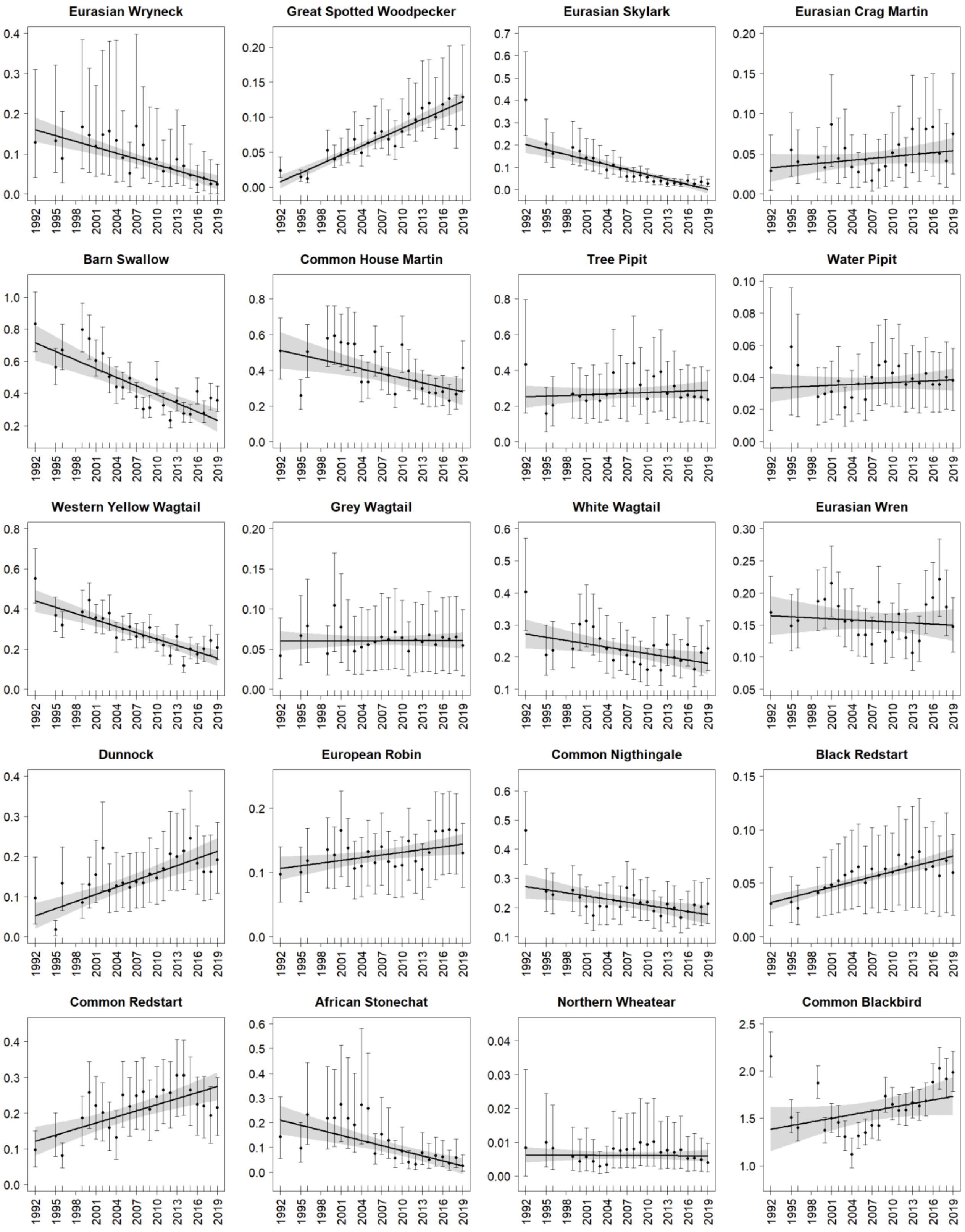
Eurasian Wryneck
Great Spotted Woodpecker
Eurasian Skylark
Eurasian Crag Martin
Barn Swallow
Common House Martin
Tree Pipit
Water Pipit
Western Yellow Wagtail
Grey Wagtail
White Wagtail
Eurasian Wren
Dunnock
European Robin
Common Nigthingale
Black Redstart
Common Redstart
African Stonechat
Northern Wheatear
Common Blackbird

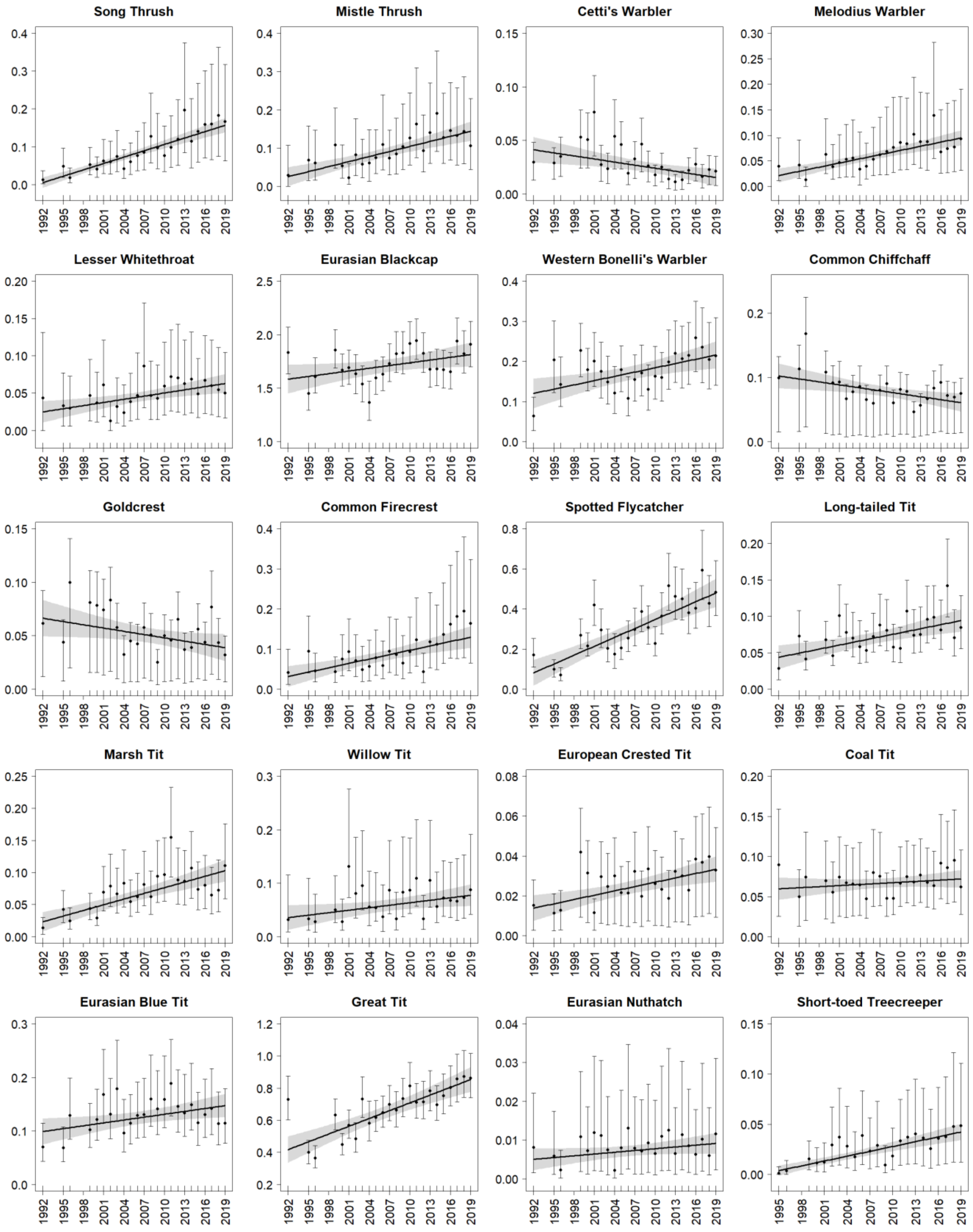
Song Thrush
Mistle Thrush
Cetti's Warbler
Melodius Warbler
Lesser Whitethroat
Eurasian Blackcap
Western Bonelli's Warbler
Common Chiffchaff
Goldcrest
Common Firecrest
Spotted Flycatcher
Long-tailed Tit
Marsh Tit
Willow Tit
European Crested Tit
Coal Tit
Eurasian Blue Tit
Great Tit
Eurasian Nuthatch
Short-toed Treecreeper

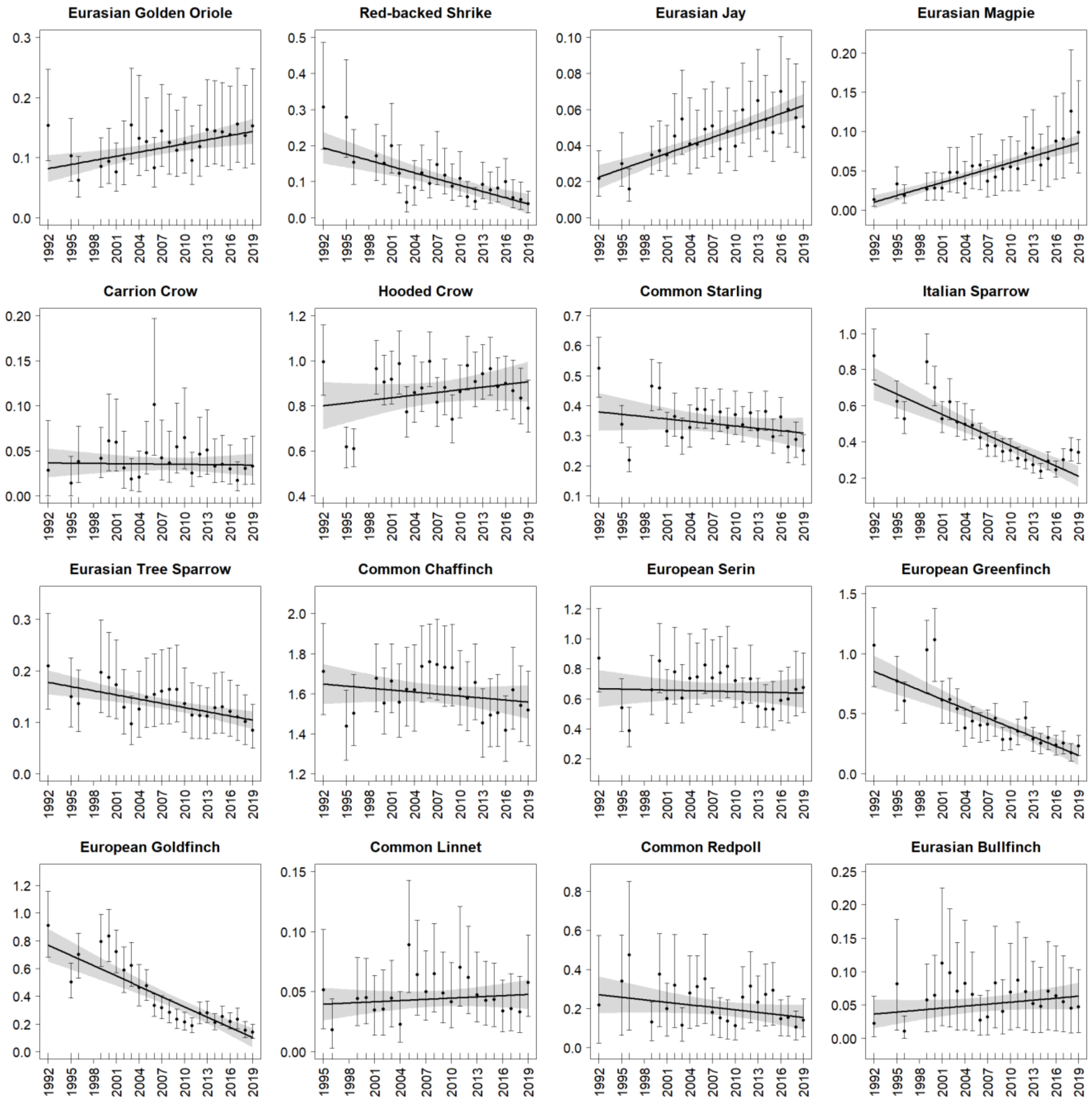
Eurasian Golden Oriole
Red-backed Shrike
Eurasian Jay
Eurasian Magpie
Carrion Crow
Hooded Crow
Common Starling
Italian Sparrow
Eurasian Tree Sparrow
Common Chaffinch
European Serin
European Greenfinch
European Goldfinch
Common Linnet
Common Redpoll
Eurasian Bullfinch
